# Prevalence of fungal colonization among patients with psoriasis in difficult-to-treat areas: impact of apremilast on mycotic burden and clinical outcomes

**DOI:** 10.3389/fimmu.2024.1508489

**Published:** 2024-12-10

**Authors:** Elena Campione, Terenzio Cosio, Enrico Salvatore Pistoia, Fabio Artosi, Ruslana Gaeta Shumack, Cristiana Borselli, Antonia Rivieccio, Valerio Caputo, Marco Favaro, Roberto Sorge, Francesca Pica, Luca Bianchi, Roberta Gaziano

**Affiliations:** ^1^ Dermatologic Unit, Department of Systems Medicine, University of Rome Tor Vergata, Rome, Italy; ^2^ Department of Experimental Medicine, University of Rome “Tor Vergata”, Rome, Italy; ^3^ Genomic Medicine Laboratory UILDM, IRCCS Santa Lucia Foundation, Rome, Italy; ^4^ Department of Biostatistics, Tor Vergata University of Rome, Rome, Italy

**Keywords:** apremilast, difficult-to-treat psoriasis areas, *Candida* species, fungal infections, cytokines, IL-17

## Abstract

**Introduction:**

Fungi, including *Candida*, may be a trigger or exacerbate psoriasis, especially in difficult to treat (DTT) areas, through the activation of IL-17/23 axis.

**Methods:**

In this study, seventy patients with DDT psoriasis were enrolled to evaluate *Candida* species and/or other opportunistic fungi colonization rate at baseline (T0) and the impact of apremilast on fungal load, clinical outcome, serum cytokine levels and biochemical serum profile of patients after 16, 24 and 52 weeks of treatment.

**Results:**

In our population, 33 (47%) patients were colonized by *Candida* spp. at baseline. In 24 (34%) individuals *Candida* was detected in the oral cavity while in the remaining 9 (13%) individuals the fungus was isolated from stool samples. Twenty subjects were colonized by only the species *C. albicans*, whereas in the remaining 13 a combination of two or more species (*C. albicans* plus non-*albicans* strains) was found in the oral cavity. Moreover, 27 (39%) patients were affected by onychomycosis. At 52 weeks, apremilast treatment induced a full recovery from *Candida* colonization in 83% of patients colonized with a single species of *Candida* (*C. albicans*); while in those co-infected by two or more *Candida* spp. induced a significant reduction (colony counts >10 CFU/mL) in fungal load was observed in comparison to baseline. Among patients with onychomycosis, 78% (21/27) of them presented a complete clinical resolution of nail psoriasis and concomitant nail infections. Finally, improvements in clinical scores i.e., PASI, NAPSI, DLQI, itch VAS, PAIN VAS, scPGA and sPGA-G and biochemical serum profile, as well as a significant decrease in serum IL-17A, TGF-β 1 and IL-10 levels (from 8.51 to 4.16 pg/mL; from 66.10 to 48.70 ng/mL and from 20.05 to 14 pg/mL, respectively) were observed in all patients.

**Conclusions:**

Fungi may play a role in the psoriasis pathogenesis. Apremilast has been shown to ameliorate psoriasis signs and symptoms and counteract fungal overgrowth, probably by dampening inflammation, triggered by the fungal infections themselves. Thus, apremilast may represent an effective therapeutic approach in the treatment of DTT psoriasis and modulate the fungal colonization.

## 1 Introduction

Psoriasis (PsO) is a complex chronic, autoimmune-inflammatory disorder, affecting approximately 2% of the general population, mainly involving the skin, although it is considered a systemic disease, which can be coupled with psoriatic arthritis (PsA) (1–3). The trunk and extremities are the most commonly affected areas in plaque psoriasis, but nails, scalp and intertriginous areas, including groins, abdominal skin folds, inframammary folds, and interdigital spaces can be involved (4). The involvement of these areas has a significant impact on quality of life (QoL) with severe negative physical, psychological and psychosocial effects for psoriatic patients (5). Psoriasis that affects these areas of the skin is often referred to as “difficult-to-treat” (6) and it is usually more resistant to traditional therapies than other psoriatic clinical variants, and normally these clinical features are present simultaneously (6–8). Dysregulation of the immune system, as well as abnormal keratinocyte proliferation and differentiation, seem to play an important role in PsO pathogenesis (9). In terms of the implications for patients and clinicians, PsO is associated with an increased risk of serious infections, especially respiratory and soft‐tissue/skin infections, underlining the burden of infective comorbidities in psoriatic patients (10). Moreover, several evidence have indicated that infections are an environmental trigger for PsO and may play multiple roles in its maintenance, as evidenced by the frequent association between guttate psoriasis onset and acute streptococcal infection (11). Various bacterial, viral and fungal pathogens may play a role as superantigens that trigger the immune cells to produce inflammatory cytokines, leading to the onset or exacerbation of psoriasis (10). Moreover, over the past several years, many studies confirmed the association between gut and/or skin dysbiosis and psoriasis (12–14). In this regard, increasing evidence suggests that gut microbiota plays a central role in the maintenance of host immune homeostasis. Alterations in microbiota composition and diversity have been proposed to contribute to PsO pathogenicity in a susceptible host with sub-clinical inflammation by triggering an exaggerated immune response (15–21). Notably, *Candida* species overgrowth in gut dysbiosis may play an important role in the pathogenesis of psoriasis. Several molecular pathways associated with psoriasis and other inflammatory diseases, such as the interleukin (IL)-23/IL-17 axis, are also involved in host defense mechanisms against fungal pathogens (22). The Interleukin-17 family plays a key role in host defenses against certain pathogenic fungi including *Candida*. Elevated serum levels of the proinflammatory IL-17 have been documented in psoriatic patients and a positive correlation was found between serum concentration of IL-17 and psoriasis severity, suggesting its implication in psoriasis pathogenicity (23). It is assumed that high colonization level of *C. albicans* in the gut leads to the release of *Candida* superantigens, contributing to non-specific T-cell activation and excessive production of pro-inflammatory cytokines, which can act as trigger factors of psoriatic disease (24). This may create a “vicious cycle” in which inflammation promotes *Candida* colonization, and fungal colonization further improves inflammation. Significant levels of C. *albicans* detected in saliva and stool samples of psoriatic patients, reinforce the hypothesis that *C. albicans* may trigger both exacerbation and persistence of psoriasis (15). Even though increasing evidence suggests the implication of *Candida* in the complex pathophysiology of psoriasis, there is no knowledge of the prevalence of *Candida* in patients with concomitant nail, scalp and inverse psoriasis. Further, the association between *Candida* colonization rates and psoriasis severity still remains controversial (25, 26). Additionally, despite significant advances in knowledge of psoriasis pathogenesis have been made over the years, there is not yet a complete recovery of psoriasis, especially in DTT psoriasis and multifailure patients (27). A major challenge addresses DTT areas, especially in those individuals with the absence of significant body surface involvement (26). Moreover, recent data indicate that psoriasis is associated with an increased risk of cardiovascular disease and related comorbidities such as diabetes mellitus, metabolic syndrome, dyslipidaemia, and obesity (28). The common factor is a low-grade chronic inflammation and the increases in systemic oxidation that favor a vicious circle. Although psoriasis is considered a systemic disease (3), topical treatments are commonly used to treat mild-to-moderate psoriasis and have shown efficacy and tolerability in randomized clinical trials (29, 30). However, recent guidelines suggest systemic treatments for patients with mild-to-moderate skin involvement who experience high disease burden or inadequate disease control with topical therapies, as well as with PsO in special areas (31). On the other hand, topical corticosteroid can cause contact allergy, especially in sensible body area, and favor an increase in fungal infections as mucocutaneous candidiasis, when applied on genital area (32–34). Among systemic therapies, IL-17 inhibitors, have shown therapeutic benefits in treating moderate/severe plaque psoriasis (7, 35). Notably, ixekizumab, an inhibitor of interleukin-17A, has shown to be effective and safe in the treatment of moderate-to-severe genital PsO (7, 35). Likewise genital and scalp PsO is a major issue to be researched, as it is not only a physical burden, but at the same time inflicts significant psychological stress on the patient, disproportional to the body area affected (36). Nevertheless, due to the protective role of IL-17 against fungal infections, these drugs have been associated with an increased risk of mucocutaneous candidiasis in PsO patients (37–39). Besides IL-17 inhibitors, recent reports documented the efficacy of apremilast, a selective phosphodiesterase 4 (PDE4) inhibitor, in treating psoriasis (40, 41). In 2023, a double-blinded placebo-controlled trial demonstrated the effectiveness of apremilast in the treatment of genital PsO (42), other than special areas, such as scalp, nails, and palmoplantar areas (43). Based on this evidence, the present study aimed at evaluating: i) the prevalence of *Candida* species and other opportunistic fungi in psoriatic patients with DTT and the impact of apremilast on *Candida* colonization rates; ii) the clinical efficacy of apremilast in the study population iii) the effect of apremilast on serum cytokine and biochemical profiles to provide new insights into the beneficial effects of this drug in psoriatic disease.

## 2 Material and methods

### 2.1 Patient enrolment and study design

A pivotal, prospective, single-center study was performed to evaluate the prevalence of fungal colonization in DTT psoriatic patients treated with apremilast (30 mg bid, orally). Seventy patients were enrolled in this study and followed for 52 weeks. *Candida* colonization rates were documented by cultural examination and expressed as colony-forming units (CFU). Patients with active fungal infection (regardless of baseline presence or development during the study) were not excluded but received an antifungal treatment as in common clinical practice. We regularly follow international guidelines, which include the following treatments: for mild disease, clotrimazole, ciclopirox or miconazole; for moderate to severe disease, oral fluconazole (20). Psoriasis Area Severity Index (PASI), Nail Psoriasis Severity Index (NAPSI), Pain VAS, Itch VAS, Dermatology Life Quality Index (DLQI), and Scalp Physician Global Assessment (scPGA) and Genital PGA (sPGA-G) scores were collected for each patient at baseline (T0), 16, 24 and 52-weeks (W). Moreover, all the patients were evaluated for serum cytokine levels, as described below. This study was conducted following the Declaration of Helsinki and approved by the Institutional Review Board’s (IRB) Independent Ethical Committee Tor Vergata University Hospital (R.S. 133.19).

### 2.2 Inclusion and exclusion criteria

Inclusion criteria comprised patients who have undergone cultural examination for fungal infections within the last 15 days before the enrolment; patients ≥18 years of age and affected by psoriasis with concomitant nail, scalp and intertriginous disease; patients who were consecutively enrolled after having received apremilast, and accordingly had to meet all the requested conditions to be treated with apremilast; signed and dated written informed consent; appropriate wash out from previous systemic and biological therapies from at least 3 months for psoriasis and fungal infections. Exclusion criteria comprised pregnancy, breast-feeding, or female who planned to become pregnant while in the trial; underage; galactose-intolerant; diagnosed anorexia; currently enrolled in another investigational device or drug study, or less than 30 days since ending another investigational device or drug study(s) or receiving other investigational treatment; previous treatment with IL-17 inhibitors.

### 2.3 Primary objective

The primary objective of this study was to investigate the skin, oral mucosa and gut *Candida* spp. prevalence in patients affected by nail, scalp and inverse psoriasis at baseline (T0) and the effect of apremilast on *Candida* colonization at 16 (16W), 24 (24W) and 52 (52W) weeks of treatment.

### 2.4 Secondary objectives

The secondary objective was to evaluate the efficacy, safety and tolerability of apremilast in patients with simultaneous involvement of DTT areas (nail, scalp and folds) by evaluating changes in PASI, NAPSI, DLQI, itch VAS, PAIN VAS, scPGA and sPGA-G scores at week 0, 16, 24 and 52. Moreover, we explored whether *Candida* colonization negatively impacted the patient’s outcomes. The safety and tolerability of apremilast were evaluated throughout the study (including any adverse events (AEs), as reported by patients during follow-up examinations at 16, 24 and 52 weeks of treatment.

### 2.5 Tertiary objectives

The tertiary objective of this study was to investigate the impact of apremilast on serum cytokine levels (IL-17A, IL-10 and TGF-β 1) and biochemical serum profile in our population.

### 2.6 Sampling and isolation of *Candida* species and other fungi

The presence of *Candida* spp. in the skin folds (axilla, intergluteal, infra-mammary, and genitocrural fold), scalp, oral cavity and stools was assessed by cultural examination at baseline and 16, 24 and 52 weeks after treatment with apremilast. Swabs (eSwab™; Copan, Italy) were sampled from psoriatic lesions and the oral cavity and collected in sterile polystyrene tubes (Copan spa, Brescia, Italy). One stool sample and three nail clipping were also collected from each patient. Specifically, *Candida* gut colonization was achieved by diluting 100 µL of stool sample (approx. 100 µg) in 900 µL of PBS, plating 100 µL on Sabouraud Dextrose Agar (SDA) supplemented with chloramphenicol (Difco Laboratories, Detroit, MI, USA); *Candida* oral colonization was achieved by plating 100 µL of Amies transport liquid medium after swab vortexing on SDA with chloramphenicol (Oxoid spa, Milan, Italy). The nail fragments were plated directly into SDA with chloramphenicol and in SDA supplemented with chloramphenicol and cycloheximide (Oxoid DTM spa, Milan, Italy). The culture plates were maintained at a temperature of 25°C for a period of at least three days and inspected daily and 30 days at 28°C in case of DTM for dermatophytes. Fungi isolated from nail samples were firstly identified by macroscopic features of colonies and morphological analyses by using lactophenol blue mount. The microscopic results were confirmed by proteomic analyses by applying the Matrix-assisted laser desorption ionization time-of-flight mass spectrometry (MALDI-TOF MS) (Bruker Daltonics, MS, USA) method. Captured spectra were analyzed using the MALDI Biotyper software package (version 3.4) containing the Filamentous Fungi Library 4.0 (Bruker Daltonics, Bremen, Germany). *Candida* species were identified by color, texture and microscopical morphology. The fungal growth was expressed as the number of colony-forming units per millilitre (CFU/mL). All samples with a colony count of >10 CFU/mL were considered positive for *Candida* spp. (20). For further differentiation, yeasts were grown on the brilliance™ Candida Agar (Oxoid Chromogenic Candida Agar), a selective differential medium used for the rapid identification (within 48 hr) of the clinically important *Candida* species. Green colonies are interpreted as *C. albicans*, blue colonies are defined as *C. tropicalis*, and light white to purple colonies are defined as *C. glabrata*; purple to pink colonies are defined as *C. krusei*; and pale colonies are referred to *C. parapsilosis*.

### 2.7 *Candida* genus identification by Oxford Nanopore’s MinION sequencing device

The nucleic acids of cultured samples were extracted utilizing Molgen Universal Extraction kit (Adaltis s.r.l. via Durini, 27, 20122 Milano, Italy) following the producer protocol*. Candida* species were further characterized by sequencing using a CE_IVD (Microbiome plus panel long kit _ 4bases, Manno, Switzerland) kit for the molecular identification of human microbiome communities, on the Nanopore platform. This kit contains all reagents necessary for the amplification and sequencing of both bacterial and fungal genes. Only the part relating to the fungal genes (18S, ITS1, 5.8S and ITS2) was used in our case. The data obtained (Q-fast) by sequencing were analyzed using a software analysis (One Codex database).

### 2.8 Cytokine detection

The serum levels of the cytokines IL-17A, IL-10 and TGF-β 1 were assessed at 0, 16, 24 and 52 weeks. Blood samples were collected from patients in serum-separating tubes and centrifuged for 15 minutes at 1000 rpm/min. Then, serum samples were subdivided into small aliquots and stored at −80°C. Cytokine levels were detected using a commercial Enzyme-linked Immunosorbent Assay (ELISA) (Tecan s.r.l., Milan, Italy), according to the manufacturer’s instructions. The detection limits (picogram per milliliter) of the assays were 1.6 for IL-17A, 0.390 for IL-10 and 22.000 for TGF-β 1. All tests were conducted using a pipetting robot and an automated ELISA analyzer (ThunderBolt^®^, Tecan s.r.l., Milan, Italy).

### 2.9 Statistical analyses

All data were initially entered into an Excel database (Microsoft, Version 2406, Redmond, Washington – United States) and the analysis was performed using IBM Corp.2017. IBM SPSS Statistics for Windows, vers.25.0. Armonk, NY: IBM Corp. The descriptive statistics consisted of the mean ± standard deviation (mean ± SD) for the parameters with normal distributions (after confirmation with histograms and Kolgomorov-Smirnov test), and median and range (min.; max.) for variables with non-normal distributions, while for the occurrences or frequencies the values ​​were expressed as a percentage (%). Error Bar graphs report data as means and 95% confidence interval (mean and 95%CI). Comparison among groups was performed with the Anova one-way, 2factor Anova or Anova for repeated measures, for normal variables or the Chi-Square test or Fisher’s exact test (if cells <5) for frequency variables. A *p*<0.05 was considered statistically significant (**Supplementary Material**).

## 3 Results

### 3.1 Demographic characteristics of enrolled patients

The clinical and demographic characteristics of the 70 patients (mean age 52.7 ± 14.8 years) recorded at baseline (T0) are shown in **Table 1**. In detail, 29 were females and 41 males, with 25 being smokers and 14 consumed alcohols more than 1 time a day. The mean age of disease onset was 27 years old. In the entire study population, there was a statistically significant decrease in weight during the 52 weeks of evaluation, ranging from an average value of 77.60 to 72.20 Kg, and it is completely comparable to the decrease in the average BMI in the population, from 26.02 ± 4.74 to 24.28 ± 4.75 Kg/m^2^. As expected between gender groups, males are statistically heavier (81.70 ± 13.80 Kg) and taller (176.60 ± 7.60 cm) (*p*<0.05, one-way ANOVA), while the study population resulted homogeneous for age and BMI. Patients were affected by moderate-to-severe plaque-type psoriasis with a mean PASI score of 12.17 (range 2–50), a mean DLQI score of 6.85 (range 0–30), a mean NAPSI scores of 28.02 (1-120), itch scores of 7.42 (2-10), PAIN VAS scores of 19.78 (0-100), scPGA moderate (3) 18.6% and severe (4) 2.9% and sPGA-G moderate 21.4% (3) and severe (4) 4.3%. Patients showed a body mass index (BMI) mean value of 26.02 (pre-obesity status), whereas 22 of 70 enrolled patients were overweight (BMI 26–29.99) and 6 were obese with a BMI> 30 Kg/m^2^ (range 30-39). Furthermore, eight patients (11.4%) had concomitant psoriatic arthritis (PsA). In addition, patient stratification was carried out for previous treatments: 15.7% (11/70) of subjects started therapy with apremilast as their first treatment; 71.43% (50/70) had previously received only topical treatment (corticosteroids) and 9 (12.9%) both topical and systemic treatments. Eleven patients had one or more systemic treatments, specifically 10% (7/70) cyclosporine, 4.3% (3/70) methotrexate and 10% (7/70) anti TNF-α. Clinical features and previous anti-psoriatic treatments are reported in **Table 1**. Cyclosporine administration was more used by male than female patients (4 *vs* 1 patients; *p*<0.05, Chi-Square test). Patient comorbidities were also recorded, with cardiovascular comorbidities (39%), mainly blood hypertension (35.7%), and followed by psoriatic arthritis (PsA) (11.4%), diabetes (10%), infections (11.4%), hypercholesterolemia (7.1%) and hypo-HDL-cholesterolemia (14.3%), inflammatory bowel diseases (1.5%), whereas 3.7% were affected by other pathologies. Twenty-three patients did not declare any comorbidities. Regarding the safety profile of the drug, during the 52 weeks of treatment, 21% of the patients presented diarrhea during the first two weeks of treatment with spontaneous resolution, 10% reported nausea and 9% insomnia, consistent with the known safety profile of apremilast. No serious AEs were reported. Only four of them discontinued the treatment primary due to inefficacy, while 6 were lost during follow-up. All the patients that did not complete 52 weeks of observation were excluded from the statistical analyses. Female patients dropped-out of the trial more than males (7 *vs* 2 patients; *p*<0.05; Chi-Square test). In the present study, 86% of patients reached treatment at W52.

**Table 1:** Baseline demographics and clinical characteristics of psoriatic patients.

Characteristic	Value
Age mean (SD), y	52.70 (± 14.80)
Men, n (%)	41 (59%)
BMI mean (SD), Kg/m^2^	26.02 (± 4.70)
Weight, mean (SD), Kg	77.60(± 14.40)
Smokers, n (%)	25 (36%)
Age at disease onset, mean (SD), y	27.00 (± 13.90)
Daily alcohols intake, n (%)	14 (20%)
Overweight (BMI 26–29.99 Kg/m^2^), n (%)	22 (31%)
Obese (BMI> 30 Kg/m^2^), n (%)	6 (9%)
Cardiovascular comorbidities, n (%)	27 (39%)
Blood hypertension being the most frequent, n (%)	25 (35.7%)
Hypo-HDL-cholesterolemia, n (%)	10 (14.3%)
Malignancy, n (%)	10 (14.3%)
Psoriatic arthritis (PsA), n (%)	8 (11.4%)
Chronic Infections, n (%)	8 (11.4%)
Diabetes, n (%)	7 (10%)
Hypercholesterolemia, n (%)	5 (7.15%)
Inflammatory bowel diseases, n (%)	1 (1.5%)
Bionaïve, n (%)	63 (90%)
Previous treatments, n (%)	59 (84.29%)
Previous only topical treatment	9 (12.86%)
Previous biological treatment, n (%)	7 (10%)
Multifailure, n (%)	2 (3%)

N, number; y, years.

### 3.2 Prevalence of *Candida* spp. colonization in psoriatic patients

In our population 33 out of 70 patients (47%) were colonized by *Candida* spp., detected in the oral cavity of 24 (34%) individuals and in stool samples of 9 (13%) individuals. The most common species was *C. albicans*, isolated in all 33 colonized patients. Notably, in 20 subjects only one species (*C. albicans*) was isolated, while in the remaining 13 a combination of two or more species (*C. albicans* plus non-*albicans* strains) was found in the oral cavity. With regard to non-*albicans* species, culture and molecular methods revealed that the most prevalent strains isolated from the oral cavity was *C. glabrata*, detected in 9/13 (69%) patients, followed by *C. parapsilosis*, detected in 4/13 (31%) patients. Other species, to a lesser extent, such as *C. krusei, C. africana* and *C. auris* were also found. All the data are deposited on BioProject NIH, number PRJNA1170201. A full list of the strains isolated from *Candida* co-infected patients is shown in **Table 2**. No significant *Candida* load (>10 CFU/mL) was found in DTT cutaneous lesions of psoriatic subjects. Moreover, the results indicated that *Candida* colonization was influenced by the gender as well as by comorbidities. In fact, the colonization rate in the oral cavity was higher in females than in males (19 *vs* 14 patients; *p*<0.05; one-way ANOVA) and, among females, the elderly were more infected than the younger ones (54.7 *vs* 45.8 years; *p*<0.05, 2-factors ANOVA). In addition, a high prevalence of *Candida* spp. was observed in patients with neoplasms (26 *vs* 19 patients; *p*<0.05; Chi-Square test), cardiovascular comorbidities and a high BMI (>25 Kg/m^2^) in comparison to those with no comorbidities (**Supplementary Material**). Interestingly, despite the high colonization rate of *Candida* spp. in our study population, the patients did not have any clinical signs or symptoms of oral candidiasis or even received antifungal treatments.

**Table 2:** Patients simultaneously colonized in the oral cavity by *C. albicans* and *non-albicans Candida* species.

Patients	*C. albicans*	*C. glabrata*	*C. krusei*	*C. parapsilosis*	*C. africana*	*C. auris*
Patient 1	*		*			
Patient 2	*			*		
Patient 3	*	*				
Patient 4	*	*		*		
Patient 5	*	*		*		
Patient 6	*	*				
Patient 7	*	*				
Patient 8	*				*	
Patient 9	*			*		
Patient 10	*	*				
Patient 11	*	*				
Patient 12	*	*				
Patient 13	*	*				*

### 3.3 Prevalence of onychomycosis in the study population

**Figure 1:**
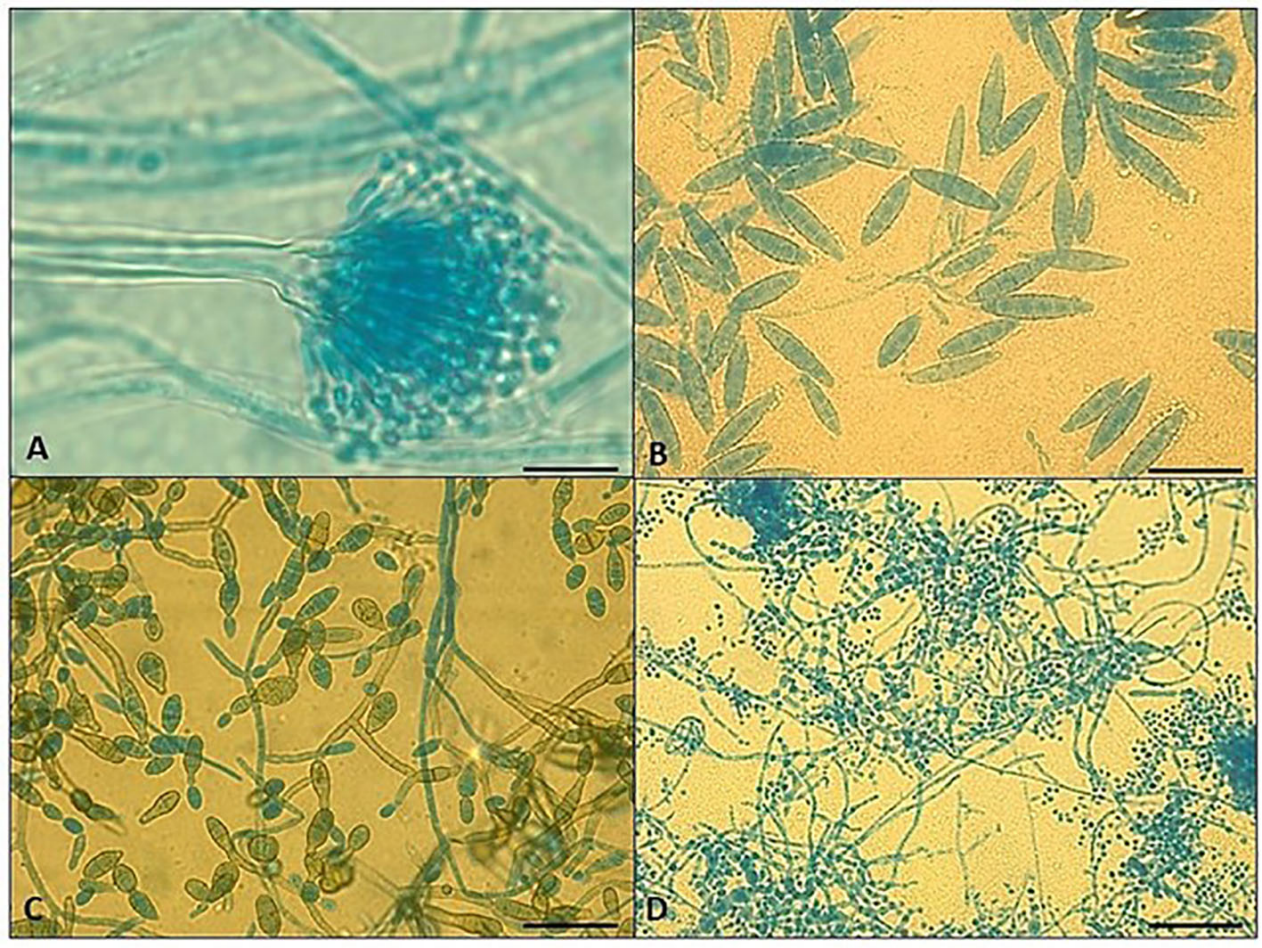
Microscopic examination of positive fungal cultures from nail clipping of psoriasis patients shows the presence of **(A)** *Aspergillus* spp. **(B)** *Microsporum canis* **(C)** *Alternaria alternata* and **(D)** *Trichophyton mentagrophytes* complex. Scale bar 50 µm.

In this study, we also evaluated the prevalence of onychomycosis in psoriatic patients. The results show that 27 (39%) patients were affected by onychomycosis. In detail, the prevalence of dermatophytes (*T. mentagrophytes complex*; *T. rubrum species complex*; *M. gypseum*) and non-dermatophytic mold (NDM; *Penicillium* spp; *Scopulariopsis brevicaulis*; *Aspergillus* spp., *Fusarium* spp. *Alternaria* spp.) isolated from nail clinical samples was similar (44.4%), followed by yeasts, including *Candida* spp. and *Trichosporon* spp., detected in 11.2% of the samples. In **Figure 1**, the microscopic images of various mold isolated from nail samples of psoriatic patients at baseline have been reported.

### 3.4 Effect of apremilast on the prevalence of *Candida* species and other fungal pathogens

**Figure 2:**
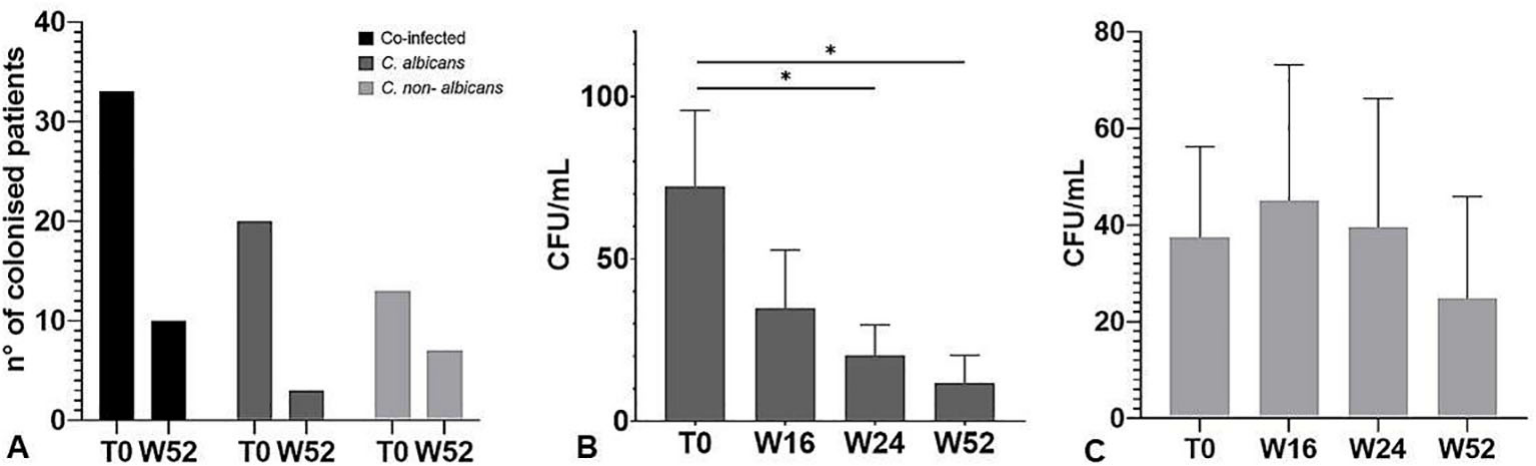
**(A)** Number of patients with positive culture for *Candida* species at baseline (T0) and 52 weeks of treatment. **(B)** CFU counts of *Candida albicans* and **(C)** non-*albicans* species at baseline (T0) and at all-time points considered. The graph shows the mean ± SD. *p<0.05 (One-way ANOVA).

To evaluate the impact of apremilast on the prevalence of *Candida* spp. in psoriasis patients, the CFU quantification from oral cavity swabs and stool samples was performed at baseline (T0), 16W, 24W and 52W. Interestingly, as reported in **Figure 2A**, the treatment with apremilast induced a full recovery from *Candida* colonization after 52 weeks in the majority of patients (83%) colonized with a single species of *Candida* (*C. albicans*); whereas in those co-infected by two or more *Candida* spp. apremilast failed to completely eliminate the fungus (colony counts >10 CFU/mL), although a significant reduction in *Candida* burden (40.52 *vs* 26.69 CFU/mL; one-way ANOVA; *p*<0.05) was observed in comparison to baseline. As shown in **Figures 2B, C**, apremilast exhibited higher effectiveness against *C. albicans* than non-*albicans Candida* species by inducing a reduction by 84% and 30% of *Candida* CFU, respectively, after 52 weeks of treatment.

**Figure 3:**
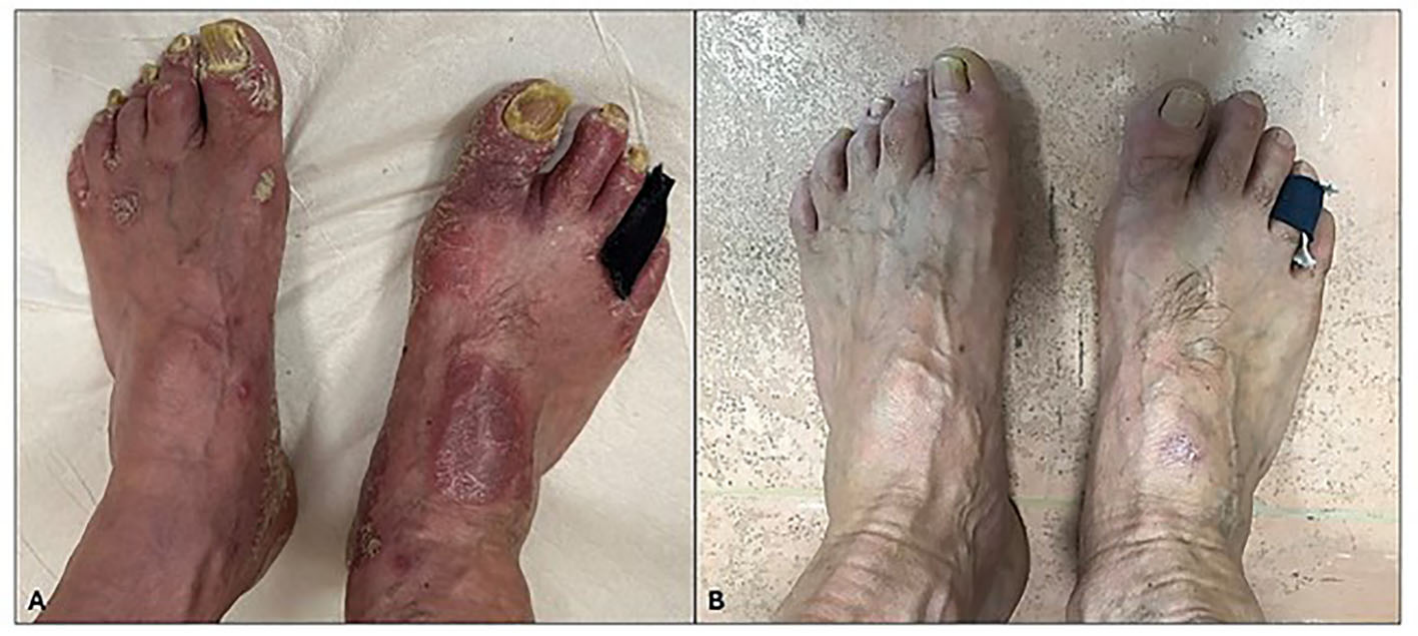
**(A)** Severe 10-nail and nail fold psoriasis before apremilast treatment. **(B)** Improvement in nail psoriasis after 52 weeks of treatment. Complete remission of psoriasis and concomitant onychomycosis.

Regarding onychomycosis, after 52 weeks of treatment, 78% (21/27) of the patients presented a complete clinical resolution of nail psoriasis and concomitant onychomycosis (**Figure 3**) with negative fungal cultures. The results are summarized in **Table 3**.

**Table 3:** Fungi identified from nail samples and mycological and clinical cure rates at the end of the study.

		Number of patients diagnosed with onychomycosis		Clinical cure (%)	Mycological cure
	Genus	T0	52W	52W	52W
Yeasts	*Candida* spp.	3	0	3/3 (100%)	3/3 (100%)
	*Trichosporon* spp.	1	0	1/1 (100%)	1/1 (100%)
Dermatophytes	*T. mentagrophytes* complex	4	0	4/4 (100%)	4/4 (100%)
	*T. rubrum species* complex	8	2	8/8 (100%)	6/8 (75%)
	*Microsporum gypseum*	3	0	3/3 (100%)	3/3 (100%)
NDM	*Penicillium* spp.	3	3	3/3 (100%)	0/3 (0%)
	*Scopulariopsis brevicaulis*	2	0	2/2 (100%)	2/2 (100%)
	*Aspergillus* spp.	2	0	2/2 (100%)	2/2 (100%)
	*Fusarium* spp.	3	1	3/3 (100%)	2/3(66%)
	*Alternaria* spp.	2	2	2/2 (100%)	2/2 (100%)

NDM, non-dermatophytic mold. *T., trichophyton*.

### 3.5 Evaluation of PASI, NAPSI, DLQI, ITCH VAS, scPGA and sPGA-G indexes

**Figure 4:**
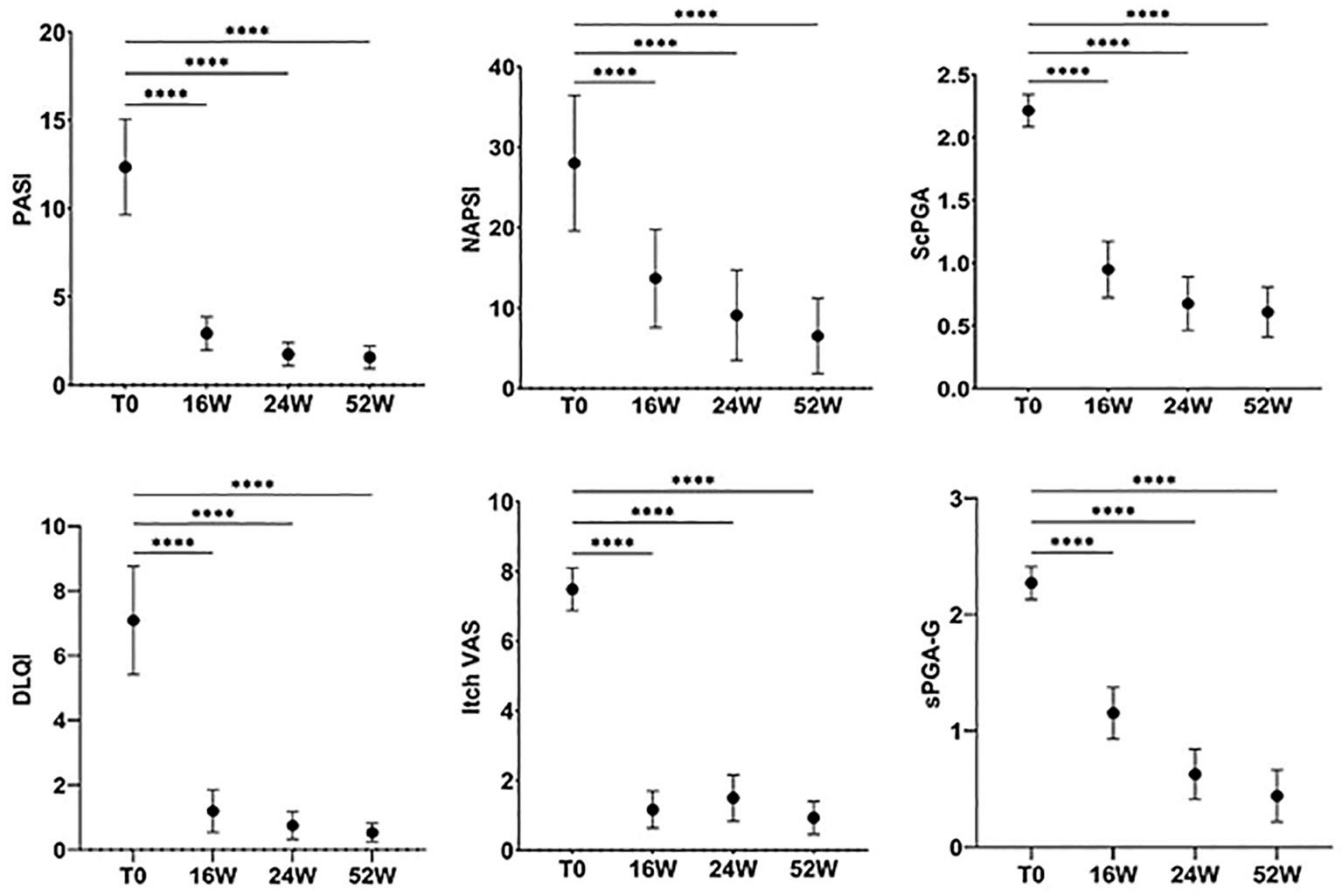
PASI, NAPSI, DLQI, ITCH VAS, ScPGA and sPGA-G scores variation during treatment. The graph shows the mean ± 95% CI. ****p<0.001 (Repeated measures ANOVA).

**Figure 5:**
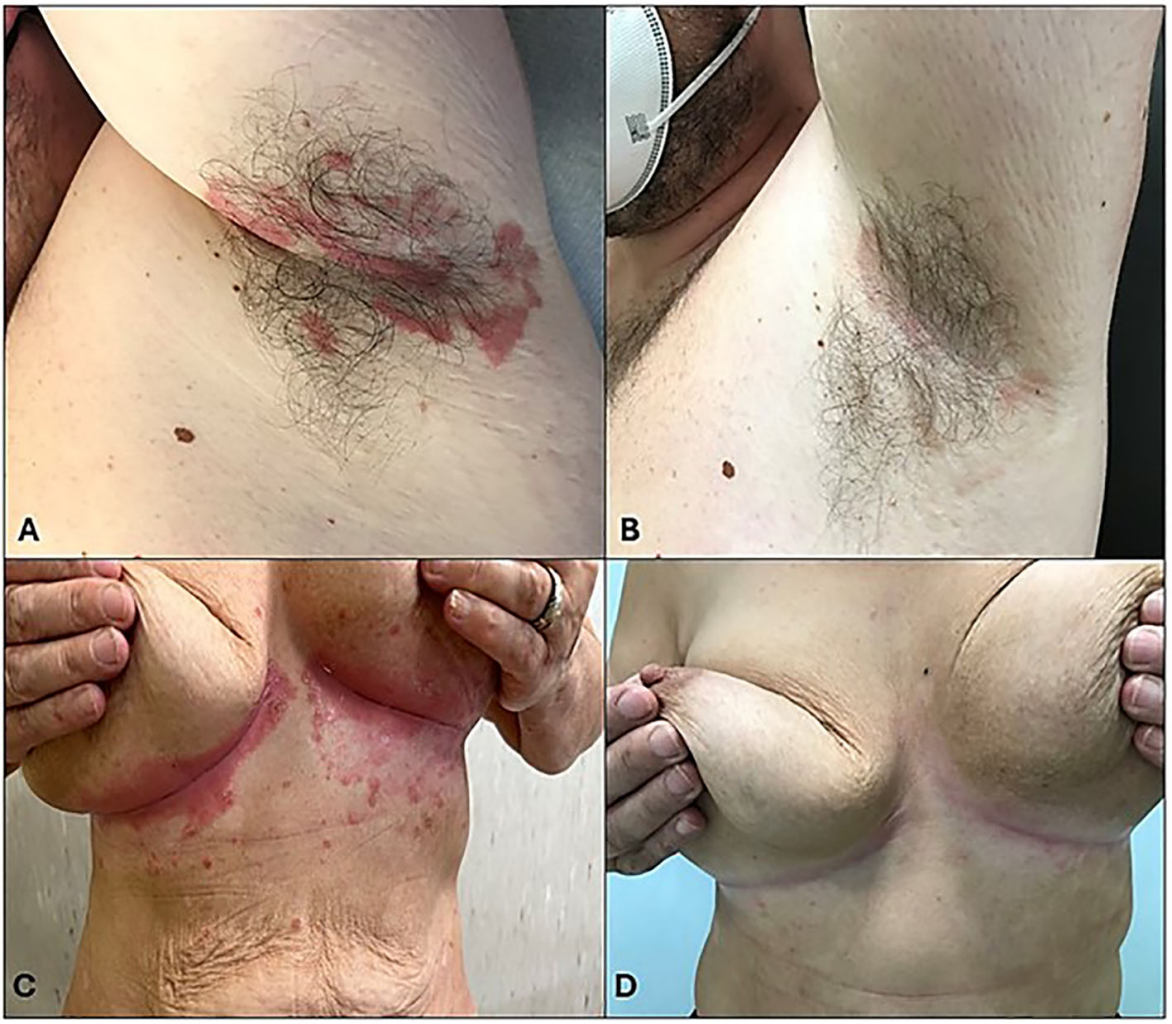
Clinical evaluation at T0 and after 52 weeks of treatment. **(A–C)** Clinical examination revealed inverse psoriasis in the context of metabolic syndrome, **(B–D)** resolved after 52 weeks of treatment.

To investigate the efficacy of apremilast in DTT psoriasis, we evaluated PASI, NAPSI, DLQI, itch VAS, PAIN VAS, scPGA and sPGA-G scores at baseline and at different time points (week 16, 24, 52). Statistical analysis showed a significant reduction of PASI, from 12.17 to 1.57 after 52 weeks of treatment (*P*<0.0001; Repeated measures ANOVA). The mean NAPSI score decreased from 28,02 at T0 to 6.53 at week 52 (*p*<0.0001; Repeated measures ANOVA). In addition, to the improvement in terms of QoL, we also found a statistically significant reduction after 52 weeks of treatment (*p*<0.0001; Repeated measures ANOVA), from 6.85 to 0.58 (Repeated measures ANOVA test; *p*<0.05), itch VAS from 7.42 to 0.93 (*p*<0.0001; Repeated measures ANOVA test) and pain VAS from 19.78 to 3.97 (*p*<0.0001; Repeated measures ANOVA test) (**Figures 4**, **5**). The decrease of the above-mentioned parameters was statistically significant from the 4^th^ month of treatment. In this study we also examined a possible correlation between the prevalence of isolated *Candida* spp. and severity of psoriasis. For this purpose, we stratified the patient population into two groups: *Candida* colonized (>10 CFU/mL) and *Candida* non-colonized patients at baseline and at week 52. Despite slightly higher baseline values for patients colonized by *Candida* spp., no statistically significant differences were found in PASI, NAPSI, ITCH, PAIN, ScPGA and PGA-G scores between the two subgroups in line with previous works (22,23), except in the 4 week, where patients with comorbidities and colonized by *Candida* showed a slow reduction in mean PASI score, compared to patients without comorbidities (*p*<0.05, one-way ANOVA), suggesting a delayed drug response in patients colonized by *Candida* spp.

### 3.6 Effect of apremilast on biochemical blood values

**Figure 6:**
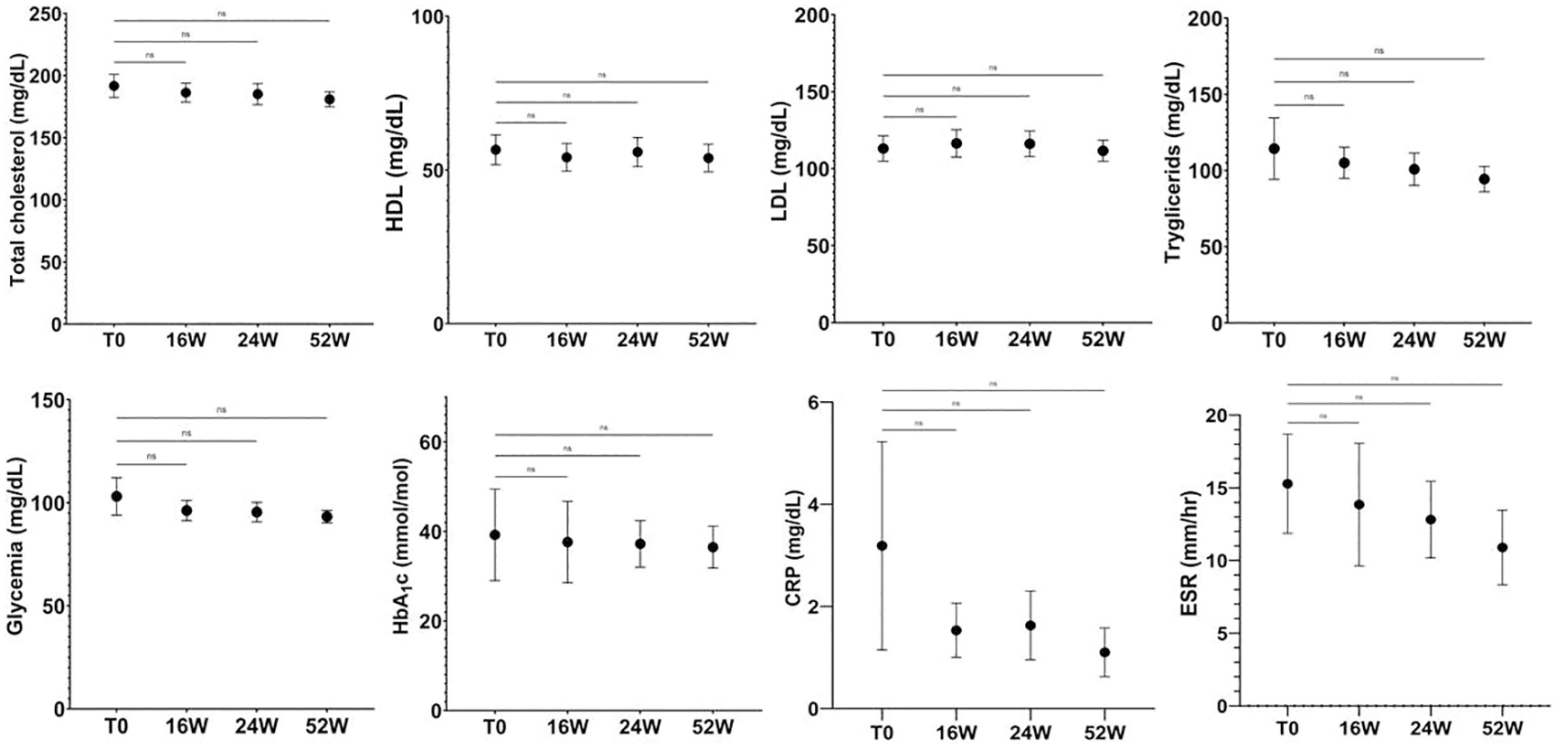
Plasma glucose level value over study period. At week 52, glucose levels decreased from 103.10 to 93.30 mg/dL. In parallel, total cholesterol decreased, from 191.70 to 180.90 mg/dL without diet modification in the population. Ns, not significant; repeated measures ANOVA. Error bars represent 95% CI. CI, Confidence interval.

Psoriasis patients are at increased risk of developing metabolic diseases. Proinflammatory cytokines such as IL-17, IL-6, IL-1 and TNF-α are increased in psoriasis and numerous studies suggest that they play a pivotal role in developing type 2 diabetes, hypertension, dyslipidaemia, obesity, insulin resistance and their complications (40). In this study the impact of apremilast on metabolic parameter was also evaluated. As shown in **Figure 6**, in our patients the drug significantly reduced the serum levels of glucose and total cholesterol after 52 weeks of treatment. Further, a significant decrease in circulating levels of C reactive Protein (CRP), triglycerides and LDL cholesterol was also induced by apremilast in comparison to baseline. The plasmatic glucose level showed a decreasing trend in the first 4 months of therapy that stabilized in the range between 91-93 mg/L at week 52, in comparison to the mean value of 99.32 mg/L at baseline. Interestingly, with regard to the patients with hypo-HLD cholesterolemia, a slight increase in HDL from baseline to week 52, ranging from 29.70 to 37.50 mg/dL was observed. In light of these evidences our results underline the beneficial role of apremilast in psoriasis patients with dysregulation of lipid pathway.

### 3.7 Effect of apremilast on serum cytokine levels

**Figure 7:**
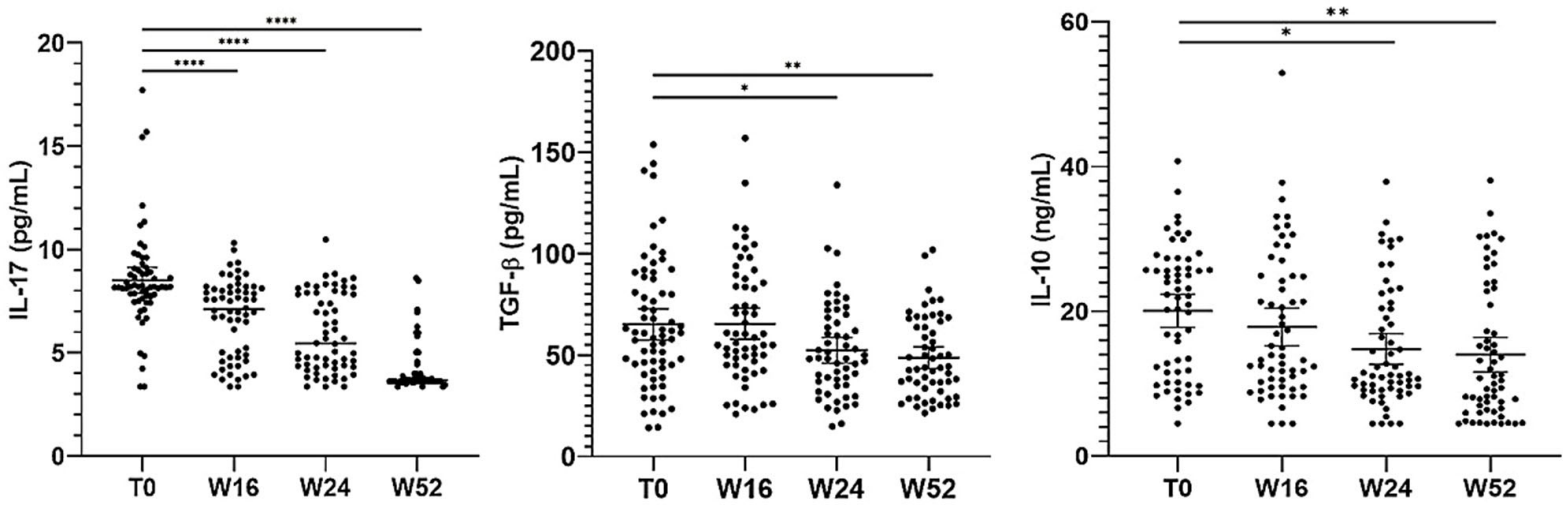
Serum cytokine levels at different time points of the study. The graph shows the mean ± 95% CI. *p<0.05, **p<0.01, ****p<0.0001 (one-way ANOVA).

Herein, we evaluated the impact of apremilast on circulating levels of IL-17A, TGF-β 1 and IL-10 at baseline and after 16, 24 and 52 weeks of apremilast treatment. Our results (**Figure 7**) revealed a significant decrease in serum IL-17A, TGF-β 1 and IL-10 levels up 52 weeks of apremilast administration compared with baseline (from 8.51 to 4.16 pg/mL; from 66.10 to 48.70 ng/mL and from 20.05 to 14 pg/mL, respectively). Moreover, no statistically significant differences in serum cytokine concentrations were observed between *Candida* colonized and non-colonized patients, as well as between patients colonized by a single *Candida* species (*C. albicans*) and those co-infected by more than one *Candida* species. In addition, no correlation was found between the serum cytokines and gender in both *Candida* spp. colonized and non-colonized groups.

## 4 Discussion

Psoriasis is a chronic inflammatory disease caused by a complex interaction between genetic and immunologic factors in a predisposing environment. Although it was considered primarily a disease of keratinocyte dysfunction, dysregulation of the immune system, potentially correlated with gut and/or skin dysbiosis among others, is now recognized as a key event in the pathogenesis of this disease (43). Restoring the gut microbiome is considered nowadays a promising preventive and therapeutic option for psoriasis. In this regard, several reports suggest that high levels of *Candida* spp. colonization in the gut and/or skin may play a crucial role in initiating, exacerbating, and maintaining chronic inflammation in psoriasis (44–46). Waldman et al. (15) detected a high *Candida* load in saliva and stool samples of psoriatic patients, although no correlation was found between *Candida* load and PsO severity. In another study conducted by Lesan et al. (25) on 70 psoriatic patients, *Candida* was isolated from the oral cavity of 20% subjects *versus* 2.8% of healthy controls, but none of them had clinical features of oral candidiasis. Moreover, Picciani et al. (26) found that 26% of psoriatic patients were positive for oral candidiasis, in comparison to 0 of 140 healthy controls, and candidiasis was more common in patients with severe psoriasis than in those with mild or moderate disease. Based on these evidences, we investigated the prevalence of *Candida* spp. colonization in psoriatic patient with DTT PsO. In line with the previous studies, our results confirm a high *Candida* spp. colonization rate in the oral cavity and gut of psoriatic patients. In fact, *Candida* was detected in 33 (47%) out of 70 patients, with *C. albicans* being the most frequent species. Notably, 20 individuals were positive for only one *Candida* species (*C. albicans*), while the remaining 13 were positive for two or more *Candida* spp. In the latter, *C. glabrata* was the most common non-*albicans* species associated with *C. albicans.* Other authors have shown that *C. albicans* and *C. glabrata* are frequently co-isolated in oropharyngeal candidiasis. *C. albicans* adhesins, and in particular agglutinin-like sequence (Asl) 3, in fact, contribute not only to the ability of the fungus to adhere and colonize host tissues, but also serve as binding moieties for bacteria i.e., *Streptococcus gordonii*, *Pseudomonas aeruginosa* and *Staphylococcus aureus*, as well as other non-hyphal forming *Candida* species such as *C. glabrata* (47). Moreover, our results reveal that *Candida* colonization was strongly influenced by the gender and comorbidities, resulting to be higher in female than in male patients and a high colonization rate was also observed in subjects with neoplasms, cardiovascular comorbidities and overweight. However, despite the high prevalence of *Candida* among patients with psoriasis, according to other authors (15, 25), we did not observe any correlation between *Candida* colonization and clinical severity of psoriasis (PASI, NAPSI, scPGA, sPGA-G). To our best knowledge, this is the first clinical study demonstrating a high prevalence of oral/gut *Candida* spp. in DTT psoriatic patients. Notably, this clinical phenotype of PsO could be initially responsive to systemic first line drugs (cyclosporine, methotrexate, retinoids) although during treatment relapses may occur. Moreover, biological treatments, such as IL-17 inhibitors, have shown to be more effective than traditional drugs, favoring a rapid healing of psoriasis but in some patients, such treatments could increase the risk of mycotic infections. In addition to *Candida*, other fungi may play a role in psoriasis pathogenesis. In our study population, 27 (39%) patients presented a concomitant onychomycosis caused by dermatophytes, NDM and yeasts. Interestingly, after 52 weeks of treatment, 78% of the patients presented a complete clinical resolution of nail psoriasis and onychomycosis and in 70% mycological culture tests were negative. It should be noted that in our patients all the concomitant onychomycoses have been identified before apremilast treatment as clinical routine practice and no new nail infections were observe in the study period. Nail psoriasis and onychomycosis are the two most frequent diseases affecting the nail unit (48) and their coexistence could not be incidental. Ghosal et al. (49) found that nail onychomycosis was more frequent in psoriatic patients with skin Koebner phenomenon respect to patients without traumatism in anamnesis. These observations support the hypothesis that Koebner phenomenon induced by the fungal invasion may worsen nail psoriasis. Although over the years significant advances have been made in understanding the pathogenic mechanisms of psoriasis, leading to the development of various therapeutic options, the treatment of this disease remains challenging in clinical practice. A major challenge in addressing DTT, mainly in patients without significant body surface involvement (28), is a tailored treatment for this clinical psoriasis phenotype (50). Recently, IL-17 inhibitor drugs represent the most effective therapeutic option for patients with moderate/severe plaque psoriasis, also in those with DTT involvement (35–37). However, randomized clinical trials (RCT) and real-life studies demonstrated an increase in fungal and bacterial infections in patients treated with IL-23 or IL-17 inhibitors (38, 49, 51). In 2023, in a double-blinded placebo-controlled trial apremilast demonstrated the clinical efficacy in the treatment of psoriasis affecting genital areas (41) as well as other specific sites, such as the scalp, nails, and palmoplantar areas (42). In line with these clinical studies, here we demonstrated the therapeutic efficacy and safety of apremilast in the treatment of DTT psoriatic patients, achieving improvement in itching and relative QoL, in both *Candida* colonized and *Candida* non-colonized subjects. In terms of clinical efficacy, no significant differences were found in our population between the two groups of psoriatic patients. The beneficial effects of apremilast on clinimetric scores correlated in our study population with a significant decrease in serum levels of IL-17A, and TGF-β 1, which have proven to be higher in individuals with psoriasis in comparison to healthy controls (52, 53), although no statistical correlation has been found between IL-17 concentrations and PASI/NAPSI scores (54, 55). A significant reduction in IL-10 serum levels was also observed in our patients after apremilast treatment. This result seems to be in contrast with the immunoregulatory properties of IL-10. However, it should be pointed out that the role of this cytokine in psoriasis remains unclear. Immunohistochemical investigations suggested a low IL-10 protein expression in psoriatic lesional skin as compared to healthy skin (56). On the other hand, no significant differences were reported in the literature in the baseline serum levels between patients with psoriasis and healthy individuals (57–60). Moreover, in a previous report Wakiya et al. (61) investigated the therapeutic efficacy of apremilast and its impact on serum cytokine levels in Behçet’s disease patients. The authors found no significant changes in IL-10 concentrations after three months of apremilast initiation. Conversely, previous *in vitro* studies have demonstrated that apremilast was able to increase IL-10 production by B cells (62). Thus, conflicting data are reported in the literature on the impact of apremilast on IL-10 serum levels. Further studies are needed on a large cohort of psoriatic patients to better clarify this aspect. The ability of apremilast to modulate the serum levels of the inflammatory cytokine IL-17 could also explain, at least in part, the negative impact of such drug on *Candida* colonization in our psoriatic population. In fact, in this study we demonstrated for the first time the effectiveness of apremilast in inhibiting the fungal growth in the majority of patients (83%) colonized by only one *Candida* species. It is assumed that the intestinal barrier dysfunction and the consequent increase of intestinal permeability that occurs in gut dysbiosis may cause microbial translocation, leading to systemic, low-grade inflammation. These effects may create a “vicious cycle” in which low-level inflammation may promote fungal colonization, which in turn, can promote further inflammation (63, 64). In this scenario it is likely that apremilast, due to its own anti-inflammatory properties, could restore gut homeostasis in psoriatic patients, thus counteracting *Candida* overgrowth. In the inflamed tissues, overexpression of PDE4 isoforms and defective cAMP-mediating pathway were identified for the first time in chronic ulcerative colitis (UC) patients. Therapeutic inhibition of PDE4 by apremilast ameliorated the clinical symptoms in patients with chronic UC, as evidenced by improvements on mucosal ulcerations, tissue fibrosis, and inflammatory infiltrations (65). Consistent with these findings, in a murine model of chronic UC apremilast was proven to play a role in the maintenance of intestinal barrier integrity, reestablishing the mucosal immune homeostasis, by interfering with the crosstalk between human epithelial barrier and immune cells. In addition, by modulating the gut microbiota composition this drug might exert regulatory effects on antimicrobial responses (66) Comorbidities like diabetes, obesity, dyslipidaemia, metabolic syndrome, and non-alcoholic fatty liver disease, have been consistently found to be associated with chronic plaque psoriasis (67). Notably, metabolic syndrome is a condition of low-grade chronic inflammation where gut dysbiosis plays a crucial role. Besides intestinal bacteria, opportunistic fungi such as *C. albicans*, *Aspergillus* spp. and *Meyerozyma* spp. may also contribute to the onset of metabolic diseases by activating the immune system and/or producing harmful metabolites (68). Yeter et al. (69) found that oral *Candida* colonization represented a risk factor for chronic inflammation, atherosclerosis and coronary artery disease in hemodialysis patients. Consistently, in our study a high *Candida* colonization rate was found in patients with cardiometabolic comorbidities and overweighed. A beneficial effect of apremilast, *via* inhibiting PDE4, has been previously associated with weight loss and other cardiometabolic benefits in psoriatic patients (70, 71), including reduced glycated hemoglobin A1c (HbA1c) and serum glucose levels, thus suggesting potential novel therapeutic uses of PDE4 inhibitors (72–76). Interestingly, in line with the literature data, our results demonstrated that apremilast affected the body weight, BMI and metabolic profile in our population, favoring a reduction in serum levels of total cholesterol, triglycerides and blood glucose. The mechanism behind the potential role of apremilast on metabolic and cardiovascular benefits is still not fully understood. However, Ikonomidis et al. (77) demonstrated that apremilast confers a greater improvement of endothelial glycocalyx integrity, microvascular perfusion, arterial elasticity and left ventricular myocardial function compared with etanercept or cyclosporine treatment, suggesting a favorable profile of PDE4 inhibition on cardiovascular function (74–79). In addition, Wang et al. (78) also demonstrated a significant efficacy of apremilast on ischemic stroke outcomes (79, 80). Thus, those findings could pave the way for the potential use of PDE4 inhibitors in the treatment of cardiometabolic comorbidities and oxidative stress in the future. Despite the promising results, this study presents some limitations. First of all, it is a real-life study with a high percentage of patients who dropped out (14%) and did not complete the planned treatment. Moreover, a longer follow-up time to evaluate drug survival and maintenance of clinical response should be performed. It should be also pointed out that, although the sample collection procedures have been standardized in all patients, oral and cutaneous swabs collection are operator-dependent, thus representing a limitation of the study. Finally, we evaluated the levels of IL-10, IL-17 and TGF-β 1 only in the serum. Nevertheless, the cytokine analysis in the psoriatic skin lesions based on the expression quantification of cytokine mRNA or immunohistochemistry analyses would be more useful to explain the contribution of inflammatory/regulatory cytokines in the immunopathogenesis of this disease.

## 5 Conclusion

Overall, our results suggest that although psoriasis is considered a multifactorial disease, *Candida* may play a possible role as a trigger factor in the pathogenic process of this inflammatory disease. In this context, apremilast has been shown to ameliorate psoriasis symptoms and counteract *Candida* overgrowth. We hypothesized that apremilast, by dampening inflammation, due its ability to modulate the serum levels of the inflammatory cytokine IL-17, might arrest the “vicious cycle”, triggered by *Candida* itself, thus restoring the gut homeostasis in psoriatic patients. Thus, considering the crucial role of IL-17 in protecting against *Candida* infections, apremilast by modulating this cytokine does not increase the risk of fungal infections, exerting a protective effect against mucosal candidiasis. Given the high rate of *Candida* colonization in psoriatic patients and the potential role of *Candida* in psoriasis onset, the use of PDE-4 inhibitors may represent an effective therapeutic approach for better management of sub-clinical risk of candidiasis in those patients. Moreover, apremilast may offer a unique opportunity to control systemic inflammation by improving the metabolic profile in psoriatic subjects. Finally, this study confirms the safety profile and therapeutic efficacy of apremilast in treating DTT psoriasis, regardless of age, sex, disease onset, BMI, cardiovascular comorbidities and neoplastic conditions. Despite the promising results, further studies are needed to better understand the relationship between gut homeostasis, obesity and psoriasis and the pleiotropic role of PDE4 inhibitors in the management of psoriasis as a complex disease.

## Data Availability

The datasets presented in this study can be found in online repositories. The names of the repository/repositories and accession number(s) can be found in the article/**Supplementary Material**.
