# Supplementary material for: Prevalence of fungal colonization among patients with psoriasis in difficult-to-treat areas: impact of apremilast on mycotic burden and clinical outcomes
Source: Front Immunol. 2024 Dec 10;15:1508489. doi: 10.3389/fimmu.2024.1508489 (PMC11666449; doi:10.3389/fimmu.2024.1508489)
Supplement: Supplementary file 1 [file DataSheet1.pdf]

**University of**
Laboratory of Biometry. Department of Systems Medicine, 1 Montpellier Street - 00133 Rome, Italy.
Tel. +390672596416; E-mail: sorge@uniroma2.it

**Ricercatore:** U.O.S.D Dermatology; terenziocosio@gmail.com; Tel. +0039 3351764572; +0039 3665987179

# REPORT Amgen3

*Studio sull'efficacia, sicurezza e tollerabilità della somministrazione di Apremilast per il trattamento della psoriasi delle sedi difficili. Studio retrospettivo in aperto.*

## LEGENDA:

**Variabili di valutazione di efficacia del trattamento di APREMILAST nel tempo**
VES= velocità di eritrosedimentazione
**PCR= proteina c reattiva**
PASI= indice gravità psoriasi
PAIN VAS= dolore riferito psoriasi
TJC= tender join count( articolazioni tumefatte psoriasi)
DLQI= questionario qualità vita con psoriasi (< 10 lieve)
NAPSI= Valori psoriasi ungueale
**Variabili per le precedenti terapie:**
HbA1= emoglobina glicata
CYA= CICLOSPORINA
MTX= METOTREXATO
RTX= RITUXIMAB
ADA= ADALIMUMAB
ETA= ETANERCEPT
GOL= GOLIMUMAB
IFX= INFLIXIMAB
UST= USTEKINUMAB
CZP= CERTOLIZUMAB
SZP= SALAZOSULFAPIRIDINA
**Codifiche:**
Variabile tempo; T0,T4,T6,T12
Factor1; valori delle variabili ai 4 tempi
Sex; M=1;F=0
LAVORO; pensionato=disoccupato=0; operaio=artigiano=Turnistaali=commessa=1; impiegato,assicuratore;insegnante;maestra=2; medico;libero prof. =3
DROPOUT;1=terapia sospesa; 0=terapia continua

INFEZ1S; 1S;0N
TIPOINF1S; 1=albicans;2=albicans+noalbicans;3=noalbicans
CURAINF1S; 1S;0N

## NUOVA ELABORAZIONE DA PAG.19

## ANALISI STATISTICA

All data were initially entered into an Excel database (Microsoft, Redmond, Washington – United States) and the analysis was performed using IBM Corp.2017. IBM SPSS Statistics for Windows, vers.25.0. Armonk, NY: IBM Corp.
Descriptive statistics consisted of the mean ± standard deviation for parameter with normal distributions (after confirmation with histograms and the Kolgomorov-Smirnov test), median and range (min.;Max.) for variables with non-normal distributions and percentage (%) for occorrencies or frequencies.
Comparison among groups was performed with the ANOVA one-way o ANOVA for repeated measured for normal variables or the Chi-Square test or Fisher's exact test (if cells<5) for frequencies variables.
A p value of < 0.05 was considered statistically significant.

## COMPOSIZIONE CAMPIONE DI PARTENZA T0 (N°=70; 29F;41M)

| Descrizione | | | | | | | Anova oneway | | | | | |
|---|---|---|---|---|---|---|---|---|---|---|---|---|
| | | | | | | | | | | | | |
| | sexM1 | N | Mean | SD | Min. | Max. | | Sum of sq | df | $Mean^2$ | F | Sig. |
| AGE | F=0 | 29 | 51,3 | 13,9 | 25 | 79 | Between | 97,591 | 1 | 97,591 | ,441 | ,509 |
| | M=1 | 41 | 53,7 | 15,5 | 25 | 83 | Within | 15044,695 | 68 | 221,246 | | |
| | total | 70 | 52,7 | 14,8 | 25 | 83 | Total | 15142,286 | 69 | | | |
| PESO | F=0 | 26 | 69,9 | 12,3 | 50 | 114 | Between | 2223,903 | 1 | 2223,903 | 12,715 | ,001 |
| | M=1 | 41 | 81,7 | 13,8 | 56 | 130 | Within | 11369,142 | 65 | 174,910 | | |
| | total | 67 | 77,1 | 14,4 | 50 | 130 | Total | 13593,045 | 66 | | | |
| ALTEZZA | F=0 | 29 | 166,6 | 7,3 | 150 | 181 | Between | 1703,146 | 1 | 1703,146 | 30,717 | ,000 |
| | M=1 | 41 | 176,6 | 7,6 | 160 | 195 | Within | 3770,340 | 68 | 55,446 | | |
| | total | 70 | 172,5 | 8,9 | 150 | 195 | Total | 5473,486 | 69 | | | |
| BMI | F=0 | 26 | 25,42 | 5,64 | 19,29 | 46,25 | Between | 8,922 | 1 | 8,922 | ,413 | ,523 |
| | M=1 | 41 | 26,17 | 3,91 | 19,61 | 37,18 | Within | 1404,406 | 65 | 21,606 | | |
| | total | 67 | 25,88 | 4,63 | 19,29 | 46,25 | Total | 1413,328 | 66 | | | |

**Commento:** Come atteso tra gruppi di genere i maschi sono statisticamente più pesanti e più alti, tuttavia l'età e il bmi risultano del tutto omogenei.

### Tempistica e drop-out

| Time | T0 | T4 | T6 | T12 |
|---|---|---|---|---|
| **N°totali** | **70** | **70** | **70** | **70** |
| **Drop-out** | **10** | **10** | **10** | **10** |
| **Perdita %** | **14,3** | **14,3** | **14,3** | **14,3** |
| **N°effettivi** | **60** | **60** | **60** | **60** |

## COMPOSIZIONE CAMPIONE EFFETTIVO A T12 mesi (N°60; 21F; 39M)

| Descrizione | | | | | | | Anova oneway | | | | | |
|---|---|---|---|---|---|---|---|---|---|---|---|---|
| | sexM1 | N | Mean | SD | Min. | Max. | | Sum of sq | df | $Mean^2$ | F | Sig. |
| AGE | F=0 | 21 | 52,1 | 15,0 | 25 | 79 | Between | 86,441 | 1 | 86,441 | ,370 | ,545 |
| | M=1 | 39 | 54,6 | 15,4 | 25 | 83 | Within | 13534,542 | 58 | 233,354 | | |
| | total | 60 | 53,7 | 15,2 | 25 | 83 | Total | 13620,983 | 59 | | | |
| PESO | F=0 | 21 | 70,7 | 12,8 | 50 | 114 | Between | 1539,231 | 1 | 1539,231 | 8,341 | ,005 |
| | M=1 | 39 | 81,3 | 14,0 | 56 | 130 | Within | 10702,952 | 58 | 184,534 | | |
| | total | 60 | 77,6 | 14,4 | 50 | 130 | Total | 12242,183 | 59 | | | |
| ALTEZZA | F=0 | 21 | 167,1 | 7,4 | 150 | 181 | Between | 1081,484 | 1 | 1081,484 | 20,931 | ,000 |
| | M=1 | 39 | 176,0 | 7,1 | 160 | 195 | Within | 2996,850 | 58 | 51,670 | | |
| | total | 60 | 172,8 | 8,3 | 150 | 195 | Total | 4078,333 | 59 | | | |
| BMI | F=0 | 21 | 25,60 | 6,00 | 16,71 | 46,25 | Between | 5,939 | 1 | 5,939 | ,261 | ,611 |
| | M=1 | 39 | 26,25 | 3,97 | 17,90 | 37,18 | Within | 1318,962 | 58 | 22,741 | | |
| | total | 60 | 26,02 | 4,74 | 16,71 | 46,25 | Total | 1324,900 | 59 | | | |

**Commento:** Come atteso tra gruppi di genere i maschi sono statisticamente più pesanti e più alti, tuttavia l'età e il bmi risultano del tutto omogenei.

**SEX1M * FUMO Crosstabulation**

| | | FUMO | | | Chi-SquareTests | | | |
|---|---|---|---|---|---|---|---|---|
| sex | | 0=N | 1=S | Total | | Value | df | Sig. |
| 0=F | Count<br>%sex<br>%FUMO | 15<br>71,4%<br>42,9% | 6<br>28,6%<br>24,0% | 21<br>100,0%<br>35,0% | Chi-Square<br>N | 2,279<br>60 | 1 | ,131 |
| 1=M | Count<br>%sex<br>%FUMO | 20<br>51,3%<br>57,1% | 19<br>48,7%<br>76,0% | 39<br>100,0%<br>65,0% | Commento: il test certifica che le occorrenze di FUMO risultano proporzionate tra gruppi di genere. | | | |
| Total | Count<br>%sex<br>%FUMO | 35<br>58,3%<br>100,0% | 25<br>41,7%<br>100,0% | 60<br>100,0%<br>100,0% | | | | |

**SEX1M * ALCOL Crosstabulation**

| | | **ALCOL** | | | Chi-SquareTests | | | |
|---|---|---|---|---|---|---|---|---|
| sex | | 0=N | 1=S | Total | | Value | df | Sig. |
| 0=F | Count | 17 | 4 | 21 | Chi-Square | ,332 | 1 | ,565 |
| | %sex | 81,0% | 19,0% | 100,0% | N | 60 | | |
| | % ALCOL | 37,0% | 28,6% | 35,0% | | | | |
| 1=M | Count | 29 | 10 | 39 | Commento: il test certifica che le occorrenze di ALCOL risultano proporzionate tra gruppi di genere. | | | |
| | %sex | 74,4% | 25,6% | 100,0% | | | | |
| | % ALCOL | 63,0% | 71,4% | 65,0% | | | | |
| Total | Count | 46 | 14 | 60 | | | | |
| | %sex | 76,7% | 23,3% | 100,0% | | | | |
| | % ALCOL | 100,0% | 100,0% | 100,0% | | | | |

**SEX1M * FAMILIARITA' Crosstabulation**

| | | **FAMILIARITA'** | | | Chi-SquareTests | | | |
|---|---|---|---|---|---|---|---|---|
| sex | | 0=N | 1=S | Total | | Value | df | Sig. |
| 0=F | Count | 12 | 9 | 21 | Chi-Square | ,659 | 1 | ,417 |
| | %sex | 57,1% | 42,9% | 100,0% | N | 60 | | |
| | % FAMILIARITA' | 40,0% | 30,0% | 35,0% | | | | |
| 1=M | Count | 18 | 21 | 39 | Commento: il test certifica che le occorrenze di FAMILIARITA' risultano proporzionate tra gruppi di genere. | | | |
| | %sex | 46,2% | 53,8% | 100,0% | | | | |
| | % FAMILIARITA' | 60,0% | 70,0% | 65,0% | | | | |
| Total | Count | 30 | 30 | 60 | | | | |
| | %sex | 50,0% | 50,0% | 100,0% | | | | |
| | % FAMILIARITA' | 100,0% | 100,0% | 100,0% | | | | |

**SEX1M * COMORBIDITA' Crosstabulation**

| | | COMORBIDITA' | | | Chi-SquareTests | | | |
|---|---|---|---|---|---|---|---|---|
| sex | | 0=N | 1=S | Total | | Value | df | Sig. |
| 0=F | Count | 9 | 12 | 21 | Chi-Square | ,877 | 1 | ,349 |
| | %sex | 42,9% | 57,1% | 100,0% | N | 60 | | |
| | %COMORBIDITA' | 42,9% | 30,8% | 35,0% | | | | |
| 1=M | Count | 12 | 27 | 39 | Commento: il test certifica che le occorrenze di COMORBIDITA' risultano proporzionate tra gruppi di genere. | | | |
| | %sex | 30,8% | 69,2% | 100,0% | | | | |
| | %COMORBIDITA' | 57,1% | 69,2% | 65,0% | | | | |
| Total | Count | 21 | 39 | 60 | | | | |
| | %sex | 35,0% | 65,0% | 100,0% | | | | |
| | %COMORBIDITA' | 100,0% | 100,0% | 100,0% | | | | |

**SEX1M * K COLON adenoma tubulo-villoso Crosstabulation**

| | | K COLON | | | Chi-SquareTests | | | |
|---|---|---|---|---|---|---|---|---|
| sex | | 0=N | 1=S | Total | | Value | df | Sig. |
| 0=F | Count | 21 | 0 | 21 | Chi-Square | ,548 | 1 | ,459 |
| | %sex | 100,0% | ,0% | 100,0% | N | 60 | | |
| | % K COLON | 35,6% | ,0% | 35,0% | | | | |
| 1=M | Count | 38 | 1 | 39 | Commento: il test certifica che le occorrenze di K COLON risultano proporzionate tra gruppi di genere. | | | |
| | %sex | 97,4% | 2,6% | 100,0% | | | | |
| | % K COLON | 64,4% | 100,0% | 65,0% | | | | |
| Total | Count | 59 | 1 | 60 | | | | |
| | %sex | 98,3% | 1,7% | 100,0% | | | | |
| | % K COLON | 100,0% | 100,0% | 100,0% | | | | |

**SEX1M * K MAMMELLA tubulo-papillare Crosstabulation**

| | | K MAMMELLA | | | Chi-SquareTests | | | |
|---|---|---|---|---|---|---|---|---|
| sex | | 0=N | 1=S | Total | | Value | df | Sig. |
| 0=F | Count | 18 | 3 | 21 | Chi-Square | 5,865 | 1 | **,015** |
| | %sex | 85,7% | 14,3% | 100,0% | N | 60 | | |
| | % K MAMMELLA | 31,6% | 100,0% | 35,0% | | | | |
| 1=M | Count | 39 | 0 | 39 | Commento: il test certifica che le occorrenze di K MAMMELLA risultano per logica statisticamente disproporzionate tra gruppi di genere. | | | |
| | %sex | 100,0% | ,0% | 100,0% | | | | |
| | % K MAMMELLA | 68,4% | ,0% | 65,0% | | | | |
| Total | Count | 57 | 3 | 60 | | | | |
| | %sex | 95,0% | 5,0% | 100,0% | | | | |
| | % K MAMMELLA | 100,0% | 100,0% | 100,0% | | | | |

**SEX1M * MELANOMA pregresso Crosstabulation**

| | | MELANOMA | | | Chi-SquareTests | | | |
|---|---|---|---|---|---|---|---|---|
| sex | | 0=N | 1=S | Total | | Value | df | Sig. |
| 0=F | Count | 20 | 1 | 21 | Chi-Square | ,188 | 1 | ,664 |
| | %sex | 95,2% | 4,8% | 100,0% | N | 60 | | |
| | % MELANOMA | 35,7% | 25,0% | 35,0% | | | | |
| 1=M | Count | 36 | 3 | 39 | Commento: il test certifica che le occorrenze di MELANOMA risultano proporzionate tra gruppi di genere | | | |
| | %sex | 92,3% | 7,7% | 100,0% | | | | |
| | % MELANOMA | 64,3% | 75,0% | 65,0% | | | | |
| Total | Count | 56 | 4 | 60 | | | | |
| | %sex | 93,3% | 6,7% | 100,0% | | | | |
| | % MELANOMA | 100,0% | 100,0% | 100,0% | | | | |

**SEX1M * linfoma non-hodgkin Crosstabulation**

| | | linfoma non-hodgkin | | | Chi-SquareTests | | | |
|---|---|---|---|---|---|---|---|---|
| sex | | 0=N | 1=S | Total | | Value | df | Sig. |
| 0=F | Count | 21 | 0 | 21 | Chi-Square | 1,114 | 1 | ,291 |
| | %sex | 100,0% | ,0% | 100,0% | N | 60 | | |
| | % linfoma | 36,2% | ,0% | 35,0% | | | | |
| 1=M | Count | 37 | 2 | 39 | Commento: il test certifica che le occorrenze di linfoma non-hodgkin risultano proporzionate tra gruppi di genere | | | |
| | %sex | 94,9% | 5,1% | 100,0% | | | | |
| | % linfoma | 63,8% | 100,0% | 65,0% | | | | |
| Total | Count | 58 | 2 | 60 | | | | |
| | %sex | 96,7% | 3,3% | 100,0% | | | | |
| | % linfoma | 100,0% | 100,0% | 100,0% | | | | |

**SEX1M * K tiroide istotipo papillare Crosstabulation**

| | | K tiroide | | | Chi-SquareTests | | | |
|---|---|---|---|---|---|---|---|---|
| sex | | 0=N | 1=S | Total | | Value | df | Sig. |
| 0=F | Count | 18 | 3 | 21 | Chi-Square | 5,865 | 1 | **,015** |
| | %sex | 85,7% | 14,3% | 100,0% | N | 60 | | |
| | % K tiroide | 31,6% | 100,0% | 35,0% | | | | |
| 1=M | Count | 39 | 0 | 39 | Commento: il test certifica che le occorrenze di K tiroide risultano statisticamente disproporzionate tra gruppi di genere. | | | |
| | %sex | 100,0% | ,0% | 100,0% | | | | |
| | % K tiroide | 68,4% | ,0% | 65,0% | | | | |
| Total | Count | 57 | 3 | 60 | | | | |
| | %sex | 95,0% | 5,0% | 100,0% | | | | |
| | % K tiroide | 100,0% | 100,0% | 100,0% | | | | |

**SEX1M * K rene a cellule chiare Crosstabulation**

| | | K rene | | | Chi-SquareTests | | | |
|---|---|---|---|---|---|---|---|---|
| sex | | 0=N | 1=S | Total | | Value | df | Sig. |
| 0=F | Count | 18 | 3 | 21 | Chi-Square | ,548 | 1 | ,459 |
| | %sex | 85,7% | 14,3% | 100,0% | N | 60 | | |
| | % K rene | 31,6% | 100,0% | 35,0% | | | | |
| 1=M | Count | 39 | 0 | 39 | Commento: il test certifica che le occorrenze di K rene risultano proporzionate tra gruppi di genere | | | |
| | %sex | 100,0% | ,0% | 100,0% | | | | |
| | % K rene | 68,4% | ,0% | 65,0% | | | | |
| Total | Count | 57 | 3 | 60 | | | | |
| | %sex | 95,0% | 5,0% | 100,0% | | | | |
| | % K rene | 100,0% | 100,0% | 100,0% | | | | |

**SEX1M * MGUS Monoclonal gammopathy of undetermined significance Crosstabulation**

| | | MGUS | | | Chi-SquareTests | | | |
|---|---|---|---|---|---|---|---|---|
| sex | | 0=N | 1=S | Total | | Value | df | Sig. |
| 0=F | Count | 21 | 0 | 21 | Chi-Square | ,548 | 1 | ,459 |
| | %sex | 100,0% | ,0% | 100,0% | N | 60 | | |
| | % MGUS | 35,6% | ,0% | 35,0% | | | | |
| 1=M | Count | 38 | 1 | 39 | Commento: il test certifica che le occorrenze di MGUS risultano proporzionate tra gruppi di genere | | | |
| | %sex | 97,4% | 2,6% | 100,0% | | | | |
| | % MGUS | 64,4% | 100,0% | 65,0% | | | | |
| Total | Count | 59 | 1 | 60 | | | | |
| | %sex | 98,3% | 1,7% | 100,0% | | | | |
| | % MGUS | 100,0% | 100,0% | 100,0% | | | | |

**SEX1M * IBD Crosstabulation**

| | | IBD | | |
|---|---|---|---|---|
| sex | | 0=N | 1=S | Total |
| 0=F | Count | 21 | 0 | 21 |
| | %sex | 100,0% | ,0% | 100,0% |
| | % IBD | 35,6% | ,0% | 35,0% |
| 1=M | Count | 38 | 1 | 39 |
| | %sex | 97,4% | 2,6% | 100,0% |
| | % IBD | 64,4% | 100,0% | 65,0% |
| Total | Count | 59 | 1 | 60 |
| | %sex | 98,3% | 1,7% | 100,0% |
| | % IBD | 100,0% | 100,0% | 100,0% |

| Chi-SquareTests | | | |
|---|---|---|---|
| | Value | df | Sig. |
| Chi-Square | ,548 | 1 | ,459 |
| N | 60 | | |

Commento: il test certifica che le occorrenze di IBD risultano proporzionate tra gruppi di genere

**SEX1M * COMORBIDITA' CARDIOVASCOLARE Crosstabulation**

| | | COMORBIDITA' VASC. | | |
|---|---|---|---|---|
| sex | | 0=N | 1=S | Total |
| 0=F | Count | 12 | 9 | 21 |
| | %sex | 57,1% | 42,9% | 100,0% |
| | %COMORBIDITA'VASC. | 32,4% | 39,1% | 35,0% |
| 1=M | Count | 25 | 14 | 39 |
| | %sex | 64,1% | 35,9% | 100,0% |
| | %COMORBIDITA'VASC. | 67,6% | 60,9% | 65,0% |
| Total | Count | 37 | 23 | 60 |
| | %sex | 61,7% | 38,3% | 100,0% |
| | %COMORBIDITA'VASC. | 100,0% | 100,0% | 100,0% |

| Chi-SquareTests | | | |
|---|---|---|---|
| | Value | df | Sig. |
| Chi-Square | ,280 | 1 | ,597 |
| N | 60 | | |

Commento: il test certifica che le occorrenze di COMORBIDITA' VASC risultano proporzionate tra gruppi di genere

**SEX1M * COMORBIDITA' METABOLICA Crosstabulation**

| | | COMORBIDITA' MET | | |
|---|---|---|---|---|
| sex | | 0=N | 1=S | Total |
| 0=F | Count | 17 | 4 | 21 |
| | %sex | 81,0% | 19,0% | 100,0% |
| | % COMORBIDITA' MET | 35,4% | 33,3% | 35,0% |
| 1=M | Count | 31 | 8 | 39 |
| | %sex | 79,5% | 20,5% | 100,0% |
| | % COMORBIDITA' MET | 64,6% | 66,7% | 65,0% |
| Total | Count | 48 | 12 | 60 |
| | %sex | 80,0% | 20,0% | 100,0% |
| | % COMORBIDITA' MET | 100,0% | 100,0% | 100,0% |

| Chi-SquareTests | | | |
|---|---|---|---|
| | Value | df | Sig. |
| Chi-Square | ,018 | 1 | ,892 |
| N | 60 | | |

Commento: il test certifica che le occorrenze di COMORBIDITA' MET risultano proporzionate tra gruppi di genere

**SEX1M * IPERCOLESTEROLEMIA Crosstabulation**

| | | IPERCOLESTEROLEMIA | | |
|---|---|---|---|---|
| sex | | 0=N | 1=S | Total |
| 0=F | Count | 19 | 2 | 21 |
| | %sex | 90,5% | 9,5% | 100,0% |
| | % IPERCOLESTEROL. | 37,3% | 22,2% | 35,0% |
| 1=M | Count | 32 | 7 | 39 |
| | %sex | 82,1% | 17,9% | 100,0% |
| | % IPERCOLESTEROL. | 62,7% | 77,8% | 65,0% |
| Total | Count | 51 | 9 | 60 |
| | %sex | 85,0% | 15,0% | 100,0% |
| | % IPERCOLESTEROL. | 100,0% | 100,0% | 100,0% |

| Chi-SquareTests | | | |
|---|---|---|---|
| | Value | df | Sig. |
| Chi-Square | ,760 | 1 | ,383 |
| N | 60 | | |

Commento: il test certifica che le occorrenze di IPERCOLESTEROLEMIA risultano proporzionate tra gruppi di genere

**SEX1M * IPERTENSIONE Crosstabulation**

| | | IPERTENSIONE | | |
|---|---|---|---|---|
| sex | | 0=N | 1=S | Total |
| 0=F | Count | 14 | 7 | 21 |
| | %sex | 66,7% | 33,3% | 100,0% |
| | % IPERTENSIONE | 36,8% | 31,8% | 35,0% |
| 1=M | Count | 24 | 15 | 39 |
| | %sex | 61,5% | 38,5% | 100,0% |
| | % IPERTENSIONE | 63,2% | 68,2% | 65,0% |
| Total | Count | 38 | 22 | 60 |
| | %sex | 63,3% | 36,7% | 100,0% |
| | % IPERTENSIONE | 100,0% | 100,0% | 100,0% |

| Chi-SquareTests | | | |
|---|---|---|---|
| | Value | df | Sig. |
| Chi-Square | ,155 | 1 | ,694 |
| N | 60 | | |

Commento: il test certifica che le occorrenze di IPERTENSIONE risultano proporzionate tra gruppi di genere

**SEX1M * DIABETE Crosstabulation**

| | | DIABETE | | | Chi-SquareTests | | | |
|---|---|---|---|---|---|---|---|---|
| sex | | 0=N | 1=S | Total | | Value | df | Sig. |
| 0=F | Count | 19 | 2 | 21 | Chi-Square | ,144 | 1 | ,704 |
| | %sex | 90,5% | 9,5% | 100,0% | N | 60 | | |
| | % DIABETE | 35,8% | 28,6% | 35,0% | | | | |
| 1=M | Count | 34 | 5 | 39 | Commento: il test certifica che le occorrenze di DIABETE risultano proporzionate tra gruppi di genere | | | |
| | %sex | 87,2% | 12,8% | 100,0% | | | | |
| | % DIABETE | 64,2% | 71,4% | 65,0% | | | | |
| Total | Count | 53 | 7 | 60 | | | | |
| | %sex | 88,3% | 11,7% | 100,0% | | | | |
| | % DIABETE | 100,0% | 100,0% | 100,0% | | | | |

**SEX1M * COMORBIDITA' INFETTIVE Crosstabulation**

| | | COMORBIDITA'INF | | | Chi-SquareTests | | | |
|---|---|---|---|---|---|---|---|---|
| sex | | 0=N | 1=S | Total | | Value | df | Sig. |
| 0=F | Count | 19 | 2 | 21 | Chi-Square | ,060 | 1 | ,807 |
| | %sex | 90,5% | 9,5% | 100,0% | N | 60 | | |
| | % COMORBIDITA' INF | 34,5% | 40,0% | 35,0% | | | | |
| 1=M | Count | 36 | 3 | 39 | Commento: il test certifica che le occorrenze di COMORBIDITA' INF risultano proporzionate tra gruppi di genere | | | |
| | %sex | 92,3% | 7,7% | 100,0% | | | | |
| | % COMORBIDITA' INF | 65,5% | 60,0% | 65,0% | | | | |
| Total | Count | 55 | 5 | 60 | | | | |
| | %sex | 91,7% | 8,3% | 100,0% | | | | |
| | % COMORBIDITA' INF | 100,0% | 100,0% | 100,0% | | | | |

**SEX1M * naive Crosstabulation**

| | | naive | | | Chi-SquareTests | | | |
|---|---|---|---|---|---|---|---|---|
| sex | | 0=N | 1=S | Total | | Value | df | Sig. |
| 0=F | Count | 4 | 17 | 21 | Chi-Square | ,913 | 1 | ,339 |
| | %sex | 19,0% | 81,0% | 100,0% | N | 60 | | |
| | % naive | 50,0% | 32,7% | 35,0% | | | | |
| 1=M | Count | 4 | 35 | 39 | Commento: il test certifica che le occorrenze di naive risultano proporzionate tra gruppi di genere | | | |
| | %sex | 10,3% | 89,7% | 100,0% | | | | |
| | % naive | 50,0% | 67,3% | 65,0% | | | | |
| Total | Count | 8 | 52 | 60 | | | | |
| | %sex | 13,3% | 86,7% | 100,0% | | | | |
| | % naive | 100,0% | 100,0% | 100,0% | | | | |

**SEX1M * no naive Crosstabulation**

| | | No naive | | | Chi-SquareTests | | | |
|---|---|---|---|---|---|---|---|---|
| sex | | 0=N | 1=S | Total | | Value | df | Sig. |
| 0=F | Count | 17 | 4 | 21 | Chi-Square | ,913 | 1 | ,339 |
| | %sex | 81,0% | 19,0% | 100,0% | N | 60 | | |
| | % no naive | 32,7% | 50,0% | 35,0% | | | | |
| 1=M | Count | 35 | 4 | 39 | Commento: il test certifica che le occorrenze di no naive risultano proporzionate tra gruppi di genere | | | |
| | %sex | 89,7% | 10,3% | 100,0% | | | | |
| | % no naive | 67,3% | 50,0% | 65,0% | | | | |
| Total | Count | 52 | 8 | 60 | | | | |
| | %sex | 86,7% | 13,3% | 100,0% | | | | |
| | % no naive | 100,0% | 100,0% | 100,0% | | | | |

**SEX1M * TERAPIE LOCALI PRECEDENTI (corticosteroidi topici) Crosstabulation**

| | | TERAPIE LOC | | | Chi-SquareTests | | | |
|---|---|---|---|---|---|---|---|---|
| sex | | 0=N | 1=S | Total | | Value | df | Sig. |
| 0=F | Count | 5 | 16 | 21 | Chi-Square | 1,187 | 1 | ,276 |
| | %sex | 23,8% | 76,2% | 100,0% | N | 60 | | |
| | % TERAPIE LOC | 50,0% | 32,0% | 35,0% | | | | |
| 1=M | Count | 5 | 34 | 39 | Commento: il test certifica che le occorrenze di TERAPIE LOCALI PRECEDENTI risultano proporzionate tra gruppi di genere | | | |
| | %sex | 12,8% | 87,2% | 100,0% | | | | |
| | % TERAPIE LOC | 50,0% | 68,0% | 65,0% | | | | |
| Total | Count | 10 | 50 | 60 | | | | |
| | %sex | 16,7% | 83,3% | 100,0% | | | | |
| | % TERAPIE LOC | 100,0% | 100,0% | 100,0% | | | | |

**SEX1M * TERAPIE SISTEMICHE PRECEDENTI  Crosstabulation**

| | | TERAPIE SIST | | |
|---|---|---|---|---|
| sex | | 0=N | 1=S | Total |
| 0=F | Count | 17 | 4 | 21 |
| | %sex | 81,0% | 19,0% | 100,0% |
| | % TERAPIE SIST | 32,7% | 50,0% | 35,0% |
| 1=M | Count | 35 | 4 | 39 |
| | %sex | 89,7% | 10,3% | 100,0% |
| | % TERAPIE SIST | 67,3% | 50,0% | 65,0% |
| Total | Count | 52 | 8 | 60 |
| | %sex | 86,7% | 13,3% | 100,0% |
| | % TERAPIE SIST | 100,0% | 100,0% | 100,0% |

| Chi-SquareTests | | | |
|---|---|---|---|
| | Value | df | Sig. |
| Chi-Square | ,913 | 1 | ,339 |
| N | 60 | | |

Commento: il test certifica che le occorrenze di TERAPIE SIST risultano proporzionate tra gruppi di genere

**SEX1M * anti-TNF  Crosstabulation**

| | | anti-TNF | | |
|---|---|---|---|---|
| sex | | 0=N | 1=S | Total |
| 0=F | Count | 20 | 1 | 21 |
| | %sex | 95,2% | 4,8% | 100,0% |
| | % anti-TNF | 34,5% | 50,0% | 35,0% |
| 1=M | Count | 38 | 1 | 39 |
| | %sex | 97,4% | 2,6% | 100,0% |
| | % anti-TNF | 65,5% | 50,0% | 65,0% |
| Total | Count | 58 | 2 | 60 |
| | %sex | 96,7% | 3,3% | 100,0% |
| | % anti-TNF | 100,0% | 100,0% | 100,0% |

| Chi-SquareTests | | | |
|---|---|---|---|
| | Value | df | Sig. |
| Chi-Square | ,205 | 1 | ,651 |
| N | 60 | | |

Commento: il test certifica che le occorrenze di anti-TNF risultano proporzionate tra gruppi di genere

**SEX1M * CICLOSPORINA Crosstabulation**

| | | CICLOSPORINA | | |
|---|---|---|---|---|
| sex | | 0=N | 1=S | Total |
| 0=F | Count | 17 | 4 | 21 |
| | %sex | 81,0% | 19,0% | 100,0% |
| | % CICLOSPORINA | 30,9% | 80,0% | 35,0% |
| 1=M | Count | 38 | 1 | 39 |
| | %sex | 97,4% | 2,6% | 100,0% |
| | % CICLOSPORINA | 69,1% | 20,0% | 65,0% |
| Total | Count | 55 | 5 | 60 |
| | %sex | 91,7% | 8,3% | 100,0% |
| | % CICLOSPORINA | 100,0% | 100,0% | 100,0% |

| Chi-SquareTests | | | |
|---|---|---|---|
| | Value | df | Sig. |
| Chi-Square | 4,855 | 1 | **,028** |
| N | 60 | | |

Commento: il test certifica che le occorrenze di CICLOSPORINA risultano statisticamente disproporzionate tra gruppi di genere.

**SEX1M * METOTREXATE  Crosstabulation**

| | | METOTREXATE | | |
|---|---|---|---|---|
| sex | | 0=N | 1=S | Total |
| 0=F | Count | 21 | 0 | 21 |
| | %sex | 100,0% | ,0% | 100,0% |
| | % METOTREXATE | 36,2% | ,0% | 35,0% |
| 1=M | Count | 37 | 2 | 39 |
| | %sex | 94,9% | 5,1% | 100,0% |
| | % METOTREXATE | 63,8% | 100,0% | 65,0% |
| Total | Count | 58 | 2 | 60 |
| | %sex | 96,7% | 3,3% | 100,0% |
| | % METOTREXATE | 100,0% | 100,0% | 100,0% |

| Chi-SquareTests | | | |
|---|---|---|---|
| | Value | df | Sig. |
| Chi-Square | 1,114 | 1 | ,291 |
| N | 60 | | |

Commento: il test certifica che le occorrenze di METOTREXATE risultano proporzionate tra gruppi di genere

**SEX1M * CORTICOSTEROIDE SISTEMICO Crosstabulation**

| | | CORTICOSTEROIDE SIST | | |
|---|---|---|---|---|
| sex | | 0=N | 1=S | Total |
| 0=F | Count | 21 | 0 | 21 |
| | %sex | 100,0% | ,0% | 100,0% |
| | % CORTICOSTEROIDE | 36,2% | ,0% | 35,0% |
| 1=M | Count | 37 | 2 | 39 |
| | %sex | 94,9% | 5,1% | 100,0% |
| | % CORTICOSTEROIDE | 63,8% | 100,0% | 65,0% |
| Total | Count | 58 | 2 | 60 |
| | %sex | 96,7% | 3,3% | 100,0% |
| | % CORTICOSTEROIDE | 100,0% | 100,0% | 100,0% |

| Chi-SquareTests | | | |
|---|---|---|---|
| | Value | df | Sig. |
| Chi-Square | ,548 | 1 | ,459 |
| N | 60 | | |

Commento: il test certifica che le occorrenze di CORTICOSTEROIDE SIST risultano proporzionate tra gruppi di genere

**SEX1M * diarrea Crosstabulation**

| sex | | diarrea 0=N | 1=S | Total |
|---|---|---|---|---|
| 0=F | Count | 16 | 5 | 21 |
| | %sex | 76,2% | 23,8% | 100,0% |
| | % diarrea | 33,3% | 41,7% | 35,0% |
| 1=M | Count | 32 | 7 | 39 |
| | %sex | 82,1% | 17,9% | 100,0% |
| | % diarrea | 66,7% | 58,3% | 65,0% |
| Total | Count | 48 | 12 | 60 |
| | %sex | 80,0% | 20,0% | 100,0% |
| | % diarrea | 100,0% | 100,0% | 100,0% |

Chi-SquareTests

| | Value | df | Sig. |
|---|---|---|---|
| Chi-Square | ,293 | 1 | ,588 |
| N | 60 | | |

Commento: il test certifica che le occorrenze di diarrea risultano proporzionate tra gruppi di genere

**SEX1M * nausea Crosstabulation**

| sex | | nausea 0=N | 1=S | Total |
|---|---|---|---|---|
| 0=F | Count | 19 | 2 | 21 |
| | %sex | 90,5% | 9,5% | 100,0% |
| | % nausea | 34,5% | 40,0% | 35,0% |
| 1=M | Count | 36 | 3 | 39 |
| | %sex | 92,3% | 7,7% | 100,0% |
| | % nasuea | 65,5% | 60,0% | 65,0% |
| Total | Count | 55 | 5 | 60 |
| | %sex | 91,7% | 8,3% | 100,0% |
| | % nasuea | 100,0% | 100,0% | 100,0% |

Chi-SquareTests

| | Value | df | Sig. |
|---|---|---|---|
| Chi-Square | ,060 | 1 | ,807 |
| N | 60 | | |

Commento: il test certifica che le occorrenze di nausea risultano proporzionate tra gruppi di genere

**SEX1M * pesantezza gastrica Crosstabulation**

| sex | | pesantezza gastrica 0=N | 1=S | Total |
|---|---|---|---|---|
| 0=F | Count | 19 | 2 | 21 |
| | %sex | 90,5% | 9,5% | 100,0% |
| | % pesantezza gastrica | 34,5% | 40,0% | 35,0% |
| 1=M | Count | 36 | 3 | 39 |
| | %sex | 92,3% | 7,7% | 100,0% |
| | % pesantezza gastrica | 65,5% | 60,0% | 65,0% |
| Total | Count | 55 | 5 | 60 |
| | %sex | 91,7% | 8,3% | 100,0% |
| | % pesantezza gastrica | 100,0% | 100,0% | 100,0% |

Chi-SquareTests

| | Value | df | Sig. |
|---|---|---|---|
| Chi-Square | ,144 | 1 | ,704 |
| N | 60 | | |

Commento: il test certifica che le occorrenze di pesantezza gastrica risultano proporzionate tra gruppi di genere

**SEX1M * insonnia Crosstabulation**

| sex | | insonnia 0=N | 1=S | Total |
|---|---|---|---|---|
| 0=F | Count | 19 | 2 | 21 |
| | %sex | 90,5% | 9,5% | 100,0% |
| | % insonnia | 35,2% | 33,3% | 35,0% |
| 1=M | Count | 35 | 4 | 39 |
| | %sex | 89,7% | 10,3% | 100,0% |
| | % insonnia | 64,8% | 66,7% | 65,0% |
| Total | Count | 54 | 6 | 60 |
| | %sex | 90,0% | 10,0% | 100,0% |
| | % insonnia | 100,0% | 100,0% | 100,0% |

Chi-SquareTests

| | Value | df | Sig. |
|---|---|---|---|
| Chi-Square | ,144 | 1 | ,704 |
| N | 60 | | |

Commento: il test certifica che le occorrenze di insonnia risultano proporzionate tra gruppi di genere

**SEX1M * tristezza Crosstabulation**

| sex | | i tristezza 0=N | 1=S | Total |
|---|---|---|---|---|
| 0=F | Count | 19 | 2 | 21 |
| | %sex | 90,5% | 9,5% | 100,0% |
| | % tristezza | 32,8% | 100,0% | 35,0% |
| 1=M | Count | 39 | 0 | 39 |
| | %sex | 100,0% | ,0% | 100,0% |
| | % tristezza | 67,2% | ,0% | 65,0% |
| Total | Count | 58 | 2 | 60 |
| | %sex | 96,7% | 3,3% | 100,0% |
| | % tristezza | 100,0% | 100,0% | 100,0% |

Chi-SquareTests

| | Value | df | Sig. |
|---|---|---|---|
| Chi-Square | 3,842 | 1 | ,051 |
| N | 60 | | |

Commento: il test certifica che le occorrenze di tristezza risultano al limite proporzionate tra gruppi di genere

**SEX1M * sonnolenza Crosstabulation**

| | | sonnolenza | | | Chi-SquareTests | | | |
|---|---|---|---|---|---|---|---|---|
| sex | | 0=N | 1=S | Total | | Value | df | Sig. |
| 0=F | Count | 21 | 0 | 21 | Chi-Square | 1,114 | 1 | ,291 |
| | %sex | 100,0% | ,0% | 100,0% | N | 60 | | |
| | % sonnolenza | 36,2% | ,0% | 35,0% | | | | |
| 1=M | Count | 37 | 2 | 39 | Commento: il test certifica che le occorrenze di sonnolenza risultano proporzionate tra gruppi di genere | | | |
| | %sex | 94,9% | 5,1% | 100,0% | | | | |
| | % sonnolenza | 63,8% | 100,0% | 65,0% | | | | |
| Total | Count | 58 | 2 | 60 | | | | |
| | %sex | 96,7% | 3,3% | 100,0% | | | | |
| | % sonnolenza | 100,0% | 100,0% | 100,0% | | | | |

**SEX1M * cambio dell'umore Crosstabulation**

| | | cambio dell'umore | | | Chi-SquareTests | | | |
|---|---|---|---|---|---|---|---|---|
| sex | | 0=N | 1=S | Total | | Value | df | Sig. |
| 0=F | Count | 19 | 2 | 21 | Chi-Square | 1,392 | 1 | ,238 |
| | %sex | 90,5% | 9,5% | 100,0% | N | 60 | | |
| | % cambio dell'umore | 33,3% | 66,7% | 35,0% | | | | |
| 1=M | Count | 38 | 1 | 39 | Commento: il test certifica che le occorrenze di cambio dell'umore risultano proporzionate tra gruppi di genere | | | |
| | %sex | 97,4% | 2,6% | 100,0% | | | | |
| | % cambio dell'umore | 66,7% | 33,3% | 65,0% | | | | |
| Total | Count | 57 | 3 | 60 | | | | |
| | %sex | 95,0% | 5,0% | 100,0% | | | | |
| | % cambio dell'umore | 100,0% | 100,0% | 100,0% | | | | |

**SEX1M * calo della libido Crosstabulation**

| | | calo della libido | | | Chi-SquareTests | | | |
|---|---|---|---|---|---|---|---|---|
| sex | | 0=N | 1=S | Total | | Value | df | Sig. |
| 0=F | Count | 21 | 0 | 21 | Chi-Square | ,548 | 1 | ,459 |
| | %sex | 100,0% | ,0% | 100,0% | N | 60 | | |
| | % calo della libido | 35,6% | ,0% | 35,0% | | | | |
| 1=M | Count | 38 | 1 | 39 | Commento: il test certifica che le occorrenze di calo della libido risultano proporzionate tra gruppi di genere | | | |
| | %sex | 97,4% | 2,6% | 100,0% | | | | |
| | % calo della libido | 64,4% | 100,0% | 65,0% | | | | |
| Total | Count | 59 | 1 | 60 | | | | |
| | %sex | 98,3% | 1,7% | 100,0% | | | | |
| | % calo della libido | 100,0% | 100,0% | 100,0% | | | | |

**SEX1M * alterazioni della percezione Crosstabulation**

| | | alterazioni della percezione | | | Chi-SquareTests | | | |
|---|---|---|---|---|---|---|---|---|
| sex | | 0=N | 1=S | Total | | Value | df | Sig. |
| 0=F | Count | 21 | 0 | 21 | Chi-Square | ,548 | 1 | ,459 |
| | %sex | 100,0% | ,0% | 100,0% | N | 60 | | |
| | % alterazioni percezione | 35,6% | ,0% | 35,0% | | | | |
| 1=M | Count | 38 | 1 | 39 | Commento: il test certifica che le occorrenze di alterazioni della percezione risultano proporzionate tra gruppi di genere | | | |
| | %sex | 97,4% | 2,6% | 100,0% | | | | |
| | % alterazioni percezione | 64,4% | 100,0% | 65,0% | | | | |
| Total | Count | 59 | 1 | 60 | | | | |
| | %sex | 98,3% | 1,7% | 100,0% | | | | |
| | % alterazioni percezione | 100,0% | 100,0% | 100,0% | | | | |

# CONFRONTI LONGITUDINALI (factor1=T0;T4;T6;T12 mesi)

| Descriptive Statistics | | | | ANOVA per misure multiple | | | | | | Bonferroni test | | | |
|---|---|---|---|---|---|---|---|---|---|---|---|---|---|
| Factor1 | Mean | sd | N | | Sumofsquares | df | Mean$^2$ | F | Sig. | Sig. | **0** | **4** | **6** |
| PESO_0 | 77,6 | 14,4 | 60 | Factor1 | 894,413 | 3 | 298,138 | 9,549 | **,000** | 4 | **,000** | | |
| PESO_4 | 75,2 | 12,7 | 60 | Error | 5526,338 | 177 | 31,222 | | | 6 | **,000** | **,041** | |
| PESO_6 | 74,4 | 12,2 | 60 | | | | | | | 12 | **,002** | ,210 | .730 |
| PESO_12 | 72,2 | 14,2 | 60 | | | | | | | | | | |

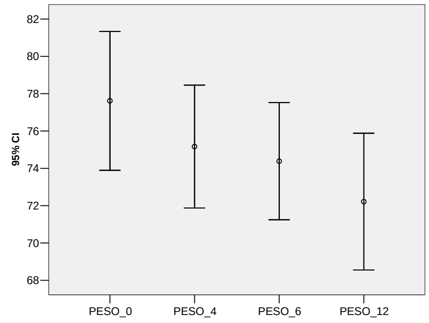


**Commento:** Come è evidente il trend del parametro nel tempo risulta significativamente discendente.

| Descriptive Statistics | | | | ANOVA per misure multiple | | | | | | Bonferroni test | | | |
|---|---|---|---|---|---|---|---|---|---|---|---|---|---|
| Factor1 | Mean | sd | N | | SumofSquares | df | Mean$^2$ | F | Sig. | Sig | **0** | **4** | **6** |
| BMI_0 | 26,02 | 4,74 | 60 | factor1 | 93,259 | 3 | 31,086 | 12,096 | **,000** | 4 | **,000** | | |
| BMI_4 | 25,19 | 4,11 | 60 | Error | 454,900 | 177 | 2,570 | | | 6 | **,000** | **,029** | |
| BMI_6 | 24,91 | 3,79 | 60 | | | | | | | 12 | **,000** | **,124** | **,636** |
| BMI_12 | 24,28 | 4,75 | 60 | | | | | | | | | | |

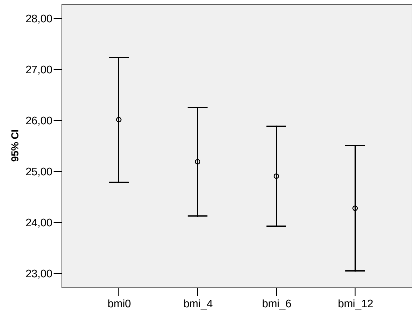


**Commento:** Come è evidente il trend del parametro nel tempo risulta significativamente discendente.

| Descriptive Statistics | | | | ANOVA per misure multiple | | | | | | Bonferroni test | | | |
|---|---|---|---|---|---|---|---|---|---|---|---|---|---|
| Factor1 | Mean | sd | N | | SumofSquares | df | Mean$^2$ | F | Sig. | Sig | **0** | **4** | **6** |
| PASI_0 | 12,17 | 11,010 | 60 | factor1 | 4637,578 | 3 | 1545,859 | 53,752 | **,000** | 4 | **,000** | | |
| PASI_4 | 2,97 | 3,682 | 60 | Error | 5090,359 | 177 | 28,759 | | | 6 | **,000** | **,002** | |
| PASI_6 | 1,74 | 2,539 | 60 | | | | | | | 12 | **,000** | **,041** | **1,00** |
| PASI_12 | 1,57 | 2,459 | 60 | | | | | | | | | | |

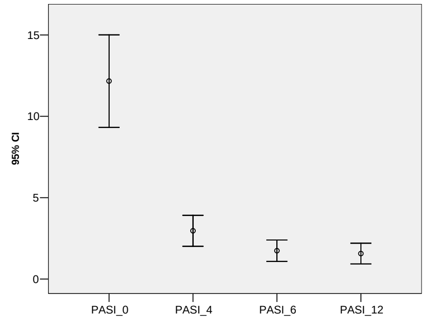


**Commento:** Come è evidente il trend del parametro nel tempo risulta significativamente discendente, già al 4° mese!

**Descriptive Statistics**

| Factor1 | Mean | sd | N |
|---|---|---|---|
| NAPSITOT_0 | 28,02 | 32,64 | 60 |
| NAPSI TOT_4 | 13,68 | 23,50 | 60 |
| NAPSI TOT_6 | 9,10 | 21,76 | 60 |
| NAPSITOT_12 | 6,53 | 18,17 | 60 |

ANOVA per misure multiple

| | SumofSquares | df | Mean$^2$ | F | Sig. |
|---|---|---|---|---|---|
| factor1 | 16553,03 | 3 | 5517,68 | 30,26 | ,000 |
| Error | 32273,97 | 177 | 182,34 | | |

**Bonferroni test**

| **Sig** | **0** | **4** | **6** |
|---|---|---|---|
| **4** | **,000** | | |
| **6** | **,000** | **,018** | |
| **12** | **,000** | **,005** | **,932** |

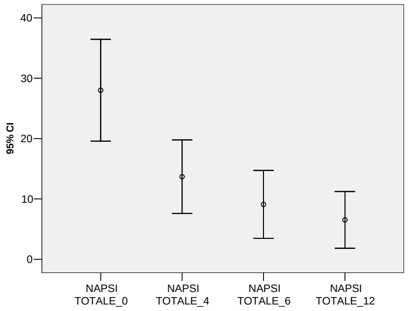


**Commento:** Come è evidente il trend del parametro nel tempo risulta significativamente discendente, già al 4° mese!

**Descriptive Statistics**

| Factor1 | Mean | sd | N |
|---|---|---|---|
| DLQI_0 | 6,85 | 6,62 | 60 |
| DLQI_4 | 1,22 | 2,59 | 60 |
| DLQI_6 | ,75 | 1,66 | 60 |
| DLQI_12 | ,53 | 1,11 | 60 |

ANOVA per misure multiple

| | SumofSquares | df | Mean$^2$ | F | Sig. |
|---|---|---|---|---|---|
| factor1 | 1643,646 | 3 | 547,882 | 43,300 | ,000 |
| Error | 2239,604 | 177 | 12,653 | | |

**Bonferroni test**

| Sig | **0** | **4** | **6** |
|---|---|---|---|
| **4** | **,000** | | |
| **6** | **,000** | **1,00** | |
| **12** | **,000** | **,406** | **1,00** |

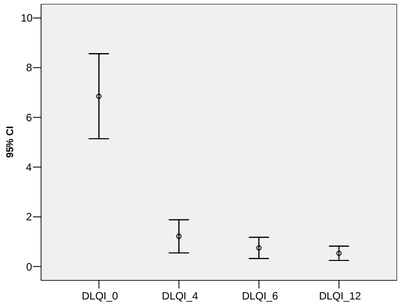


**Commento:** Come è evidente il trend del parametro nel tempo risulta significativamente discendente, già al 4° mese!

**Descriptive Statistics**

| Factor1 | Mean | sd | N |
|---|---|---|---|
| itch_0 | 7,42 | 2,54 | 60 |
| itch_4 | 1,17 | 2,05 | 60 |
| itch_6 | 1,50 | 2,57 | 60 |
| itch_12 | ,93 | 1,84 | 60 |

ANOVA per misure multiple

| | SumofSquares | df | Mean$^2$ | F | Sig. |
|---|---|---|---|---|---|
| factor1 | 1748,846 | 3 | 582,949 | 140,977 | ,000 |
| Error | 731,904 | 177 | 4,135 | | |

**Bonferroni test**

| Sig | **0** | **4** | **6** |
|---|---|---|---|
| **4** | **,000** | | |
| **6** | **,000** | **1,00** | |
| **12** | **,000** | **1,00** | **,758** |

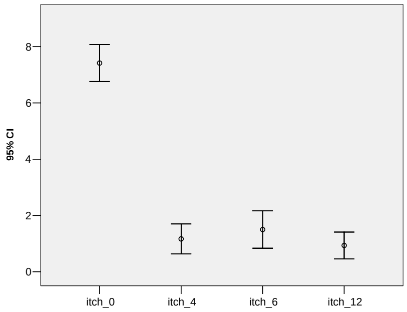


**Commento:** Come è evidente il trend del parametro nel tempo risulta significativamente discendente, già al 4° mese!

**Descriptive Statistics**

| Factor1 | Mean | sd | N |
|---|---|---|---|
| PAINVAS_0 | 19,78 | 28,09 | 60 |
| PAIN VAS_4 | 8,00 | 20,98 | 60 |
| PAIN VAS_6 | 6,33 | 19,04 | 60 |
| PAINVAS_12 | 3,97 | 10,08 | 60 |

ANOVA per misure multiple

| | SumofSquares | df | Mean² | F | Sig. |
|---|---|---|---|---|---|
| factor1 | 8918,45 | 3 | 2972,82 | 12,10 | **,000** |
| Error | 43478,80 | 177 | 245,64 | | |

**Bonferroni test**

| Sig | 0 | 4 | 6 |
|---|---|---|---|
| **4** | **,002** | | |
| **6** | **,001** | 1,00 | |
| **12** | **,000** | ,659 | **1,00** |

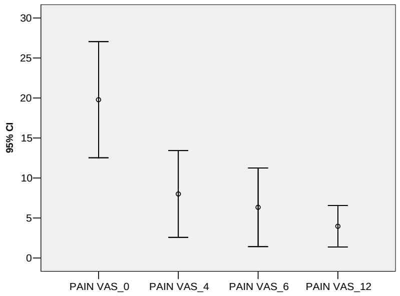


**Commento:** Come è evidente il trend del parametro nel tempo risulta significativamente discendente già al 4° mese!

**Descriptive Statistics**

| Factor1 | Mean | sd | N |
|---|---|---|---|
| GLICEMIA_0 | 103,13 | 35,24 | 60 |
| GLICEMIA_4 | 96,25 | 18,99 | 60 |
| GLICEMIA_6 | 95,51 | 18,56 | 60 |
| GLICEMIA_12 | 93,33 | 11,72 | 60 |

ANOVA per misure multiple

| | SumofSquares | df | Mean² | F | Sig. |
|---|---|---|---|---|---|
| factor1 | 3233,954 | 3 | 1077,99 | 5,12 | **,012** |
| Error | 37256,921 | 177 | 210,49 | | |

**Bonferroni test**

| Sig | 0 | 4 | 6 |
|---|---|---|---|
| **4** | **,097** | | |
| **6** | **,053** | **1,00** | |
| **12** | **,013** | **,956** | **1,00** |

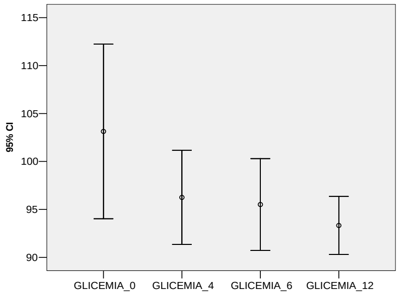


**Commento:** Come è evidente il trend del parametro nel tempo risulta significativamente discendente solo al 12mese vs 0.

**Descriptive Statistics**

| Factor1 | Mean | sd | N |
|---|---|---|---|
| HBA1C_0 | 39,18 | 10,42 | 60 |
| HBA1C_4 | 38,16 | 8,15 | 60 |
| HBA1C_6 | 37,23 | 5,20 | 60 |
| HBA1C_12 | 36,50 | 4,66 | 60 |

ANOVA per misure multiple

| | Sum of Squares | df | Mean² | F | Sig. |
|---|---|---|---|---|---|
| factor1 | 241,778 | 3 | 80,593 | 3,137 | **,057** |
| Error | 4547,825 | 177 | 25,694 | | |

**Bonferroni test**

| Sig | 0 | 4 | 6 |
|---|---|---|---|
| **4** | **1,00** | | |
| **6** | **,501** | **1,00** | |
| **12** | **,168** | **,435** | **,986** |

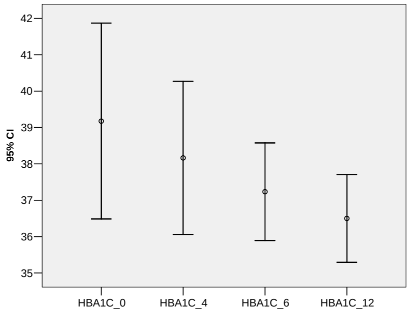


**Commento:** Come è evidente il trend del parametro nel tempo NON risulta significativo anche se risulta evidente un decremento del punto medio e della variabilità del parametro nel tempo di trattamento.

| Descriptive Statistics | | | |
|---|---|---|---|
| Factor1 | Mean | sd | N |
| COLTOT_0 | 191,70 | 36,00 | 60 |
| COLTOT_4 | 186,28 | 29,48 | 60 |
| COLTOT_6 | 185,12 | 32,95 | 60 |
| COLTOT_12 | 180,88 | 23,29 | 60 |

| ANOVA per misure multiple | | | | | |
|---|---|---|---|---|---|
| | SumofSquares | df | Mean $^2$ | F | Sig. |
| factor1 | 3571,846 | 3 | 1190,62 | 2,77 | **,043** |
| Error | 76082,404 | 177 | 429,84 | | |

| **Bonferroni test** | | | |
|---|---|---|---|
| Sig | **0** | **4** | **6** |
| **4** | **,802** | | |
| **6** | **,703** | **1,00** | |
| **12** | **,042** | **,637** | **1,00** |

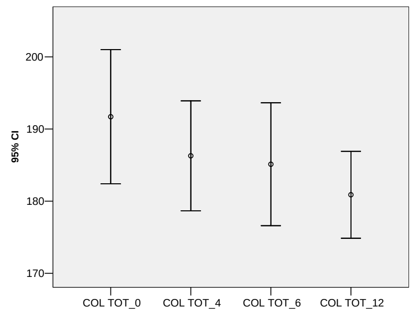


**Commento:** Come è evidente il trend del parametro nel tempo risulta significativamente discendente solo al 12mese vs 0.

| Descriptive Statistics | | | |
|---|---|---|---|
| Factor1 | Mean | sd | N |
| HLD_0 | 56,60 | 18,80 | 60 |
| HLD_4 | 54,13 | 17,44 | 60 |
| HLD_6 | 55,88 | 18,32 | 60 |
| HLD_12 | 53,90 | 17,38 | 60 |

| ANOVA per misure multiple | | | | | |
|---|---|---|---|---|---|
| | SumofSquares | df | Mean$^2$ | F | Sig. |
| factor1 | 314,478 | 3 | 104,826 | ,763 | **,516** |
| Error | 24304,702 | 177 | 137,315 | | |

| **Bonferroni test** | | | |
|---|---|---|---|
| Sig | **0** | **4** | **6** |
| **4** | **1,00** | | |
| **6** | **1,00** | **1,00** | |
| **12** | **,926** | **1,00** | **1,00** |

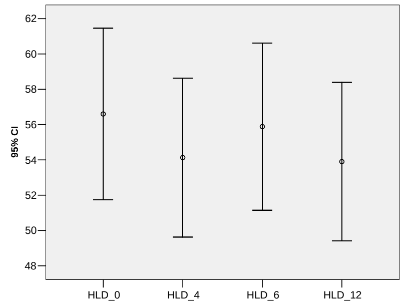


**Commento:** Come è evidente il trend del parametro nel tempo NON risulta significativo.

| Descriptive Statistics | | | |
|---|---|---|---|
| Factor1 | Mean | sd | N |
| LDL_0 | 113,10 | 32,00 | 60 |
| LDL_4 | 116,50 | 34,38 | 60 |
| LDL_6 | 116,22 | 32,20 | 60 |
| LDL_12 | 111,65 | 26,74 | 60 |

| ANOVA per misure multiple | | | | | |
|---|---|---|---|---|---|
| | SumofSquares | df | Mean$^2$ | F | Sig. |
| factor1 | 1017,500 | 3 | 339,167 | ,683 | **,563** |
| Error | 87878,000 | 177 | 496,486 | | |

| **Bonferroni test** | | | |
|---|---|---|---|
| Sig | **0** | **4** | **6** |
| **4** | **1,00** | | |
| **6** | **1,00** | **1,00** | |
| **12** | **1,00** | **1,00** | **1,00** |

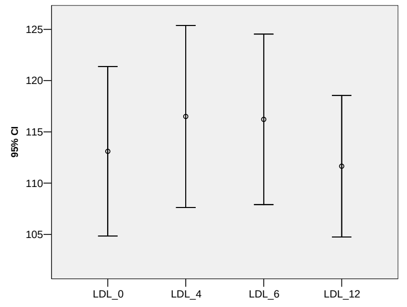


**Commento:** Come è evidente il trend del parametro nel tempo NON risulta significativamente diverso nel tempo del trattamento.

| Descriptive Statistics | | | | | ANOVA per misure multiple | | | | | | | Bonferroni test | | | |
|---|---|---|---|---|---|---|---|---|---|---|---|---|---|---|---|
| Factor1 | Mean | sd | N | | | SumofSquares | df | Mean$^2$ | F | Sig. | | Sig | 0 | 4 | 6 |
| **TRIGLICERIDI_0** | 114,37 | 77,98 | 60 | | factor1 | 12615,279 | 3 | 4205,1 | 2,37 | **,072** | | **4** | **1,00** | | |
| **TRIGLICERIDI_4** | 105,07 | 39,66 | 60 | | Error | 314477,971 | 177 | 1776,7 | | | | **6** | **,930** | **1,00** | |
| **TRIGLICERIDI_6** | 100,85 | 41,01 | 60 | | | | | | | | | **12** | **,236** | **,372** | **1,00** |
| **TRIGLICERIDI_12** | 94,40 | 32,20 | 60 | | | | | | | | | | | | |

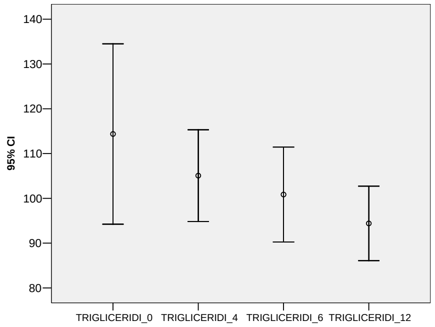


**Commento:** Come è evidente il trend del parametro nel tempo NON risulta significativo anche se si deve ammettere un decremento del punto medio.

| Descriptive Statistics | | | | | ANOVA per misure multiple | | | | | | | Bonferroni test | | | |
|---|---|---|---|---|---|---|---|---|---|---|---|---|---|---|---|
| Factor1 | Mean | sd | N | | | SumofSquares | df | Mean$^2$ | F | Sig. | | Sig | 0 | 4 | 6 |
| VES_0 | 15,28 | 13,22 | 60 | | factor1 | 611,946 | 3 | 203,982 | 1,931 | **,126** | | **4** | **1,00** | | |
| VES_4 | 13,85 | 16,34 | 60 | | Error | 18702,304 | 177 | 105,663 | | | | **6** | **,599** | **1,00** | |
| VES_6 | 12,82 | 10,20 | 60 | | | | | | | | | **12** | **,064** | **1,00** | **1,00** |
| VES_12 | 10,90 | 9,93 | 60 | | | | | | | | | | | | |

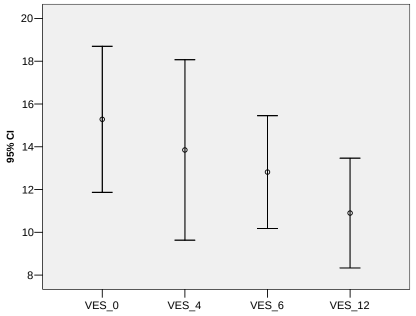


**Commento:** Come è evidente il trend del parametro nel tempo NON risulta significativo anche se si deve ammettere un decremento del punto medio.

| Descriptive Statistics | | | | | ANOVA per misure multiple | | | | | | | Bonferroni test | | | |
|---|---|---|---|---|---|---|---|---|---|---|---|---|---|---|---|
| Factor1 | Mean | sd | N | | | SumofSquares | df | Mean$^2$ | F | Sig. | | Sig | 0 | 4 | 6 |
| PCR_0 | 3,19 | 7,88 | 60 | | factor1 | 150,040 | 3 | 50,013 | 3,302 | ,022 | | **4** | **,598** | | |
| PCR_4 | 1,53 | 2,05 | 60 | | Error | 2680,621 | 177 | 15,145 | | | | **6** | **,694** | **1,00** | |
| PCR_6 | 1,63 | 2,60 | 60 | | | | | | | | | **12** | **,024** | **,832** | **,174** |
| PCR_12 | 1,10 | 1,84 | 60 | | | | | | | | | | | | |

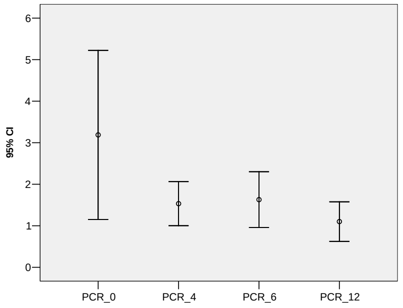


**Commento:** Come è evidente il trend del parametro nel tempo risulta significativo solo al 12mese vs 0.

| Descriptive Statistics | | | |
|---|---|---|---|
| Factor1 | Mean | sd | N |
| PASI_0 | 12,17 | 11,01 | 60 |
| PASI_4 | 2,97 | 3,68 | 60 |
| PASI_6 | 1,74 | 2,54 | 60 |
| PASI_12 | 1,57 | 2,46 | 60 |

| ANOVA per misure multiple | | | | | |
|---|---|---|---|---|---|
| | SumofSquares | df | $Mean^2$ | F | Sig. |
| factor1 | 4637,578 | 3 | 1545,859 | 53,752 | **,000** |
| Error | 5090,359 | 177 | 28,759 | | |

| **Bonferroni test** | | | |
|---|---|---|---|
| Sig | **0** | **4** | **6** |
| **4** | **000** | | |
| **6** | **000** | **,002** | |
| **12** | **,000** | **,041** | **1,00** |

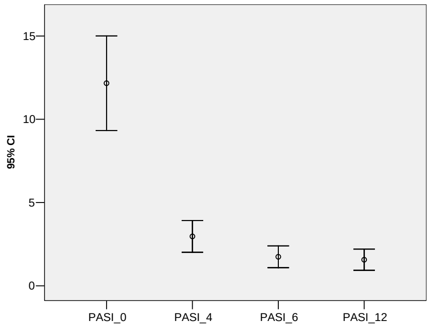


**Commento:** Come è evidente il trend del parametro nel tempo risulta significativamente discendente, già al 4° mese!

## CONFRONTI LONGITUDINALI (factor1=T0;T4;T6;T12 mesi) per interluchine

**Descriptive Statistics**

| Factor1 | Sex | Mean | sd | N |
|---|---|---|---|---|
| IL10_0 | 0=F | ,374 | ,203 | 19 |
| | 1=M | ,454 | ,246 | 37 |
| | total | ,426 | ,234 | 56 |
| IL10_4 | 0=F | ,347 | ,215 | 19 |
| | 1=M | ,397 | ,284 | 37 |
| | total | ,380 | ,262 | 56 |
| IL10_6 | 0=F | ,341 | ,206 | 19 |
| | 1=M | ,271 | ,218 | 37 |
| | total | ,295 | ,215 | 56 |
| IL10_12 | 0=F | ,354 | ,218 | 19 |
| | 1=M | ,231 | ,254 | 37 |
| | total | ,273 | ,248 | 56 |

ANOVA per misure multiple

| | F | df | Mean$^2$ | F | Sig. |
|---|---|---|---|---|---|
| factor1 | ,489 | 3 | ,163 | 4,431 | **,005** |
| factor1*SEX | ,350 | 3 | ,117 | 3,170 | **,056** |
| Error | 5,964 | 162 | ,037 | | |

**Bonferroni test**

| Sig | **0** | **4** | **6** |
|---|---|---|---|
| **4** | **1,00** | | |
| **6** | **,037** | **,238** | |
| **12** | **,042** | **,389** | **1,00** |

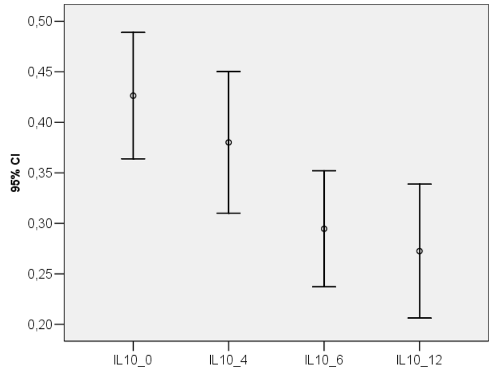


**Commento:** Come è evidente il trend del parametro nel tempo risulta significativamente discendente solo da 0 al 6°mese e da 0 al 12°mese!
Il sex si mantiene proporzionato e non interviene nella valutazione di factor1!

**Descriptive Statistics**

| Factor1 | Guarisce | Mean | sd | N |
|---|---|---|---|---|
| IL10_0 | 0=no | ,28 | ,30 | 5 |
| | 1=s1 | ,44 | ,23 | 51 |
| | total | ,43 | ,23 | 56 |
| IL10_4 | 0=no | ,15 | ,04 | 5 |
| | 1=s1 | ,40 | ,26 | 51 |
| | total | ,38 | ,26 | 56 |
| IL10_6 | 0=no | ,12 | ,05 | 5 |
| | 1=s1 | ,31 | ,22 | 51 |
| | total | ,29 | ,21 | 56 |
| IL10_12 | 0=no | ,08 | ,05 | 5 |
| | 1=s1 | ,29 | ,25 | 51 |
| | total | ,27 | ,25 | 56 |

ANOVA per misure multiple

| | F | df | Mean$^2$ | F | Sig. |
|---|---|---|---|---|---|
| factor1 | ,333 | 3 | ,111 | 2,859 | **,051** |
| factor1*guarisce | ,022 | 3 | ,007 | ,188 | **,905** |
| Error | 6,293 | 162 | ,039 | | |

**Commento:** Come è evidente il trend del parametro nel tempo NON risulta significativo. Il sex si mantiene proporzionato e non interviene nella valutazione di factor1!

**Bonferroni test**

| Sig | **0** | **4** | **6** |
|---|---|---|---|
| **4** | **1,00** | | |
| **6** | **,169** | **1,00** | |
| **12** | **,176** | **1,00** | **1,00** |

**Descriptive Statistics**

| Factor1 | Neoplasia | Mean | sd | N |
|---|---|---|---|---|
| IL10_0 | 0=no | ,44 | ,23 | 43 |
| | 1=s1 | ,39 | ,26 | 13 |
| | total | ,43 | ,23 | 56 |
| IL10_4 | 0=no | ,40 | ,27 | 43 |
| | 1=s1 | ,31 | ,23 | 13 |
| | total | ,38 | ,26 | 56 |
| IL10_6 | 0=no | ,29 | ,22 | 43 |
| | 1=s1 | ,30 | ,21 | 13 |
| | total | ,29 | ,21 | 56 |
| IL10_12 | 0=no | ,27 | ,26 | 43 |
| | 1=s1 | ,27 | ,22 | 13 |
| | total | ,27 | ,25 | 56 |

ANOVA per misure multiple

| | F | df | Mean$^2$ | F | Sig. |
|---|---|---|---|---|---|
| factor1 | ,484 | 3 | ,161 | 4,17 | **,054** |
| factor1*Neoplasia | ,060 | 3 | ,020 | ,521 | ,668 |
| Error | 6,25 | 162 | ,039 | | |

**Commento:** Come è evidente il trend del parametro nel tempo NON risulta significativo. Il sex si mantiene proporzionato e non interviene nella valutazione di factor1!

**Bonferroni test**

| Sig | **0** | **4** | **6** |
|---|---|---|---|
| **4** | **1,00** | | |
| **6** | **,062** | **,637** | |
| **12** | **,061** | **,530** | **1,00** |

| Descriptive Statistics | | | | |
|---|---|---|---|---|
| Factor1 | Sex | Mean | sd | N |
| TGF_0 | 0=F | 61,6 | 37,2 | 19 |
| | 1=M | 68,4 | 27,5 | 37 |
| | total | 66,1 | 31,0 | 56 |
| TGF_4 | 0=F | 59,8 | 25,4 | 19 |
| | 1=M | 68,1 | 30,8 | 37 |
| | total | 65,3 | 29,1 | 56 |
| TGF_6 | 0=F | 49,6 | 18,9 | 19 |
| | 1=M | 52,5 | 26,6 | 37 |
| | total | 51,5 | 24,1 | 56 |
| TGF_12 | 0=F | 53,8 | 17,6 | 19 |
| | 1=M | 46,1 | 20,4 | 37 |
| | total | 48,7 | 19,7 | 56 |

| ANOVA per misure multiple | | | | | |
|---|---|---|---|---|---|
| | F | df | Mean$^2$ | F | Sig. |
| factor1 | 9833,02 | 3 | 3277,68 | 6,597 | ,000 |
| factor1*SEX | 1943,05 | 3 | 647,68 | 1,304 | ,275 |
| Error | 80489,51 | 162 | 496,85 | | |

| Bonferroni test | | | |
|---|---|---|---|
| Sig | 0 | 4 | 6 |
| 4 | 1,00 | | |
| 6 | ,084 | ,002 | |
| 12 | ,029 | ,004 | 1,00 |

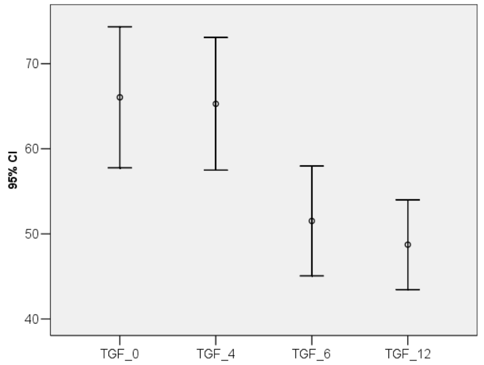


**Commento:** Come è evidente il trend del parametro nel tempo risulta significativamente discendente solo da 0 al 12°mese e da 4 al 6° e 12°mese! Il sex si mantiene proporzionato e non interviene nella valutazione di factor1!

| Descriptive Statistics | | | | |
|---|---|---|---|---|
| Factor1 | Guarisce | Mean | sd | N |
| TGF_0 | 0=no | 59,7 | 49,6 | 5 |
| | 1=s1 | 66,8 | 29,2 | 51 |
| | total | 66,1 | 31,0 | 56 |
| TGF_4 | 0=no | 57,1 | 37,2 | 5 |
| | 1=s1 | 66,1 | 28,5 | 51 |
| | total | 65,3 | 29,1 | 56 |
| TGF_6 | 0=no | 38,0 | 16,0 | 5 |
| | 1=s1 | 52,8 | 24,5 | 51 |
| | total | 51,5 | 24,1 | 56 |
| TGF_12 | 0=no | 28,4 | 19,9 | 5 |
| | 1=s1 | 50,7 | 19,5 | 51 |
| | total | 48,7 | 19,7 | 56 |

| ANOVA per misure multiple | | | | | |
|---|---|---|---|---|---|
| | F | df | Mean$^2$ | F | Sig. |
| factor1 | 6909,9 | 1 | 6909,9 | 9,8 | ,003 |
| factor1*guarisce | 617,47 | 1 | 617,47 | ,88 | ,353 |
| Error | 37954,9 | 54 | 702,87 | | |

**Commento:** Come è evidente il trend del parametro nel tempo risulta significativamente discendente solo da 0 al 12°mese e dal 4° al 6° e 12°mese! Il sex si mantiene proporzionato e non interviene nella valutazione di factor1!

| Bonferroni test | | | |
|---|---|---|---|
| Sig | 0 | 4 | 6 |
| 4 | 1,00 | | |
| 6 | ,334 | ,036 | |
| 12 | ,048 | ,009 | 1,00 |

| Descriptive Statistics | | | | |
|---|---|---|---|---|
| Factor1 | Neoplas | Mean | sd | N |
| TGF_0 | 0=no | 65,9 | 30,1 | 43 |
| | 1=s1 | 66,6 | 35,0 | 13 |
| | total | 66,1 | 31,0 | 56 |
| TGF_4 | 0=no | 69,9 | 29,4 | 43 |
| | 1=s1 | 50,0 | 23,0 | 13 |
| | total | 65,3 | 29,1 | 56 |
| TGF_6 | 0=no | 52,6 | 25,4 | 43 |
| | 1=s1 | 47,8 | 19,9 | 13 |
| | total | 51,5 | 24,1 | 56 |
| TGF_12 | 0=no | 49,4 | 19,9 | 43 |
| | 1=s1 | 46,4 | 19,7 | 13 |
| | total | 48,7 | 19,7 | 56 |

| ANOVA per misure multiple | | | | | |
|---|---|---|---|---|---|
| | F | df | Mean$^2$ | F | Sig. |
| factor1 | 8746,5 | 3 | 2915,5 | 5,9 | ,001 |
| factor1*Neoplas | 2475,2 | 3 | 825,1 | 1,7 | ,175 |
| Error | 79957,3 | 162 | 493,6 | | |

**Commento:** Come è evidente il trend del parametro nel tempo risulta significativamente discendente solo da 0 al 12°mese e dal 4° al 12°mese! Il sex si mantiene proporzionato e non interviene nella valutazione di factor1!

| Bonferroni test | | | |
|---|---|---|---|
| Sig | 0 | 4 | 6 |
| 4 | 1,00 | | |
| 6 | ,070 | ,066 | |
| 12 | ,016 | ,048 | 1,00 |

**Descriptive Statistics**

| Factor1 | Sex | Mean | sd | N |
|---|---|---|---|---|
| IL17_0 | 0=F | ,131 | ,057 | 20 |
| | 1=M | ,128 | ,055 | 37 |
| | total | ,129 | ,055 | 57 |
| IL17_4 | 0=F | ,074 | ,046 | 20 |
| | 1=M | ,088 | ,042 | 37 |
| | total | ,083 | ,043 | 57 |
| IL17_6 | 0=F | ,058 | ,047 | 20 |
| | 1=M | ,069 | ,043 | 37 |
| | total | ,065 | ,044 | 57 |
| IL17_12 | 0=F | ,026 | ,033 | 20 |
| | 1=M | ,018 | ,027 | 37 |
| | total | ,021 | ,029 | 57 |

ANOVA per misure multiple

| | F | df | Mean$^2$ | F | Sig. |
|---|---|---|---|---|---|
| factor1 | ,308 | 3 | ,103 | 74,981 | **,000** |
| factor1*SEX | ,004 | 3 | ,001 | 1,072 | ,362 |
| Error | ,226 | 165 | ,001 | | |

**Bonferroni test**

| Sig | 0 | 4 | 6 |
|---|---|---|---|
| **4** | **,000** | | |
| **6** | **,000** | **,004** | |
| **12** | **,000** | **,000** | **,000** |

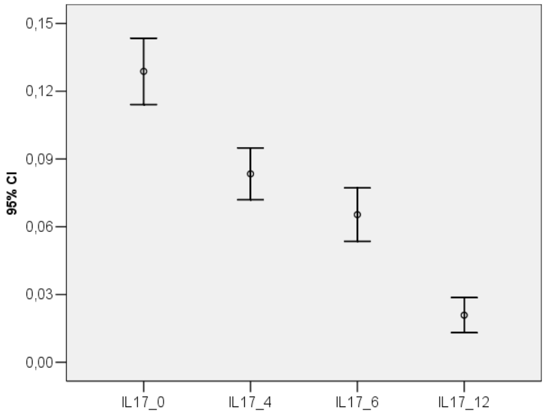


**Commento:** Come è evidente il trend del parametro nel tempo risulta significativamente discendente per tutti i mesi! Il sex si mantiene proporzionato e non interviene nella valutazione di factor1!

**Descriptive Statistics**

| Factor1 | Guarisce | Mean | sd | N |
|---|---|---|---|---|
| IL17_0 | 0=no | ,110 | ,055 | 5 |
| | 1=s1 | ,131 | ,056 | 52 |
| | total | ,129 | ,055 | 57 |
| IL17_4 | 0=no | ,097 | ,048 | 5 |
| | 1=s1 | ,082 | ,043 | 52 |
| | total | ,083 | ,043 | 57 |
| IL17_6 | 0=no | ,093 | ,048 | 5 |
| | 1=s1 | ,063 | ,044 | 52 |
| | total | ,065 | ,044 | 57 |
| IL17_12 | 0=no | ,054 | ,065 | 5 |
| | 1=s1 | ,018 | ,022 | 52 |
| | total | ,021 | ,029 | 57 |

ANOVA per misure multiple

| | F | df | Mean$^2$ | F | Sig. |
|---|---|---|---|---|---|
| factor1 | ,067 | 3 | ,022 | 16,613 | **,000** |
| factor1*guarisce | ,009 | 3 | ,003 | 2,259 | ,083 |
| Error | ,221 | 165 | ,001 | | |

**Commento:** Come è evidente il trend del parametro nel tempo risulta significativo da 0 a 6 e 12mese da 4° a 12°mese e da 6° a 12°mese. Il sex si mantiene proporzionato e non interviene nella valutazione di factor1!

**Bonferroni test**

| Sig | 0 | 4 | 6 |
|---|---|---|---|
| **4** | **,173** | | |
| **6** | **,040** | **,974** | |
| **12** | **,000** | **,000** | **,001** |

**Descriptive Statistics**

| Factor1 | Neoplas | Mean | sd | N |
|---|---|---|---|---|
| IL17_0 | 0=no | ,129 | ,057 | 44 |
| | 1=s1 | ,128 | ,052 | 13 |
| | total | ,129 | ,055 | 57 |
| IL17_4 | 0=no | ,082 | ,044 | 44 |
| | 1=s1 | ,087 | ,043 | 13 |
| | total | ,083 | ,043 | 57 |
| IL17_6 | 0=no | ,064 | ,043 | 44 |
| | 1=s1 | ,071 | ,051 | 13 |
| | total | ,065 | ,044 | 57 |
| IL17_12 | 0=no | ,013 | ,016 | 44 |
| | 1=s1 | ,046 | ,047 | 13 |
| | total | ,021 | ,029 | 57 |

ANOVA per misure multiple

| | F | df | Mean$^2$ | F | Sig. |
|---|---|---|---|---|---|
| factor1 | ,202 | 3 | ,067 | 49,805 | **,000** |
| factor1*Neoplas | ,007 | 3 | ,002 | 1,640 | ,182 |
| Error | ,224 | 165 | ,001 | | |

**Commento:** Come è evidente il trend del parametro nel tempo risulta significativamente discendente per tutti i mesi! Il sex si mantiene proporzionato e non interviene nella valutazione di factor1!

**Bonferroni test**

| Sig | 0 | 4 | 6 |
|---|---|---|---|
| **4** | **,000** | | |
| **6** | **,000** | **,017** | |
| **12** | **,000** | **,000** | **,000** |

## DESCRITTIVA  Campione con drop-out

## COMPOSIZIONE CAMPIONE DI PARTENZA T0 (N°=68; 29F;39M)

| Descrizione | | | | | | | Anova oneway | | | | | |
|---|---|---|---|---|---|---|---|---|---|---|---|---|
| | | | | | | | | | | | | |
| | sexM1 | N | Mean | SD | Min. | Max. | | Sum of sq | df | Mean$^2$ | F | Sig. |
| AGE | F=0 | 29 | 51,3 | 13,9 | 25 | 79 | Between | 14,887 | 1 | 14,887 | ,074 | **,787** |
| | M=1 | 39 | 52,3 | 14,5 | 25 | 78 | Within | 13359,643 | 66 | 202,419 | | |
| | total | 68 | 51,9 | 14,1 | 25 | 79 | Total | 13374,529 | 67 | | | |
| PESO | F=0 | 29 | 69,62 | 12,2 | 50 | 114 | Between | 1800,669 | 1 | 1800,669 | 32,741 | **,000** |
| | M=1 | 39 | 81,77 | 14,1 | 56 | 130 | Within | 3629,802 | 66 | 54,997 | | |
| | total | 68 | 76,59 | 14,6 | 50 | 130 | Total | 5430,471 | 67 | | | |
| ALTEZZA | F=0 | 29 | 166,62 | 7,3 | 150 | 181 | Between | 10,169 | 1 | 10,169 | ,458 | **,501** |
| | M=1 | 39 | 177,03 | 7,5 | 160 | 195 | Within | 1464,977 | 66 | 22,197 | | |
| | total | 68 | 172,59 | 9,0 | 150 | 195 | Total | 1475,146 | 67 | | | |
| BMI | F=0 | 29 | 25,29 | 5,55 | 16,71 | 46,25 | Between | 2454,720 | 1 | 2454,720 | 13,786 | **,000** |
| | M=1 | 39 | 26,07 | 3,98 | 17,90 | 37,18 | Within | 11751,751 | 66 | 178,057 | | |
| | total | 68 | 25,73 | 4,69 | 16,71 | 46,25 | Total | 14206,471 | 67 | | | |

**Commento:** Come atteso tra gruppi di genere i maschi sono statisticamente più pesanti e più alti, tuttavia l'età e il bmi risultano del tutto omogenei.

**CuraInf1S * Infezione1S Crosstabulation**

| | | Infezione1S | | | Chi-SquareTests | | | |
|---|---|---|---|---|---|---|---|---|
| Cura1nf1s | | 0=No | 1=Si | Total | | Value | df | Sig. |
| 0=No | Count | 36 | 13 | 49 | Chi-Square | 29,663 | 1 | **,000** |
| | % curaInf1S | 73,5% | 26,5% | 100,0% | N | 68 | | |
| | % Infezione1S | 100,0% | 40,6% | 72,1% | | | | |
| 1=SI | Count | 0 | 19 | 19 | Commento: il test certifica ovviamente che le occorrenze degli infetti curati sono statisticamente più alte dei non infetti! | | | |
| | % curaInf1S | ,0% | 100,0% | 100,0% | | | | |
| | % Infezione1S | ,0% | 59,4% | 27,9% | | | | |
| Total | Count | 36 | 32 | 68 | | | | |
| | % curaInf1S | 52,9% | 47,1% | 100,0% | | | | |
| | % Infezione1S | 100,0% | 100,0% | 100,0% | | | | |

**CuraInf1S * TipoInf  Crosstabulation**

| | | TipoInf | | | | Chi-SquareTests | | | |
|---|---|---|---|---|---|---|---|---|---|
| Cura1nf1s | | 1=alb | 2=alb+noalb | 3=noalb | Total | | Value | df | Sig. |
| 0=No | Count | 2 | 10 | 1 | 13 | Chi-Square | 24,498 | 2 | **,000** |
| | % curaInf1S | 15,4% | 76,9% | 7,7% | 100,0% | N | 32 | | |
| | % TipoInf | 9,5% | 100,0% | 100,0% | 40,6% | | | | |
| 1=SI | Count | 19 | 0 | 0 | 19 | Commento: il test certifica statisticamente che le occorrenze dei curati sono solo del tipo albicans. | | | |
| | % curaInf1S | 100,0% | ,0% | ,0% | 100,0% | | | | |
| | % TipoInf | 90,5% | ,0% | ,0% | 59,4% | | | | |
| Total | Count | 21 | 10 | 1 | 32 | | | | |
| | % curaInf1S | 65,6% | 31,3% | 3,1% | 100,0% | | | | |
| | % TipoInf | 100,0% | 100,0% | 100,0% | 100,0% | | | | |

**CuraInf1S * SEX1M Crosstabulation**

| | | SEX1M | | | Chi-SquareTests | | | |
|---|---|---|---|---|---|---|---|---|
| Cura1nf1s | | 0=F | 1=M | Total | | Value | df | Sig. |
| 0=No | Count | 7 | 6 | 13 | Chi-Square | ,051 | 1 | ,821 |
| | % curaInf1S | 53,8% | 46,2% | 100,0% | N | 32 | | |
| | % SEX1M | 38,9% | 42,9% | 40,6% | | | | |
| 1=SI | Count | 11 | 8 | 19 | Commento: il test certifica che le cure sono equamente distribuite tra i generi. | | | |
| | % curaInf1S | 57,9% | 42,1% | 100,0% | | | | |
| | % SEX1M | 61,1% | 57,1% | 59,4% | | | | |
| Total | Count | 18 | 14 | 32 | | | | |
| | % curaInf1S | 56,3% | 43,8% | 100,0% | | | | |
| | % SEX1M | 100,0% | 100,0% | 100,0% | | | | |

**Infezione1S * SEX1M Crosstabulation**

| Infezione1S | | SEX1M 0=F | SEX1M 1=M | Total |
|---|---|---|---|---|
| 0=No | Count | 11 | 25 | 36 |
| | % Infezione1S | 30,6% | 69,4% | 100,0% |
| | % SEX1M | 37,9% | 64,1% | 52,9% |
| 1=SI | Count | 18 | 14 | 32 |
| | % Infezione1S | 56,3% | 43,8% | 100,0% |
| | % SEX1M | 62,1% | 35,9% | 47,1% |
| Total | Count | 29 | 39 | 68 |
| | % Infezione1S | 42,6% | 57,4% | 100,0% |
| | % SEX1M | 100,0% | 100,0% | 100,0% |

Chi-SquareTests

| | Value | df | Sig. |
|---|---|---|---|
| Chi-Square | 4,573 | 1 | **,032** |
| N | 68 | | |

Commento: il test certifica che le Femmine sono statisticamente più infette rispetto ai Maschi.

**Descriptive Statistics** Dependent Variable: AGE

| SEX1M | Infezione1S | Mean | sd | N |
|---|---|---|---|---|
| 0=F | 0=No | 45,8 | 13,1 | 11 |
| | 1=Si | 54,7 | 13,6 | 18 |
| | Total | 51,3 | 13,8 | 29 |
| 1=M | 0=No | 50,3 | 13,9 | 25 |
| | 1=Si | 55,8 | 15,3 | 14 |
| | Total | 52,3 | 14,5 | 39 |
| Total | 0=No | 48,9 | 13,6 | 36 |
| | 1=Si | 55,2 | 14,2 | 32 |
| | Total | 51,9 | 14,1 | 68 |

Anova di AGE a 2 fattori

| Source | Sum of Squares | df | Mean Square | F | sig |
|---|---|---|---|---|---|
| **SEX1M** | 120,771 | 1 | 120,771 | ,616 | ,436 |
| **Infezione1S** | 798,947 | 1 | 798,947 | 4,073 | **,048** |
| **SEX1M * Infezione1S** | 43,328 | 1 | 43,328 | ,221 | ,640 |
| **Error** | 12553,034 | 64 | 196,141 | | |
| **Corrected Total** | 13374,529 | 67 | | | |

Commento: Anova conferma una significativa influenza sulla età delle infezioni per le Femmine rispetto ai Maschi.

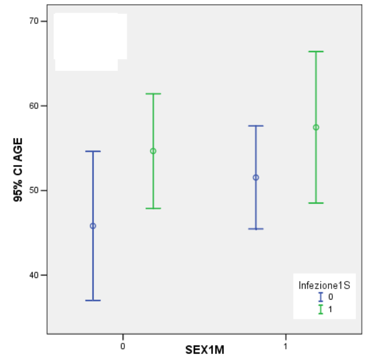


E’ evidente l’effetto statistico (anche se al limite!) sull’età degli infetti nel campione Femmine e non nel campione Maschi.

**Infezione1S * NEOPLASIA Crosstabulation**

| Infezione1S | | NEOPLASIA 0=No | NEOPLASIA 1=Si | Total |
|---|---|---|---|---|
| 0=No | Count | 32 | 4 | 36 |
| | % Infezione1S | 88,9% | 11,1% | 100,0% |
| | % NEOPLASIA | 58,2% | 30,8% | 52,9% |
| 1=SI | Count | 23 | 9 | 32 |
| | % Infezione1S | 71,9% | 28,1% | 100,0% |
| | % NEOPLASIA | 41,8% | 69,2% | 47,1% |
| Total | Count | 55 | 13 | 68 |
| | % Infezione1S | 80,9% | 19,1% | 100,0% |
| | % NEOPLASIA | 100,0% | 100,0% | 100,0% |

Chi-SquareTests

| | Value | df | Sig. |
|---|---|---|---|
| Chi-Square | 3,171 | 1 | ,075 |
| N | 68 | | |

Commento: il test certifica che le Infezioni pur se più alte nei casi di Neoplasie non sono statisticamente diverse.

**SEX1M * NEOPLASIA Crosstabulation**

| SEX1M | | NEOPLASIA 0=No | NEOPLASIA 1=Si | Total |
|---|---|---|---|---|
| 0=F | Count | 22 | 7 | 29 |
| | % SEX | 75,9% | 24,1% | 100,0% |
| | % NEOPLASIA | 40,0% | 53,8% | 42,6% |
| 1=M | Count | 33 | 6 | 39 |
| | % SEX | 84,6% | 15,4% | 100,0% |
| | % NEOPLASIA | 60,0% | 46,2% | 57,4% |
| Total | Count | 55 | 13 | 68 |
| | % SEX | 80,9% | 19,1% | 100,0% |
| | % NEOPLASIA | 100,0% | 100,0% | 100,0% |

Chi-SquareTests

| | Value | df | Sig. |
|---|---|---|---|
| Chi-Square | ,824 | 1 | ,364 |
| N | 68 | | |

Commento: il test certifica che le Neuplasie pur più frequenti nelle Femmine non sono statisticamente diverse da quelle dei Maschi.

**Descriptive Statistics** Dependent Variable: BMI

| SEX1M | Infezione1S | Mean | sd | N |
|---|---|---|---|---|
| 0=F | 0=No | 22,64 | 2,32 | 11 |
| | 1=Si | 26,90 | 6,35 | 18 |
| | Total | 25,29 | 5,55 | 29 |
| 1=M | 0=No | 26,76 | 4,45 | 25 |
| | 1=Si | 24,82 | 2,66 | 14 |
| | Total | 26,07 | 3,98 | 39 |
| Total | 0=No | 25,50 | 4,34 | 36 |
| | 1=Si | 25,99 | 5,12 | 32 |
| | Total | 25,73 | 4,69 | 68 |

Anova di BMI a 2 fattori

| Source | Sum of Squares | df | Mean Square | F | sig |
|---|---|---|---|---|---|
| **SEX1M** | 16,238 | 1 | 16,238 | ,795 | ,376 |
| **Infezione1S** | 20,983 | 1 | 20,983 | 1,028 | ,315 |
| **SEX1M * Infezione1S** | 149,527 | 1 | 149,527 | 7,323 | **,009** |
| **Error** | 1306,766 | 64 | 20,418 | | |
| **Corrected Total** | 1475,146 | 67 | | | |

Commento: Anova conferma una significativa influenza sul BMI delle infezioni diversa tra Femmine e Maschi.

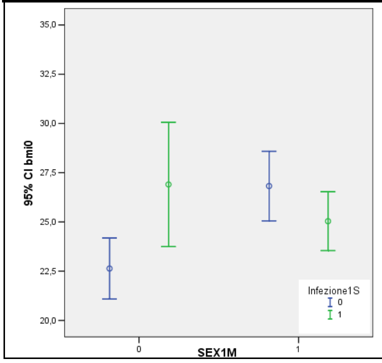


E' evidente l'effetto statistico diverso delle infezioni sul BMI del campione Femmine rispetto al campione Maschi.

**SEX1M * FUMO Crosstabulation**

| SEX1M | | FUMO 0=No | FUMO 1=Si | Total |
|---|---|---|---|---|
| 0=F | Count | 19 | 10 | 29 |
| | % SEX | 65,5% | 34,5% | 100,0% |
| | % FUMO | 51,4% | 32,3% | 42,6% |
| 1=M | Count | 18 | 21 | 39 |
| | % SEX | 46,2% | 53,8% | 100,0% |
| | % FUMO | 48,6% | 67,7% | 57,4% |
| Total | Count | 37 | 31 | 68 |
| | % SEX | 54,4% | 45,6% | 100,0% |
| | % FUMO | 100,0% | 100,0% | 100,0% |

Chi-SquareTests

| | Value | df | Sig. |
|---|---|---|---|
| Chi-Square | 2,514 | 1 | **,113** |
| N | 68 | | |

Commento: il test certifica che il FUMO pur più frequente nei Maschi non sono statisticamente diverse da quelle delle Femmine.

**SEX1M * ALCOL Crosstabulation**

| SEX1M | | ALCOL 0=No | ALCOL 1=Si | Total |
|---|---|---|---|---|
| 0=F | Count | 23 | 6 | 29 |
| | % SEX | 79,3% | 20,7% | 100,0% |
| | % ALCOL | 43,4% | 40,0% | 42,6% |
| 1=M | Count | 30 | 9 | 39 |
| | % SEX | 76,9% | 23,1% | 100,0% |
| | % ALCOL | 56,6% | 60,0% | 57,4% |
| Total | Count | 53 | 15 | 68 |
| | % SEX | 77,9% | 22,1% | 100,0% |
| | % ALCOL | 100,0% | 100,0% | 100,0% |

Chi-SquareTests

| | Value | df | Sig. |
|---|---|---|---|
| Chi-Square | ,055 | 1 | ,814 |
| N | 68 | | |

Commento: il test certifica che l'ALCOL pur più frequente nei Maschi non sono statisticamente diverse da quelle delle Femmine.

**Infezione1S * ALCOL Crosstabulation**

| Infezione1S | | ALCOL 0=No | ALCOL 1=Si | Total |
|---|---|---|---|---|
| 0=No | Count | 28 | 8 | 36 |
| | % Infezione1S | 77,8% | 22,2% | 100,0% |
| | % ALCOL | 52,8% | 53,3% | 52,9% |
| 1=SI | Count | 25 | 7 | 32 |
| | % Infezione1S | 78,1% | 21,9% | 100,0% |
| | % ALCOL | 47,2% | 46,7% | 47,1% |
| Total | Count | 53 | 15 | 68 |
| | % Infezione1S | 77,9% | 22,1% | 100,0% |
| | % ALCOL | 100,0% | 100,0% | 100,0% |

Chi-SquareTests

| | Value | df | Sig. |
|---|---|---|---|
| Chi-Square | ,001 | 1 | ,973 |
| N | 68 | | |

Commento: il test certifica che le Infezioni pur se più alte nei casi di ALCCOL non sono statisticamente diverse per i non infetti.

**SEX1M * COMORBIDITA' Crosstabulation**

| | | COMORBIDITA' | | |
|---|---|---|---|---|
| SEX1M | | 0=No | 1=Si | Total |
| 0=F | Count | 10 | 19 | 29 |
| | % SEX | 34,5% | 65,5% | 100,0% |
| | %COMORBIDITA' | 43,5% | 42,2% | 42,6% |
| 1=M | Count | 13 | 26 | 39 |
| | % SEX | 33,3% | 66,7% | 100,0% |
| | %COMORBIDITA' | 56,5% | 57,8% | 57,4% |
| Total | Count | 23 | 45 | 68 |
| | % SEX | 33,8% | 66,2% | 100,0% |
| | %COMORBIDITA' | 100,0% | 100,0% | 100,0% |

| Chi-SquareTests | Value | df | Sig. |
|---|---|---|---|
| Chi-Square | ,010 | 1 | ,921 |
| N | 68 | | |

Commento: il test certifica che lle COMORBIDITA' sono equamente presenti nei 2 generi

**Infezione1S * COMORBIDITA' Crosstabulation**

| | | COMORBIDITA' | | |
|---|---|---|---|---|
| Infezione1S | | 0=No | 1=Si | Total |
| 0=No | Count | 17 | 19 | 36 |
| | % Infezione1S | 47,2% | 52,8% | 100,0% |
| | %COMORBIDITA' | 73,9% | 42,2% | 52,9% |
| 1=SI | Count | 6 | 26 | 32 |
| | % Infezione1S | 18,8% | 81,3% | 100,0% |
| | %COMORBIDITA' | 26,1% | 57,8% | 47,1% |
| Total | Count | 23 | 45 | 68 |
| | % Infezione1S | 33,8% | 66,2% | 100,0% |
| | %COMORBIDITA' | 100,0% | 100,0% | 100,0% |

| Chi-SquareTests | Value | df | Sig. |
|---|---|---|---|
| Chi-Square | 6,136 | 1 | **,013** |
| N | 68 | | |

Commento: il test certifica che le Infezioni sono statisticamente più alte per chi presenta COMORBIDITA'.

**Infezione1S * COMORBIDITA' CARDIOVASCOLARE Crosstabulation**

| | | COMORBIDITA'CARDIOVASCOLARE | | |
|---|---|---|---|---|
| Infezione1S | | 0=No | 1=Si | Total |
| 0=No | Count | 26 | 10 | 36 |
| | % Infezione1S | 72,2% | 27,8% | 100,0% |
| | %COMORBIDITA'CARDiO | 61,9% | 38,5% | 52,9% |
| 1=SI | Count | 16 | 16 | 32 |
| | % Infezione1S | 50,0% | 50,0% | 100,0% |
| | %COMORBIDITA'CARDiO | 38,1% | 61,5% | 47,1% |
| Total | Count | 42 | 26 | 68 |
| | % Infezione1S | 61,8% | 38,2% | 100,0% |
| | %COMORBIDITA'CARDiO | 100,0% | 100,0% | 100,0% |

| Chi-SquareTests | Value | df | Sig. |
|---|---|---|---|
| Chi-Square | 3,543 | 1 | ,060 |
| N | 68 | | |

Commento: il test certifica che le Infezioni pur se più alte nei casi di COMORBIDITA'CARDIOVASCOLARE non sono statisticamente diverse dalle non infezioni.

**Infezione1S * COMORBIDITA' METABOLICA Crosstabulation**

| | | COMORBIDITA' METABOLICA | | |
|---|---|---|---|---|
| Infezione1S | | 0=No | 1=Si | Total |
| 0=No | Count | 28 | 8 | 36 |
| | % Infezione1S | 77,8% | 22,2% | 100,0% |
| | %COMORBIDITA'METAB | 52,8% | 53,3% | 52,9% |
| 1=SI | Count | 25 | 7 | 32 |
| | % Infezione1S | 78,1% | 21,9% | 100,0% |
| | %COMORBIDITA'CARDiO | 47,2% | 46,7% | 47,1% |
| Total | Count | 53 | 15 | 68 |
| | % Infezione1S | 77,9% | 22,1% | 100,0% |
| | %COMORBIDITA'CARDiO | 100,0% | 100,0% | 100,0% |

| Chi-SquareTests | Value | df | Sig. |
|---|---|---|---|
| Chi-Square | ,001 | 1 | ,973 |
| N | 68 | | |

Commento: il test certifica che le Infezioni sono equamente presenti negli infetti e non infetti.

**Infezione1S * COMORBIDITA' INFETTIVE Crosstabulation**

| | | COMORBIDITA' INFETTIVE | | |
|---|---|---|---|---|
| Infezione1S | | 0=No | 1=Si | Total |
| 0=No | Count | 33 | 3 | 36 |
| | % Infezione1S | 91,7% | 8,3% | 100,0% |
| | %COMORBIDITA' INFETT | 55,0% | 37,5% | 52,9% |
| 1=SI | Count | 27 | 5 | 32 |
| | % Infezione1S | 84,4% | 15,6% | 100,0% |
| | %COMORBIDITA' INFETT | 45,0% | 62,5% | 47,1% |
| Total | Count | 60 | 8 | 68 |
| | % Infezione1S | 88,2% | 11,8% | 100,0% |
| | %COMORBIDITA' INFETT | 100,0% | 100,0% | 100,0% |

| Chi-SquareTests | Value | df | Sig. |
|---|---|---|---|
| Chi-Square | ,868 | 1 | ,352 |
| N | 68 | | |

Commento: il test certifica che le Infezioni pur se più alte nei casi di COMORBIDITA'INFETT non sono statisticamente diverse dai non infetti.

**Infezione1S * IPERCOLESTEROLEMIA Crosstabulation**

| | | IPERCOLESTEROLEMIA | | |
|---|---|---|---|---|
| Infezione1S | | 0=No | 1=Si | Total |
| 0=No | Count | 30 | 6 | 36 |
| | % Infezione1S | 83,3% | 16,7% | 100,0% |
| | % IPERCOLESTEROLEMIA | 52,6% | 54,5% | 52,9% |
| 1=SI | Count | 27 | 5 | 32 |
| | % Infezione1S | 84,4% | 15,6% | 100,0% |
| | % IPERCOLESTEROLEMIA | 47,4% | 45,5% | 47,1% |
| Total | Count | 57 | 11 | 68 |
| | % Infezione1S | 83,8% | 16,2% | 100,0% |
| | % IPERCOLESTEROLEMIA | 100,0% | 100,0% | 100,0% |

| Chi-SquareTests | Value | df | Sig. |
|---|---|---|---|
| Chi-Square | ,001 | 1 | ,973 |
| N | 68 | | |

Commento: il test certifica che le ipercolesterolemie sono equamente presenti negli infetti e non infetti.

**Infezione1S * DIABETE Crosstabulation**

| | | DIABETE | | |
|---|---|---|---|---|
| Infezione1S | | 0=No | 1=Si | Total |
| 0=No | Count | 32 | 4 | 36 |
| | % Infezione1S | 88,9% | 11,1% | 100,0% |
| | % DIABETE | 52,5% | 57,1% | 52,9% |
| 1=SI | Count | 29 | 3 | 32 |
| | % Infezione1S | 90,6% | 9,4% | 100,0% |
| | % DIABETE | 47,5% | 42,9% | 47,1% |
| Total | Count | 61 | 7 | 68 |
| | % Infezione1S | 89,7% | 10,3% | 100,0% |
| | % DIABETE | 100,0% | 100,0% | 100,0% |

| Chi-SquareTests | Value | df | Sig. |
|---|---|---|---|
| Chi-Square | ,055 | 1 | ,814 |
| N | 68 | | |

Commento: il test certifica che il DIABETE è equamente presente tra gli infetti e non infetti.

**Infezione1S * IPERTENSIONE Crosstabulation**

| | | IPERTENSIONE | | |
|---|---|---|---|---|
| Infezione1S | | 0=No | 1=Si | Total |
| 0=No | Count | 26 | 10 | 36 |
| | % Infezione1S | 72,2% | 27,8% | 100,0% |
| | % IPERTENSIONE | 59,1% | 41,7% | 52,9% |
| 1=SI | Count | 18 | 14 | 32 |
| | % Infezione1S | 56,3% | 43,8% | 100,0% |
| | % IPERTENSIONE | 40,9% | 58,3% | 47,1% |
| Total | Count | 44 | 24 | 68 |
| | % Infezione1S | 64,7% | 35,3% | 100,0% |
| | % IPERTENSIONE | 100,0% | 100,0% | 100,0% |

| Chi-SquareTests | Value | df | Sig. |
|---|---|---|---|
| Chi-Square | 1,89 | 1 | ,169 |
| N | 68 | | |

Commento: il test certifica che le Infezioni pur se più alte nei casi di IPERTENSIONE non sono statisticamente diverse dai non infetti.

**Descriptive Statistics** Dependent Variable: COMSUM somma delle comorbidità

| SEX1M | Infezione1S | Mean | sd | N |
|---|---|---|---|---|
| 0=F | 0=No | 1,18 | 1,54 | 11 |
| | 1=Si | 1,39 | 1,24 | 18 |
| | Total | 1,31 | 1,34 | 29 |
| 1=M | 0=No | 1,12 | 1,59 | 25 |
| | 1=Si | 1,79 | 1,42 | 14 |
| | Total | 1,36 | 1,55 | 39 |
| Total | 0=No | 1,14 | 1,55 | 36 |
| | 1=Si | 1,56 | 1,32 | 32 |
| | Total | 1,34 | 1,45 | 68 |

Anova di COMSUM a 2 fattori

| Source | Sum of Squares | df | Mean Square | F | sig |
|---|---|---|---|---|---|
| **SEX1M** | ,435 | 1 | ,435 | ,203 | ,653 |
| **Infezione1S** | 2,954 | 1 | 2,954 | 1,381 | ,244 |
| **SEX1M * Infezione1S** | ,816 | 1 | ,816 | ,381 | ,539 |
| **Error** | 136,911 | 64 | 2,139 | | |
| **Corrected Total** | 263,000 | 68 | | | |

Commento: Anova conferma una NON significativa influenza sul COMSUM delle infezioni o del genere.

**SEX1M * naive Crosstabulation**

| | | naive | | |
|---|---|---|---|---|
| SEX1M | | 0=No | 1=Si | Total |
| 0=F | Count | 6 | 23 | 29 |
| | % SEX | 20,7% | 79,3% | 100,0% |
| | % naive | 54,5% | 40,4% | 42,6% |
| 1=M | Count | 5 | 34 | 39 |
| | % SEX | 12,8% | 87,2% | 100,0% |
| | % naive | 45,5% | 59,6% | 57,4% |
| Total | Count | 11 | 57 | 68 |
| | % SEX | 16,2% | 83,8% | 100,0% |
| | % naive | 100,0% | 100,0% | 100,0% |

| Chi-SquareTests | Value | df | Sig. |
|---|---|---|---|
| Chi-Square | ,760 | 1 | ,383 |
| N | 68 | | |

Commento: il test certifica che naive sono equamente presenti nei 2 generi.

**Infezione1S * naive Crosstabulation**

| Infezione1S | | naive 0=No | naive 1=Si | naive Total |
|---|---|---|---|---|
| 0=No | Count | 3 | 33 | 36 |
| | % Infezione1S | 8,3% | 91,7% | 100,0% |
| | % naive | 27,3% | 57,9% | 52,9% |
| 1=SI | Count | 8 | 24 | 32 |
| | % Infezione1S | 25,0% | 75,0% | 100,0% |
| | % naive | 72,7% | 42,1% | 47,1% |
| Total | Count | 11 | 57 | 68 |
| | % Infezione1S | 16,2% | 83,8% | 100,0% |
| | % naive | 100,0% | 100,0% | 100,0% |

| Chi-SquareTests | Value | df | Sig. |
|---|---|---|---|
| Chi-Square | 3,470 | 1 | ,062 |
| N | 68 | | |

Commento: il test certifica che naive pur se più alte nei casi di NON INFEZIONI non sono statisticamente diverse dagli infetti.

**SEX1M * naive Crosstabulation**

| SEX1M | | DROP-OUT 0=No | DROP-OUT 1=Si | DROP-OUT Total |
|---|---|---|---|---|
| 0=F | Count | 22 | 7 | 29 |
| | % SEX | 75,9% | 24,1% | 100,0% |
| | % DROP-OUT | 37,3% | 77,8% | 42,6% |
| 1=M | Count | 37 | 2 | 39 |
| | % SEX | 94,9% | 5,1% | 100,0% |
| | % DROP-OUT | 62,7% | 22,2% | 57,4% |
| Total | Count | 59 | 9 | 68 |
| | % SEX | 86,8% | 13,2% | 100,0% |
| | % DROP-OUT | 100,0% | 100,0% | 100,0% |

| Chi-SquareTests | Value | df | Sig. |
|---|---|---|---|
| Chi-Square | 5,234 | 1 | **,022** |
| N | 68 | | |

Commento: il test certifica che i DROPOUT sono statisticamente più frequenti per le Femmine rispetto ai MASCHI.

**Infezione1S * naive Crosstabulation**

| Infezione1S | | DROP-OUT 0=No | DROP-OUT 1=Si | DROP-OUT Total |
|---|---|---|---|---|
| 0=No | Count | 32 | 4 | 36 |
| | % Infezione1S | 88,9% | 11,1% | 100,0% |
| | % DROP-OUT | 54,2% | 44,4% | 52,9% |
| 1=SI | Count | 27 | 5 | 32 |
| | % Infezione1S | 84,4% | 15,6% | 100,0% |
| | % DROP-OUT | 45,8% | 55,6% | 47,1% |
| Total | Count | 59 | 9 | 68 |
| | % Infezione1S | 86,8% | 13,2% | 100,0% |
| | % DROP-OUT | 100,0% | 100,0% | 100,0% |

| Chi-SquareTests | Value | df | Sig. |
|---|---|---|---|
| Chi-Square | ,301 | 1 | ,584 |
| N | 68 | | |

Commento: il test certifica che I DROPOUT SONO EQUAMENTE presenti tra gli infetti e i non infetti.

**CuraInf1S * DROP-OUT Crosstabulation**

| Cura1nf1s | | DROP-OUT 0=No | DROP-OUT 1=Si | DROP-OUT Total |
|---|---|---|---|---|
| 0=No | Count | 12 | 1 | 13 |
| | % curaInf1S | 92,3% | 7,7% | 100,0% |
| | % DROP-OUT | 44,4% | 20,0% | 40,6% |
| 1=SI | Count | 15 | 4 | 19 |
| | % curaInf1S | 78,9% | 21,1% | 100,0% |
| | % DROP-OUT | 55,6% | 80,0% | 59,4% |
| Total | Count | 27 | 5 | 32 |
| | % curaInf1S | 84,4% | 15,6% | 100,0% |
| | % DROP-OUT | 100,0% | 100,0% | 100,0% |

| Chi-SquareTests | Value | df | Sig. |
|---|---|---|---|
| Chi-Square | 1,045 | 1 | ,307 |
| N | 32 | | |

Commento: il test certifica che i DROPOUT sono distribuite tra chi ha avuto curaSi e curaNo.

**Descriptive Statistics** Dependent Variable: EVENTISUM somma di eventi avversi

| SEX1M | Infezione1S | Mean | sd | N |
|---|---|---|---|---|
| 0=F | 0=No | ,45 | 1,21 | 11 |
| | 1=Si | ,89 | 1,02 | 18 |
| | Total | ,72 | 1,10 | 29 |
| 1=M | 0=No | ,81 | 1,33 | 26 |
| | 1=Si | ,33 | ,49 | 15 |
| | Total | ,63 | 1,11 | 41 |
| Total | 0=No | ,70 | 1,29 | 37 |
| | 1=Si | ,64 | ,86 | 33 |
| | Total | ,67 | 1,10 | 70 |

Anova di EVENTISUM a 2 fattori

| Source | Sum of Squares | df | Mean Square | F | sig |
|---|---|---|---|---|---|
| **SEX1M** | ,163 | 1 | ,163 | ,135 | ,715 |
| **Infezione1S** | ,006 | 1 | ,006 | ,005 | ,942 |
| **SEX1M * Infezione1S** | 3,282 | 1 | 3,282 | 2,712 | ,104 |
| **Error** | 79,877 | 66 | 1,210 | | |
| **Corrected Total** | 83,443 | 69 | | | |

Commento: Anova conferma una NON significativa influenza sul EVENTISUM delle infezioni o del genere.

**Descriptive Statistics** Dependent Variable: PASI somma di eventi avversi

| SEX1M | Infezione1S | Mean | sd | N |
|---|---|---|---|---|
| 0=F | 0=No | 13,55 | 14,05 | 11 |
| | 1=Si | 10,72 | 10,84 | 18 |
| | Total | 11,79 | 11,99 | 29 |
| 1=M | 0=No | 11,20 | 10,36 | 25 |
| | 1=Si | 14,86 | 11,75 | 14 |
| | Total | 12,51 | 10,87 | 39 |
| Total | 0=No | 11,92 | 11,46 | 36 |
| | 1=Si | 12,53 | 11,25 | 32 |
| | Total | 12,21 | 11,28 | 68 |

Anova di PASI a 2 fattori

| Source | Sum of Squares | df | Mean Square | F | sig |
|---|---|---|---|---|---|
| **SEX1M** | 12,417 | 1 | 12,417 | ,095 | ,759 |
| **Infezione1S** | 2,696 | 1 | 2,696 | ,021 | ,886 |
| **SEX1M * Infezione1S** | 162,840 | 1 | 162,840 | 1,249 | ,268 |
| **Error** | 8342,053 | 64 | 130,345 | | |
| **Corrected Total** | 8525,118 | 67 | | | |

Commento: Anova conferma una NON significativa influenza sul PASI delle infezioni o del genere.

**Descriptive Statistics** Dependent Variable: NAPSI TOTALE_0

| SEX1M | Infezione1S | Mean | sd | N |
|---|---|---|---|---|
| 0=F | 0=No | 23,0 | 26,6 | 11 |
| | 1=Si | 13,0 | 14,4 | 18 |
| | Total | 16,8 | 20,1 | 29 |
| 1=M | 0=No | 32,3 | 37,7 | 25 |
| | 1=Si | 39,6 | 39,4 | 14 |
| | Total | 34,9 | 37,9 | 39 |
| Total | 0=No | 29,5 | 34,6 | 36 |
| | 1=Si | 24,6 | 30,7 | 32 |
| | Total | 27,2 | 32,6 | 68 |

Anova di NAPSI TOTALE_0 a 2 fattori

| Source | Sum of Squares | df | Mean Square | F | sig |
|---|---|---|---|---|---|
| **SEX1M** | 5000,638 | 1 | 5000,638 | 4,940 | **,030** |
| **Infezione1S** | 29,722 | 1 | 29,722 | ,029 | ,864 |
| **SEX1M * Infezione1S** | 1151,337 | 1 | 1151,337 | 1,137 | ,290 |
| **Error** | 64787,789 | 64 | 1012,309 | | |
| **Corrected Total** | 71414,570 | 67 | | | |

Commento: Anova conferma una significativa influenza sul PASI_TOTALE_0 dovuta al genere.

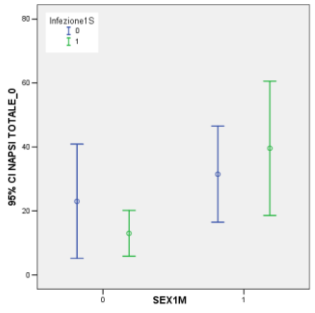


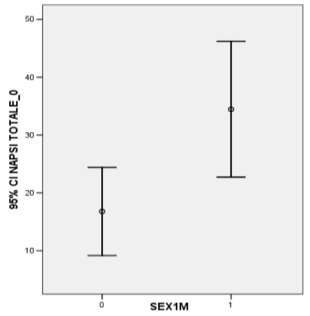


E' evidente l'effetto statistico del genere sul NAPSI TOTALE_0 dove i maschi hanno valori mediamente più alti rispetto alle femmine

**Descriptive Statistics** Dependent Variable: DLQI_0

| SEX1M | Infezione1S | Mean | sd | N |
|---|---|---|---|---|
| 0=F | 0=No | 8,45 | 6,41 | 11 |
| | 1=Si | 8,38 | 7,05 | 16 |
| | Total | 8,41 | 6,67 | 27 |
| 1=M | 0=No | 4,80 | 5,66 | 25 |
| | 1=Si | 7,57 | 7,70 | 14 |
| | Total | 5,79 | 6,51 | 39 |
| Total | 0=No | 5,92 | 6,05 | 36 |
| | 1=Si | 8,00 | 7,24 | 30 |
| | Total | 6,86 | 6,65 | 66 |

Anova di DLQI_0 a 2 fattori

| Source | Sum of Squares | df | Mean Square | F | sig |
|---|---|---|---|---|---|
| **SEX1M** | 75,045 | 1 | 75,045 | 1,726 | ,194 |
| **Infezione1S** | 27,361 | 1 | 27,361 | ,629 | ,431 |
| **SEX1M * Infezione1S** | 30,691 | 1 | 30,691 | ,706 | ,404 |
| **Error** | 2695,906 | 62 | 43,482 | | |
| **Corrected Total** | 2873,773 | 65 | | | |

Commento: Anova conferma una NON significativa influenza sul DLQI_0 delle infezioni o del genere.

**Descriptive Statistics** Dependent Variable: PAIN_VAS%_0

| SEX1M | Infezione1S | Mean | sd | N |
|---|---|---|---|---|
| 0=F | 0=No | 14,6 | 30,1 | 11 |
| | 1=Si | 31,3 | 34,8 | 16 |
| | Total | 24,4 | 33,4 | 27 |
| 1=M | 0=No | 19,9 | 29,8 | 25 |
| | 1=Si | 20,0 | 25,1 | 14 |
| | Total | 19,9 | 27,8 | 39 |
| Total | 0=No | 18,3 | 29,5 | 36 |
| | 1=Si | 26,0 | 30,7 | 30 |
| | Total | 21,8 | 30,1 | 66 |

Anova di PAIN_VAS%_0 a 2 fattori

| Source | Sum of Squares | df | Mean Square | F | sig |
|---|---|---|---|---|---|
| **SEX1M** | 132,128 | 1 | 132,128 | ,144 | ,705 |
| **Infezione1S** | 1068,826 | 1 | 1068,826 | 1,168 | ,284 |
| **SEX1M * Infezione1S** | 1038,550 | 1 | 1038,550 | 1,135 | ,291 |
| **Error** | 56716,367 | 62 | 914,780 | | |
| **Corrected Total** | 58861,591 | 65 | | | |

Commento: Anova conferma una NON significativa influenza sul PAIN_VAS%_0 delle infezioni o del genere.

**Descriptive Statistics** Dependent Variable: itch_0

| SEX1M | Infezione1S | Mean | sd | N | Anova di itch_0 a 2 fattori | | | | | |
|---|---|---|---|---|---|---|---|---|---|---|
| 0=F | 0=No | 8,45 | 1,97 | 11 | Source | Sum of Squares | df | Mean Square | F | sig |
| | 1=Si | 8,13 | 2,03 | 16 | **SEX1M** | 31,871 | 1 | 31,871 | 5,142 | **,027** |
| | Total | 8,26 | 1,97 | 27 | **Infezione1S** | 1,250 | 1 | 1,250 | ,202 | ,655 |
| 1=M | 0=No | 6,96 | 2,75 | 25 | **SEX1M*Infezione1S** | ,027 | 1 | ,027 | ,004 | ,948 |
| | 1=Si | 6,71 | 2,81 | 14 | **Error** | 384,294 | 62 | 6,198 | | |
| | Total | 6,87 | 2,74 | 39 | **Corrected Total** | 416,258 | 65 | | | |
| Total | 0=No | 7,42 | 2,60 | 36 | Commento: Anova conferma una NON significativa influenza sul DLQI_0 delle infezioni o del genere. | | | | | |
| | 1=Si | 7,47 | 2,49 | 30 | | | | | | |
| | Total | 7,44 | 2,53 | 66 | | | | | | |

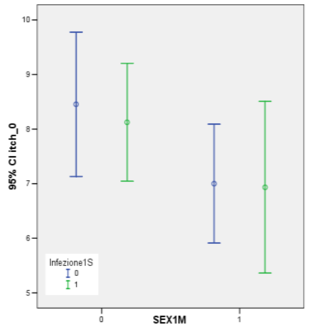


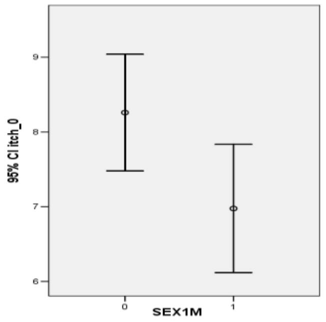


E’ evidente l’effetto statistico del genere sul itch_0 dove i maschi hanno valori mediamente più bassi rispetto alle femmine

**Descriptive Statistics** Dependent Variable: PAIN VAS_0

| SEX1M | Infezione1S | Mean | sd | N | Anova di itch_0 a 2 fattori | | | | | |
|---|---|---|---|---|---|---|---|---|---|---|
| 0=F | 0=No | 14,6 | 30,1 | 11 | Source | Sum of Squares | df | Mean Square | F | sig |
| | 1=Si | 31,3 | 34,8 | 16 | **SEX1M** | 132,128 | 1 | 132,128 | ,144 | ,705 |
| | Total | 24,4 | 33,4 | 27 | **Infezione1S** | 1068,826 | 1 | 1068,826 | 1,168 | ,284 |
| 1=M | 0=No | 19,9 | 29,8 | 25 | **SEX1M * Infezione1S** | 1038,550 | 1 | 1038,550 | 1,135 | ,291 |
| | 1=Si | 20,0 | 25,1 | 14 | **Error** | 56716,367 | 62 | 914,780 | | |
| | Total | 19,9 | 27,8 | 39 | **Corrected Total** | 58861,591 | 65 | | | |
| Total | 0=No | 18,3 | 29,5 | 36 | Commento: Anova conferma una NON significativa influenza sul PAIN VAS_0 delle infezioni o del genere. | | | | | |
| | 1=Si | 26,0 | 30,7 | 30 | | | | | | |
| | Total | 21,8 | 30,1 | 66 | | | | | | |

**Descriptive Statistics** Dependent Variable: GLICEMIA_0

| SEX1M | Infezione1S | Mean | sd | N | Anova di itch_0 a 2 fattori | | | | | |
|---|---|---|---|---|---|---|---|---|---|---|
| 0=F | 0=No | 91,0 | 10,1 | 10 | Source | Sum of Squares | df | Mean Square | F | sig |
| | 1=Si | 98,3 | 18,9 | 15 | **SEX1M** | 1644,622 | 1 | 1644,622 | 1,342 | ,251 |
| | Total | 95,4 | 16,1 | 25 | **Infezione1S** | 29,287 | 1 | 29,287 | ,024 | ,878 |
| 1=M | 0=No | 110,5 | 49,6 | 24 | **SEX1M * Infezione1S** | 1064,256 | 1 | 1064,256 | ,868 | ,355 |
| | 1=Si | 100,4 | 26,7 | 13 | **Error** | 71095,969 | 58 | 1225,793 | | |
| | Total | 107,0 | 42,8 | 37 | **Corrected Total** | 74294,774 | 61 | | | |
| Total | 0=No | 104,8 | 42,7 | 34 | Commento: Anova conferma una NON significativa influenza sul GLICEMIA_0 delle infezioni o del genere. | | | | | |
| | 1=Si | 99,3 | 22,4 | 28 | | | | | | |
| | Total | 102,3 | 34,9 | 62 | | | | | | |

**Descriptive Statistics** Dependent Variable: COL TOT _0

| SEX1M | Infezione1S | Mean | sd | N | Anova di itch_0 a 2 fattori | | | | | |
|---|---|---|---|---|---|---|---|---|---|---|
| 0=F | 0=No | 192,3 | 28,1 | 10 | Source | Sum of Squares | df | Mean Square | F | sig |
| | 1=Si | 199,0 | 39,7 | 15 | **SEX1M** | 507,295 | 1 | 507,295 | ,378 | ,541 |
| | Total | 196,3 | 35,0 | 25 | **Infezione1S** | 238,606 | 1 | 238,606 | ,178 | ,675 |
| 1=M | 0=No | 189,0 | 33,7 | 24 | **SEX1M * Infezione1S** | 97,763 | 1 | 97,763 | ,073 | ,788 |
| | 1=Si | 190,4 | 42,8 | 14 | **Error** | 79098,487 | 59 | 1340,652 | | |
| | Total | 189,5 | 36,8 | 38 | **Corrected Total** | 80088,317 | 62 | | | |
| Total | 0=No | 189,9 | 31,8 | 34 | Commento: Anova conferma una NON significativa influenza sul COLTOT _0 delle infezioni o del genere. | | | | | |
| | 1=Si | 194,9 | 40,7 | 29 | | | | | | |
| | Total | 192,2 | 35,9 | 63 | | | | | | |

**Descriptive Statistics** Dependent Variable: HLD_0

| SEX1M | Infezione1S | Mean | sd | N |
|---|---|---|---|---|
| 0=F | 0=No | 67,0 | 16,5 | 10 |
| | 1=Si | 60,4 | 18,0 | 15 |
| | Total | 63,0 | 17,4 | 25 |
| 1=M | 0=No | 53,9 | 20,2 | 24 |
| | 1=Si | 50,9 | 14,6 | 13 |
| | Total | 52,8 | 18,3 | 37 |
| Total | 0=No | 57,8 | 19,0 | 34 |
| | 1=Si | 56,0 | 16,9 | 28 |
| | Total | 57,0 | 18,5 | 62 |

Anova di itch_0 a 2 fattori

| Source | Sum of Squares | df | Mean Square | F | sig |
|---|---|---|---|---|---|
| **SEX1M** | 1796,426 | 1 | 1796,426 | 5,511 | ,022 |
| **Infezione1S** | 327,841 | 1 | 327,841 | 1,006 | ,320 |
| **SEX1M*Infezione1S** | 43,670 | 1 | 43,670 | ,134 | ,716 |
| **Error** | 18905,126 | 58 | 325,950 | | |
| **Corrected Total** | 20798,855 | 61 | | | |

Commento: Anova conferma una significativa influenza sul HDL_0 dovuta al genere.

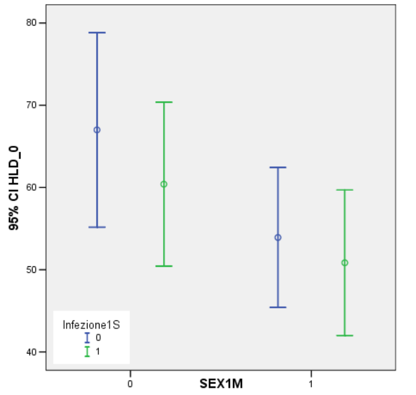


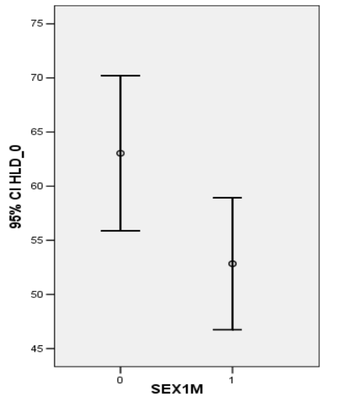


E' evidente l'effetto statistico del genere sul HLD_0 dove i maschi hanno valori mediamente più bassi rispetto alle femmine

**Descriptive Statistics** Dependent Variable: LDL _0

| SEX1M | Infezione1S | Mean | sd | N |
|---|---|---|---|---|
| 0=F | 0=No | 111,0 | 27,6 | 10 |
| | 1=Si | 110,3 | 29,1 | 15 |
| | Total | 110,6 | 27,9 | 25 |
| 1=M | 0=No | 114,4 | 34,3 | 24 |
| | 1=Si | 113,9 | 36,0 | 13 |
| | Total | 114,2 | 34,4 | 37 |
| Total | 0=No | 113,4 | 32,1 | 34 |
| | 1=Si | 112,0 | 31,9 | 28 |
| | Total | 112,8 | 31,8 | 62 |

Anova di itch_0 a 2 fattori

| Source | Sum of Squares | df | Mean Square | F | sig |
|---|---|---|---|---|---|
| **SEX1M** | 175,381 | 1 | 175,381 | ,166 | ,685 |
| **Infezione1S** | 5,277 | 1 | 5,277 | ,005 | ,944 |
| **SEX1M * Infezione1S** | ,201 | 1 | ,201 | ,000 | ,989 |
| **Error** | 61293,690 | 58 | 1056,788 | | |
| **Corrected Total** | 61501,371 | 61 | | | |

Commento: Anova conferma una NON significativa influenza sul LDL_0 delle infezioni o del genere.

**Descriptive Statistics** Dependent Variable: TRIGLICERIDI_0

| SEX1M | Infezione1S | Mean | sd | N |
|---|---|---|---|---|
| 0=F | 0=No | 89,3 | 28,9 | 10 |
| | 1=Si | 105,8 | 53,0 | 15 |
| | Total | 99,2 | 44,9 | 25 |
| 1=M | 0=No | 124,8 | 112,0 | 24 |
| | 1=Si | 130,4 | 66,7 | 14 |
| | Total | 126,8 | 96,8 | 38 |
| Total | 0=No | 114,4 | 96,1 | 34 |
| | 1=Si | 117,7 | 60,2 | 29 |
| | Total | 115,9 | 81,0 | 63 |

Anova di itch_0 a 2 fattori

| Source | Sum of Squares | df | Mean Square | F | sig |
|---|---|---|---|---|---|
| **SEX1M** | 12889,030 | 1 | 12889,030 | 1,934 | ,170 |
| **Infezione1S** | 1740,356 | 1 | 1740,356 | ,261 | ,611 |
| **SEX1M * Infezione1S** | 427,377 | 1 | 427,377 | ,064 | ,801 |
| **Error** | 393209,673 | 59 | 6664,571 | | |
| **Corrected Total** | 406638,984 | 62 | | | |

Commento: Anova conferma una NON significativa influenza sul TRIGLIGERIDI_0 delle infezioni o del genere.

**Descriptive Statistics** Dependent Variable: VES_0

| SEX1M | Infezione1S | Mean | sd | N |
|---|---|---|---|---|
| 0=F | 0=No | 22,2 | 29,0 | 10 |
| | 1=Si | 26,7 | 18,7 | 15 |
| | Total | 24,9 | 22,9 | 25 |
| 1=M | 0=No | 13,5 | 10,2 | 24 |
| | 1=Si | 11,9 | 10,6 | 13 |
| | Total | 12,9 | 10,2 | 37 |
| Total | 0=No | 16,1 | 17,8 | 34 |
| | 1=Si | 19,8 | 17,0 | 28 |
| | Total | 17,8 | 17,4 | 62 |

Anova di itch_0 a 2 fattori

| Source | Sum of Squares | df | Mean Square | F | sig |
|---|---|---|---|---|---|
| **SEX1M** | 1950,368 | 1 | 1950,368 | 6,975 | ,011 |
| **Infezione1S** | 29,067 | 1 | 29,067 | ,104 | ,748 |
| **SEX1M*Infezione1S** | 134,199 | 1 | 134,199 | ,480 | ,491 |
| **Error** | 16218,226 | 58 | 279,625 | | |
| **Corrected Total** | 18513,371 | 61 | | | |

Commento: Anova conferma una significativa influenza sul VES_0 dovuta al genere.

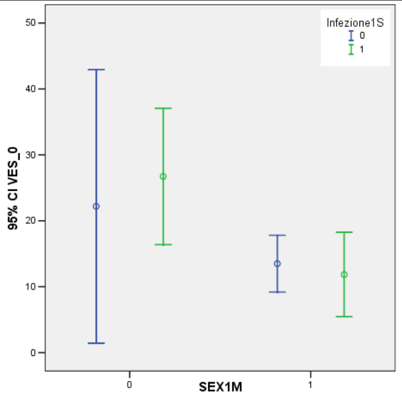


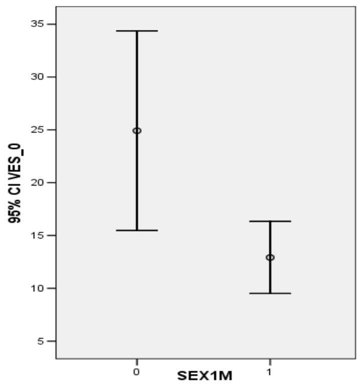


E’ evidente l’effetto statistico del genere sul VES_0 dove i maschi hanno valori mediamente più bassi rispetto alle femmine

**Descriptive Statistics** Dependent Variable: PCR_0

| SEX1M | Infezione1S | Mean | sd | N |
|---|---|---|---|---|
| 0=F | 0=No | 5,30 | 12,43 | 10 |
| | 1=Si | 1,76 | 2,29 | 15 |
| | Total | 3,19 | 8,01 | 25 |
| 1=M | 0=No | 2,66 | 5,49 | 24 |
| | 1=Si | 4,59 | 10,41 | 14 |
| | Total | 3,37 | 7,60 | 38 |
| Total | 0=No | 3,44 | 8,04 | 34 |
| | 1=Si | 3,13 | 7,42 | 29 |
| | Total | 3,30 | 7,70 | 63 |

Anova di itch_0 a 2 fattori

| Source | Sum of Squares | df | Mean Square | F | sig |
|---|---|---|---|---|---|
| **SEX1M** | ,098 | 1 | ,098 | ,002 | ,968 |
| **Infezione1S** | 9,408 | 1 | 9,408 | ,156 | ,695 |
| **SEX1M * Infezione1S** | 107,878 | 1 | 107,878 | 1,783 | ,187 |
| **Error** | 3568,981 | 59 | 60,491 | | |
| **Corrected Total** | 3678,576 | 62 | | | |

Commento: Anova conferma una NON significativa influenza sul PCR_0 delle infezioni o del genere.

**Descriptive Statistics** Dependent Variable: HBA1C_0

| SEX1M | Infezione1S | Mean | sd | N |
|---|---|---|---|---|
| 0=F | 0=No | 37,5 | 5,523 | 10 |
| | 1=Si | 37,9 | 5,946 | 15 |
| | Total | 37,8 | 5,666 | 25 |
| 1=M | 0=No | 40,8 | 13,314 | 24 |
| | 1=Si | 38,5 | 11,370 | 13 |
| | Total | 40,0 | 12,556 | 37 |
| Total | 0=No | 39,8 | 11,585 | 34 |
| | 1=Si | 38,2 | 8,710 | 28 |
| | Total | 39,1 | 10,338 | 62 |

Anova di itch_0 a 2 fattori

| Source | Sum of Squares | df | Mean Square | F | sig |
|---|---|---|---|---|---|
| **SEX1M** | 51,711 | 1 | 51,711 | ,469 | ,496 |
| **Infezione1S** | 12,891 | 1 | 12,891 | ,117 | ,734 |
| **SEX1M * Infezione1S** | 27,177 | 1 | 27,177 | ,246 | ,622 |
| **Error** | 6397,910 | 58 | 110,309 | | |
| **Corrected Total** | 6519,602 | 61 | | | |

Commento: Anova conferma una NON significativa influenza sul HBA1C_0 delle infezioni o del genere.

**Descriptive Statistics** Dependent Variable: T0 IL-10 (pg/ml)

| SEX1M | Infezione1S | Mean | sd | N |
|---|---|---|---|---|
| 0=F | 0=No | ,40 | ,18 | 7 |
| | 1=Si | ,36 | ,21 | 14 |
| | Total | ,37 | ,20 | 21 |
| 1=M | 0=No | ,39 | ,21 | 24 |
| | 1=Si | ,54 | ,29 | 12 |
| | Total | ,44 | ,25 | 36 |
| Total | 0=No | ,40 | ,20 | 31 |
| | 1=Si | ,44 | ,26 | 26 |
| | Total | ,42 | ,23 | 57 |

| Anova di itch_0 a 2 fattori | | | | | |
|---|---|---|---|---|---|
| Source | Sum of Squares | df | Mean Square | F | sig |
| **SEX1M** | ,088 | 1 | ,088 | 1,670 | ,202 |
| **Infezione1S** | ,036 | 1 | ,036 | ,684 | ,412 |
| **SEX1M * Infezione1S** | ,095 | 1 | ,095 | 1,793 | ,186 |
| **Error** | 2,801 | 53 | ,053 | | |
| **Corrected Total** | 3,036 | 56 | | | |

Commento: Anova conferma una NON significativa influenza sul T0IL-10 (pg/ml) delle infezioni o del genere.

**Descriptive Statistics** Dependent Variable: T0 TGF-B (ng/ml)

| SEX1M | Infezione1S | Mean | sd | N |
|---|---|---|---|---|
| 0=F | 0=No | 53,80 | 19,91 | 7 |
| | 1=Si | 68,00 | 43,24 | 13 |
| | Total | 63,03 | 36,80 | 20 |
| 1=M | 0=No | 66,44 | 31,56 | 24 |
| | 1=Si | 71,55 | 21,51 | 12 |
| | Total | 68,15 | 28,39 | 36 |
| Total | 0=No | 63,59 | 29,53 | 31 |
| | 1=Si | 69,70 | 33,91 | 25 |
| | Total | 66,32 | 31,41 | 56 |

| Anova di itch_0 a 2 fattori | | | | | |
|---|---|---|---|---|---|
| Source | Sum of Squares | df | Mean Square | F | sig |
| **SEX1M** | 761,455 | 1 | 761,455 | ,750 | ,391 |
| **Infezione1S** | 1081,375 | 1 | 1081,375 | 1,065 | ,307 |
| **SEX1M * Infezione1S** | 239,909 | 1 | 239,909 | ,236 | ,629 |
| **Error** | 52811,016 | 52 | 1015,596 | | |
| **Corrected Total** | 54274,423 | 55 | | | |

Commento: Anova conferma una NON significativa influenza sul T0 TGF-B (ng/ml) delle infezioni o del genere.

**Descriptive Statistics** Dependent Variable: T0 IL-17 (pg/ml)

| SEX1M | Infezione1S | Mean | Sd | N |
|---|---|---|---|---|
| 0=F | 0=No | ,120 | ,007 | 7 |
| | 1=Si | ,130 | ,068 | 14 |
| | Total | ,130 | ,056 | 21 |
| 1=M | 0=No | ,115 | ,040 | 23 |
| | 1=Si | ,152 | ,078 | 12 |
| | Total | ,128 | ,057 | 35 |
| Total | 0=No | ,117 | ,035 | 30 |
| | 1=Si | ,143 | ,071 | 26 |
| | Total | ,129 | ,056 | 56 |

| Anova di itch_0 a 2 fattori | | | | | |
|---|---|---|---|---|---|
| Source | Sum of Squares | df | Mean Square | F | sig |
| **SEX1M** | ,000 | 1 | ,000 | ,098 | ,756 |
| **Infezione1S** | ,007 | 1 | ,007 | 2,350 | ,131 |
| **SEX1M * Infezione1S** | ,002 | 1 | ,002 | ,618 | ,435 |
| **Error** | ,159 | 52 | ,003 | | |
| **Corrected Total** | ,171 | 55 | | | |

Commento: Anova conferma una NON significativa influenza sul T0IL-17 (pg/ml) delle infezioni o del genere.

| Descrizione | | | | | | | Anova oneway | | | | | |
|---|---|---|---|---|---|---|---|---|---|---|---|---|
| | | | | | | | | | | | | |
| | Infezione | N | Mean | SD | Min. | Max. | | Sum of sq | df | Mean$^2$ | F | Sig. |
| PASI_12 | 0=N | 31 | 1,39 | 2,12 | 0 | 8 | Between | ,047 | 1 | ,047 | ,011 | ,917 |
| | 1=S | 27 | 1,44 | 2,04 | 0 | 6 | Within | 244,022 | 56 | 4,358 | | |
| | total | 58 | 1,41 | 2,07 | 0 | 8 | Total | 244,069 | 57 | | | |
| NAPSI | 0=N | 31 | 8,74 | 22,64 | 0 | 100 | Between | 280,478 | 1 | 280,478 | ,820 | ,369 |
| TOT_12 | 1=S | 27 | 4,33 | 12,05 | 0 | 60 | Within | 19153,935 | 56 | 342,035 | | |
| | total | 58 | 6,69 | 18,47 | 0 | 100 | Total | 19434,414 | 57 | | | |
| itch_12 | 0=N | 31 | ,74 | 1,34 | 0 | 4 | Between | 3,329 | 1 | 3,329 | ,958 | ,332 |
| | 1=S | 27 | 1,22 | 2,33 | 0 | 10 | Within | 194,602 | 56 | 3,475 | | |
| | total | 58 | ,97 | 1,86 | 0 | 10 | Total | 197,931 | 57 | | | |
| DLQI_12 | 0=N | 31 | ,48 | ,77 | 0 | 2 | Between | ,307 | 1 | ,307 | ,238 | ,627 |
| | 1=S | 27 | ,63 | 1,45 | 0 | 7 | Within | 72,038 | 56 | 1,286 | | |
| | total | 58 | ,55 | 1,13 | 0 | 7 | Total | 72,345 | 57 | | | |
| PAIN | 0=N | 31 | 3,06 | 6,91 | 0 | 30 | Between | 71,879 | 1 | 71,879 | ,684 | ,412 |
| VAS_12 | 1=S | 27 | 5,30 | 13,09 | 0 | 60 | Within | 5885,501 | 56 | 105,098 | | |
| | total | 58 | 4,10 | 10,22 | 0 | 60 | Total | 5957,379 | 57 | | | |

Commento: Anova conferma una NON significativa influenza di Infezione sulle suddette variabili

| Descrizione | | | | | | | Anova oneway | | | | | |
|---|---|---|---|---|---|---|---|---|---|---|---|---|
| | | | | | | | | | | | | |
| | TipoINF | N | Mean | SD | Min. | Max. | | Sum of sq | df | Mean$^2$ | F | Sig. |
| PASI_12 | 1 | 16 | 1,44 | 2,60 | 0 | 6 | Between | 2,329 | 2 | 1,165 | ,263 | ,771 |
| | 2 | 10 | 1,60 | 3,00 | 0 | 6 | Within | 106,338 | 24 | 4,431 | | |
| | 3 | 1 | ,00 | . | 0 | 0 | Total | 108,667 | 26 | | | |
| | Total | 27 | 1,44 | 2,25 | 0 | 6 | | | | | | |
| NAPSI | 1 | 16 | 2,31 | 5,36 | 0 | 20 | Between | 218,563 | 2 | 109,281 | ,737 | ,489 |
| TOT_12 | 2 | 10 | 8,00 | 18,64 | 0 | 60 | Within | 3559,438 | 24 | 148,310 | | |
| | 3 | 1 | ,00 | . | 0 | 0 | Total | 3778,000 | 26 | | | |
| | Total | 27 | 4,33 | 12,05 | 0 | 60 | | | | | | |
| itch_12 | 1 | 16 | 1,19 | 2,74 | 0 | 10 | Between | 1,829 | 2 | ,915 | ,158 | ,855 |
| | 2 | 10 | 1,40 | 1,71 | 0 | 5 | Within | 138,838 | 24 | 5,785 | | |
| | 3 | 1 | ,00 | . | 0 | 0 | Total | 140,667 | 26 | | | |
| | Total | 27 | 1,22 | 2,33 | 0 | 10 | | | | | | |
| DLQI_12 | 1 | 16 | ,75 | 1,77 | 0 | 7 | Between | ,796 | 2 | ,398 | ,179 | ,838 |
| | 2 | 10 | ,50 | ,85 | 0 | 2 | Within | 53,500 | 24 | 2,229 | | |
| | 3 | 1 | ,00 | . | 0 | 0 | Total | 54,296 | 26 | | | |
| | Total | 27 | ,63 | 1,45 | 0 | 7 | | | | | | |
| PAIN | 1 | 16 | 6,44 | 16,22 | 0 | 60 | Between | 95,692 | 2 | 47,846 | ,264 | ,770 |
| VAS_12 | 2 | 10 | 3,00 | 6,75 | 0 | 20 | Within | 4355,938 | 24 | 181,497 | | |
| | 3 | 1 | 10,00 | . | 10 | 10 | Total | 4451,630 | 26 | | | |
| | Total | 27 | 5,30 | 13,09 | 0 | 60 | | | | | | |

Commento: Anova conferma una NON significativa influenza di TipoINF sulle suddette variabili

| Descrizione | | | | | | | Anova oneway | | | | | |
|---|---|---|---|---|---|---|---|---|---|---|---|---|
| | | | | | | | | | | | | |
| | CuraINF | N | Mean | SD | Min. | Max. | | Sum of sq | df | Mean$^2$ | F | Sig. |
| PASI_12 | 0=N | 12 | 1,33 | 1,88 | 0 | 6 | Between | 117,600 | 1 | 117,600 | ,803 | ,379 |
| | 1=S | 15 | 1,53 | 2,23 | 0 | 6 | Within | 3660,400 | 25 | 146,416 | | |
| | total | 27 | 1,44 | 2,04 | 0 | 6 | Total | 3778,000 | 26 | | | |
| NAPSI | 0=N | 12 | 6,67 | 17,15 | 0 | 60 | Between | ,067 | 1 | ,067 | ,012 | ,914 |
| TOT_12 | 1=S | 15 | 2,47 | 5,51 | 0 | 20 | Within | 140,600 | 25 | 5,624 | | |
| | total | 27 | 4,33 | 12,05 | 0 | 60 | Total | 140,667 | 26 | | | |
| itch_12 | 0=N | 12 | 1,17 | 1,64 | 0 | 5 | Between | ,363 | 1 | ,363 | ,168 | ,685 |
| | 1=S | 15 | 1,27 | 2,82 | 0 | 10 | Within | 53,933 | 25 | 2,157 | | |
| | total | 27 | 1,22 | 2,33 | 0 | 10 | Total | 54,296 | 26 | | | |
| DLQI_12 | 0=N | 12 | ,50 | ,80 | 0 | 2 | Between | 83,230 | 1 | 83,230 | ,476 | ,496 |
| | 1=S | 15 | ,73 | 1,83 | 0 | 7 | Within | 4368,400 | 25 | 174,736 | | |
| | total | 27 | ,63 | 1,45 | 0 | 7 | Total | 4451,630 | 26 | | | |
| PAIN | 0=N | 12 | 3,33 | 6,51 | 0 | 20 | Between | 117,600 | 1 | 117,600 | ,803 | ,379 |
| VAS_12 | 1=S | 15 | 6,87 | 16,70 | 0 | 60 | Within | 3660,400 | 25 | 146,416 | | |
| | total | 27 | 5,30 | 13,09 | 0 | 60 | Total | 3778,000 | 26 | | | |

Commento: Anova conferma una NON significativa influenza di CuraINF sulle suddette variabili

| Descrizione | | | | | | | Anova oneway | | | | | |
|---|---|---|---|---|---|---|---|---|---|---|---|---|
| | | | | | | | | | | | | |
| | CuraINF | N | Mean | SD | Min. | Max. | | Sum of sq | df | Mean$^2$ | F | Sig. |
| T12 IL-10 | 0=N | 10 | ,194 | ,157 | ,00 | ,44 | Between | ,000 | 1 | ,000 | ,012 | ,915 |
| (pg/ml) | 1=S | 14 | ,202 | ,222 | ,00 | ,65 | Within | ,863 | 22 | ,039 | | |
| | total | 24 | ,199 | ,194 | ,00 | ,65 | Total | ,863 | 23 | | | |
| T12 TGFB | 0=N | 10 | 50,770 | 14,680 | 25,40 | 70,34 | Between | 343,808 | 1 | 343,808 | 1,521 | ,230 |
| (ng/ml) | 1=S | 14 | 43,093 | 15,274 | 25,90 | 69,69 | Within | 4972,562 | 22 | 226,026 | | |
| | total | 24 | 46,292 | 15,204 | 25,40 | 70,34 | Total | 5316,371 | 23 | | | |
| T12 IL-17 | 0=N | 11 | ,0197 | ,0203 | ,005 | ,063 | Between | ,001 | 1 | ,001 | ,710 | ,408 |
| (pg/ml) | 1=S | 14 | ,0301 | ,0368 | ,005 | ,127 | Within | ,022 | 23 | ,001 | | |
| | total | 25 | ,0255 | ,0306 | ,005 | ,127 | Total | ,022 | 24 | | | |

Commento: Anova conferma una NON significativa influenza di CuraINF sulle suddette variabili

| Descrizione | | | | | | | Anova oneway | | | | | |
|---|---|---|---|---|---|---|---|---|---|---|---|---|
| | | | | | | | | | | | | |
| | TipoINF | N | Mean | SD | Min. | Max. | | Sum of sq | df | $Mean^2$ | F | Sig. |
| T12 IL-10 | 1 | 15 | ,189 | ,220 | ,00 | ,65 | Between | ,010 | 2 | ,005 | ,124 | ,884 |
| (pg/ml) | 2 | 8 | ,206 | ,159 | ,00 | ,44 | Within | ,853 | 21 | ,041 | | |
| | 3 | 1 | ,290 | . | ,29 | ,29 | Total | ,863 | 23 | | | |
| | Total | 24 | ,199 | ,194 | ,00 | ,65 | | | | | | |
| T12 TGFB | 1 | 15 | 42,657 | 14,815 | 25,90 | 69,69 | Between | 611,155 | 2 | 305,577 | 1,364 | ,277 |
| (ng/ml) | 2 | 8 | 51,279 | 15,270 | 25,40 | 70,34 | Within | 4705,216 | 21 | 224,058 | | |
| | 3 | 1 | 60,920 | . | 60,92 | 60,92 | Total | 5316,371 | 23 | | | |
| | Total | 24 | 46,292 | 15,204 | 25,40 | 70,34 | | | | | | |
| T12 IL-17 | 1 | 15 | ,029 | ,036 | ,005 | ,127 | Between | ,001 | 2 | ,000 | ,308 | ,738 |
| (pg/ml) | 2 | 9 | ,022 | ,022 | ,005 | ,063 | Within | ,022 | 22 | ,001 | | |
| | 3 | 1 | ,006 | . | ,006 | ,006 | Total | ,022 | 24 | | | |
| | Total | 25 | ,026 | ,031 | ,005 | ,127 | | ,010 | 2 | ,005 | ,124 | ,884 |

Commento: Anova conferma una NON significativa influenza di TipoINF sulle suddette variabili

| Descrizione | | | | | | | Anova oneway | | | | | |
|---|---|---|---|---|---|---|---|---|---|---|---|---|
| | | | | | | | | | | | | |
| | Infezione | N | Mean | SD | Min. | Max. | | Sum of sq | df | $Mean^2$ | F | Sig. |
| GLICEMIA_12 | 0=N | 11 | 90,0 | 8,6 | 76 | 102 | Between | 90,270 | 1 | 90,270 | ,618 | ,439 |
| | 1=S | 17 | 93,7 | 13,8 | 72 | 131 | Within | 3796,471 | 26 | 146,018 | | |
| | total | 28 | 92,2 | 12,0 | 72 | 131 | Total | 3886,741 | 27 | | | |
| HBA1C_12 | 0=N | 11 | 37,0 | 4,8 | 31 | 48 | Between | ,000 | 1 | ,000 | ,000 | 1,000 |
| | 1=S | 17 | 37,0 | 4,4 | 29 | 42 | Within | 542,000 | 26 | 20,846 | | |
| | total | 28 | 37,0 | 4,5 | 29 | 48 | Total | 542,000 | 27 | | | |
| COL TOT_12 | 0=N | 11 | 181,7 | 30,5 | 147 | 249 | Between | 45,482 | 1 | 45,482 | ,070 | ,793 |
| | 1=S | 17 | 179,1 | 21,7 | 155 | 234 | Within | 16819,947 | 26 | 646,921 | | |
| | total | 28 | 180,1 | 25,0 | 147 | 249 | Total | 16865,429 | 27 | | | |
| HLD_12 | 0=N | 11 | 48,8 | 20,5 | 22 | 102 | Between | 40,941 | 1 | 40,941 | ,124 | ,727 |
| | 1=S | 17 | 51,3 | 16,5 | 30 | 86 | Within | 8565,166 | 26 | 329,429 | | |
| | total | 28 | 50,3 | 17,9 | 22 | 102 | Total | 8606,107 | 27 | | | |
| LDL_12 | 0=N | 11 | 113,2 | 29,2 | 57 | 147 | Between | 3,278 | 1 | 3,278 | ,003 | ,954 |
| | 1=S | 17 | 113,9 | 32,5 | 50 | 180 | Within | 25437,401 | 26 | 978,362 | | |
| | total | 28 | 113,6 | 30,7 | 50 | 180 | Total | 25440,679 | 27 | | | |
| TRIGLICERIDI_12 | 0=N | 11 | 93,1 | 26,7 | 58 | 140 | Between | 24,341 | 1 | 24,341 | ,027 | ,872 |
| | 1=S | 17 | 95,0 | 32,4 | 40 | 149 | Within | 23876,909 | 26 | 918,343 | | |
| | total | 28 | 94,3 | 29,8 | 40 | 149 | Total | 23901,250 | 27 | | | |
| VES_12 | 0=N | 11 | 13,4 | 12,9 | 2 | 38 | Between | ,032 | 1 | ,032 | ,000 | ,989 |
| | 1=S | 17 | 13,3 | 12,6 | 2 | 40 | Within | 4200,075 | 26 | 161,541 | | |
| | total | 28 | 13,3 | 12,5 | 2 | 40 | Total | 4200,107 | 27 | | | |
| PCR_12 | 0=N | 11 | 1,90 | 3,13 | ,2 | 10,6 | Between | 1,341 | 1 | 1,341 | ,222 | ,642 |
| | 1=S | 17 | 1,41 | 1,93 | ,1 | 6,6 | Within | 157,210 | 26 | 6,047 | | |
| | total | 28 | 1,58 | 2,42 | ,1 | 10,6 | Total | 158,551 | 27 | | | |

Commento: Anova conferma una NON significativa influenza di Infezione sulle suddette variabili

| Descrizione | | | | | | | Anova oneway | | | | | |
|---|---|---|---|---|---|---|---|---|---|---|---|---|
| | | | | | | | | | | | | |
| | TipoINF | N | Mean | SD | Min. | Max. | | Sum of sq | df | Mean$^2$ | F | Sig. |
| GLICEMIA_12 | 1 | 10 | 97,4 | 16,3 | 72 | 131 | Between | 640,737 | 2 | 320,369 | 1,852 | ,193 |
| | 2 | 6 | 85,7 | 3,0 | 82 | 90 | Within | 2421,733 | 14 | 172,981 | | |
| | 3 | 1 | 104,5 | . | 105 | 105 | Total | 3062,471 | 16 | | | |
| | Total | 17 | 93,7 | 13,8 | 72 | 131 | | | | | | |
| HBA1C_12 | 1 | 10 | 36,2 | 5,1 | 29 | 42 | Between | 16,400 | 2 | 8,200 | ,386 | ,687 |
| | 2 | 6 | 38,0 | 3,6 | 33 | 42 | Within | 297,600 | 14 | 21,257 | | |
| | 3 | 1 | 39,0 | . | 39 | 39 | Total | 314,000 | 16 | | | |
| | Total | 17 | 37,0 | 4,4 | 29 | 42 | | | | | | |
| COL TOT_12 | 1 | 10 | 181,3 | 25,2 | 155 | 234 | Between | 134,331 | 2 | 67,166 | ,127 | ,882 |
| | 2 | 6 | 175,3 | 18,5 | 160 | 198 | Within | 7403,433 | 14 | 528,817 | | |
| | 3 | 1 | 180,0 | . | 180 | 180 | Total | 7537,765 | 16 | | | |
| | Total | 17 | 179,1 | 21,7 | 155 | 234 | | | | | | |
| HLD_12 | 1 | 10 | 53,5 | 17,1 | 33 | 86 | Between | 215,696 | 2 | 107,848 | ,364 | ,701 |
| | 2 | 6 | 49,7 | 17,3 | 30 | 77 | Within | 4149,833 | 14 | 296,417 | | |
| | 3 | 1 | 39,0 | . | 39 | 39 | Total | 4365,529 | 16 | | | |
| | Total | 17 | 51,3 | 16,5 | 30 | 86 | | | | | | |
| LDL_12 | 1 | 10 | 115,1 | 33,1 | 80 | 180 | Between | 127,531 | 2 | 63,766 | ,053 | ,948 |
| | 2 | 6 | 110,7 | 37,1 | 50 | 155 | Within | 16776,233 | 14 | 1198,302 | | |
| | 3 | 1 | 121,0 | . | 121 | 121 | Total | 16903,765 | 16 | | | |
| | Total | 17 | 113,9 | 32,5 | 50 | 180 | | | | | | |
| TRIGLICERIDI_12 | 1 | 10 | 96,0 | 29,8 | 40 | 137 | Between | 88,667 | 2 | 44,333 | ,037 | ,964 |
| | 2 | 6 | 92,3 | 41,7 | 47 | 149 | Within | 16661,333 | 14 | 1190,095 | | |
| | 3 | 1 | 101,0 | . | 101 | 101 | Total | 16750,000 | 16 | | | |
| | Total | 17 | 95,0 | 32,4 | 40 | 149 | | | | | | |
| VES_12 | 1 | 10 | 8,6 | 6,6 | 2 | 25 | Between | 651,796 | 2 | 325,898 | 2,420 | ,125 |
| | 2 | 6 | 21,7 | 17,3 | 4 | 40 | Within | 1885,733 | 14 | 134,695 | | |
| | 3 | 1 | 10,0 | . | 10 | 10 | Total | 2537,529 | 16 | | | |
| | Total | 17 | 13,3 | 12,6 | 2 | 40 | | | | | | |
| PCR_12 | 1 | 10 | ,96 | 1,66 | ,1 | 5,6 | Between | 4,843 | 2 | 2,422 | ,622 | ,551 |
| | 2 | 6 | 2,05 | 2,44 | ,1 | 6,6 | Within | 54,486 | 14 | 3,892 | | |
| | 3 | 1 | 2,00 | . | 2,0 | 2,0 | Total | 59,329 | 16 | | | |
| | Total | 17 | 1,41 | 1,93 | ,1 | 6,6 | | 640,737 | 2 | 320,369 | 1,852 | ,193 |

Commento: Anova conferma una NON significativa influenza di TipoINF sulle suddette variabili

| Descrizione | | | | | | | Anova oneway | | | | | |
|---|---|---|---|---|---|---|---|---|---|---|---|---|
| | | | | | | | | | | | | |
| | CuraInf | N | Mean | SD | Min. | Max. | | Sum of sq | df | $Mean^2$ | F | Sig. |
| GLICEMIA_12 | 0=N | 8 | 87,6 | 7,4 | 82 | 105 | Between | 564,863 | 1 | 564,863 | 3,392 | ,085 |
| | 1=S | 9 | 99,1 | 16,3 | 72 | 131 | Within | 2497,608 | 15 | 166,507 | | |
| | total | 17 | 93,7 | 13,8 | 72 | 131 | Total | 3062,471 | 16 | | | |
| HBA1C_12 | 0=N | 8 | 37,3 | 4,0 | 31 | 42 | Between | ,944 | 1 | ,944 | ,045 | ,834 |
| | 1=S | 9 | 36,8 | 5,0 | 29 | 42 | Within | 313,056 | 15 | 20,870 | | |
| | total | 17 | 37,0 | 4,4 | 29 | 42 | Total | 314,000 | 16 | | | |
| COL TOT_12 | 0=N | 8 | 176,1 | 15,7 | 160 | 198 | Between | 135,334 | 1 | 135,334 | ,274 | ,608 |
| | 1=S | 9 | 181,8 | 26,6 | 155 | 234 | Within | 7402,431 | 15 | 493,495 | | |
| | total | 17 | 179,1 | 21,7 | 155 | 234 | Total | 7537,765 | 16 | | | |
| HLD_12 | 0=N | 8 | 47,5 | 15,2 | 30 | 77 | Between | 217,529 | 1 | 217,529 | ,787 | ,389 |
| | 1=S | 9 | 54,7 | 17,8 | 33 | 86 | Within | 4148,000 | 15 | 276,533 | | |
| | total | 17 | 51,3 | 16,5 | 30 | 86 | Total | 4365,529 | 16 | | | |
| LDL_12 | 0=N | 8 | 112,3 | 31,6 | 50 | 155 | Between | 40,265 | 1 | 40,265 | ,036 | ,852 |
| | 1=S | 9 | 115,3 | 35,1 | 80 | 180 | Within | 16863,500 | 15 | 1124,233 | | |
| | total | 17 | 113,9 | 32,5 | 50 | 180 | Total | 16903,765 | 16 | | | |
| TRIGLICERIDI_12 | 0=N | 8 | 98,1 | 37,6 | 47 | 149 | Between | 147,569 | 1 | 147,569 | ,133 | ,720 |
| | 1=S | 9 | 92,2 | 28,9 | 40 | 137 | Within | 16602,431 | 15 | 1106,829 | | |
| | total | 17 | 95,0 | 32,4 | 40 | 149 | Total | 16750,000 | 16 | | | |
| VES_12 | 0=N | 8 | 18,4 | 15,8 | 4 | 40 | Between | 390,099 | 1 | 390,099 | 2,725 | ,120 |
| | 1=S | 9 | 8,8 | 7,0 | 2 | 25 | Within | 2147,431 | 15 | 143,162 | | |
| | total | 17 | 13,3 | 12,6 | 2 | 40 | Total | 2537,529 | 16 | | | |
| PCR_12 | 0=N | 8 | 1,94 | 2,08 | ,1 | 6,6 | Between | 4,281 | 1 | 4,281 | 1,167 | ,297 |
| | 1=S | 9 | ,93 | 1,76 | ,1 | 5,6 | Within | 55,048 | 15 | 3,670 | | |
| | total | 17 | 1,41 | 1,93 | ,1 | 6,6 | Total | 59,329 | 16 | | | |

Commento: Anova conferma una NON significativa influenza di CuraInf sulle suddette variabili

Stratificazione dei pazienti in coloro che hanno al tempo 0 (baseline) PASI <5; 5≤ PASI <10 e PASI≥10 e valutare sulla base del PASI se vi è relazione con il sesso; età; comorbidità; la presenza di infezione ed eventualmente quale infezione.

**PASI2_0= 1(PASI<5); 2(5≤PASI<10); 3(PASI≥10)**

**Descriptives**

| Parameter | | Descrittiva | | | | | Anova | | | | | | Bonferroni p | | |
|---|---|---|---|---|---|---|---|---|---|---|---|---|---|---|---|
| | PASI2_0 | N | Mean | sd | min | max | | Sum | df | Mean$^2$ | F | Sig | PASI2 | 1 | 2 |
| **AGE** | 1 | 12 | 56,8 | 15,6 | 31 | 83 | Between | 1902,006 | 2 | 951,003 | 4,812 | **,011** | 2 | ,081 | |
| | 2 | 24 | 45,5 | 14,7 | 25 | 73 | Within | 13240,279 | 67 | 197,616 | | | 3 | 1,00 | **,015** |
| | 3 | 34 | 56,4 | 13,0 | 29 | 81 | Total | 15142,286 | 69 | | | | | | |
| | Total | 70 | 52,7 | 14,8 | 25 | 83 | | | | | | | | | |
| **ALTEZZA** | 1 | 12 | 170,6 | 9,2 | 160 | 190 | Between | 222,353 | 2 | 111,177 | 1,419 | ,249 | | | |
| | 2 | 24 | 174,9 | 9,4 | 150 | 195 | Within | 5251,132 | 67 | 78,375 | | | | | |
| | 3 | 34 | 171,4 | 8,4 | 157 | 190 | Total | 5473,486 | 69 | | | | | | |
| | Total | 70 | 172,5 | 8,9 | 150 | 195 | | | | | | | | | |
| **PESO_0** | 1 | 12 | 73,3 | 13,2 | 52 | 95 | Between | 168,134 | 2 | 84,067 | ,400 | ,672 | | | |
| | 2 | 24 | 77,7 | 16,2 | 50 | 130 | Within | 14080,566 | 67 | 210,158 | | | | | |
| | 3 | 34 | 77,2 | 13,7 | 57 | 114 | Total | 14248,700 | 69 | | | | | | |
| | Total | 70 | 76,7 | 14,4 | 50 | 130 | | | | | | | | | |
| **bmi0** | 1 | 12 | 25,10 | 3,40 | 19,57 | 30,49 | Between | 22,192 | 2 | 11,096 | ,508 | ,604 | | | |
| | 2 | 24 | 25,35 | 4,34 | 16,71 | 37,18 | Within | 1464,622 | 67 | 21,860 | | | | | |
| | 3 | 34 | 26,38 | 5,25 | 17,90 | 46,25 | Total | 1486,814 | 69 | | | | | | |
| | Total | 70 | 25,80 | 4,64 | 16,71 | 46,25 | | | | | | | | | |

Commento: Anova conferma esiste una differenza significativa dell'età tra strati di PASI2_0; 2 vs 3. Viceversa non esistono differenze significative tra strati per gli altri parametri.

**SEX1M * PASI2_0 Crosstabulation**

| | | PASI2_0 | | | | Chi-SquareTests | | | |
|---|---|---|---|---|---|---|---|---|---|
| sex | | 1 | 2 | 3 | Total | | Value | df | Sig. |
| 0=F | Count | 7 | 8 | 14 | 29 | Chi-Square | 2,062 | 2 | ,357 |
| | %sex | 24,1% | 27,6% | 48,3% | 100,0% | N | 70 | | |
| | %PASI2_0 | 58,3% | 33,3% | 41,2% | 41,4% | | | | |
| 1=M | Count | 5 | 16 | 20 | 41 | Commento: il test certifica che le occorrenze di genre NON risultano statisticamente diverse tra strati di PAS2_0 | | | |
| | %sex | 12,2% | 39,0% | 48,8% | 100,0% | | | | |
| | %PASI2_0 | 41,7% | 66,7% | 58,8% | 58,6% | | | | |
| Total | Count | 12 | 24 | 34 | 70 | | | | |
| | %sex | 17,1% | 34,3% | 48,6% | 100,0% | | | | |
| | %PASI2_0 | 100,0% | 100,0% | 100,0% | 100,0% | | | | |

**COMORBIDITA * PASI2_0 Crosstabulation**

| | | PASI2_0 | | | | Chi-SquareTests | | | |
|---|---|---|---|---|---|---|---|---|---|
| Comorbidità | | 1 | 2 | 3 | Total | | Value | df | Sig. |
| 0=N | Count | 4 | 11 | 8 | 23 | Chi-Square | 3,174 | 2 | ,205 |
| | %Comorbidità | 17,4% | 47,8% | 34,8% | 100,0% | N | 70 | | |
| | %PASI2_0 | 33,3% | 45,8% | 23,5% | 32,9% | | | | |
| 1=S | Count | 8 | 13 | 26 | 47 | Commento: il test certifica che le occorrenze di COMORBIDITA' NON risultano statisticamente diverse tra strati di PAS2_0 | | | |
| | %Comorbidità | 17,0% | 27,7% | 55,3% | 100,0% | | | | |
| | %PASI2_0 | 66,7% | 54,2% | 76,5% | 67,1% | | | | |
| Total | Count | 12 | 24 | 34 | 70 | | | | |
| | %Comorbidità | 17,1% | 34,3% | 48,6% | 100,0% | | | | |
| | %PASI2_0 | 100,0% | 100,0% | 100,0% | 100,0% | | | | |

**Infezione1S * PASI2_0 Crosstabulation**

| | | PASI2_0 | | | |
|---|---|---|---|---|---|
| **Infezione1S** | | 1 | 2 | 3 | Total |
| 0=No | Count | 7 | 15 | 15 | 37 |
| | % Infezione1S | 18,9% | 40,5% | 40,5% | 100,0% |
| | %PASI2_0 | 58,3% | 62,5% | 44,1% | 52,9% |
| 1=Si | Count | 5 | 9 | 19 | 33 |
| | % Infezione1S | 15,2% | 27,3% | 57,6% | 100,0% |
| | %PASI2_0 | 41,7% | 37,5% | 55,9% | 47,1% |
| Total | Count | 12 | 24 | 34 | 70 |
| | % Infezione1S | 17,1% | 34,3% | 48,6% | 100,0% |
| | %PASI2_0 | 100,0% | 100,0% | 100,0% | 100,0% |

| Chi-SquareTests | | | |
|---|---|---|---|
| | Value | df | Sig. |
| Chi-Square | 2,082 | 2 | ,353 |
| N | 70 | | |

Commento: il test certifica che le occorrenze di Infezioni NON risultano statisticamente diverse tra strati di PAS2_0
